# Design, synthesis, and biological evaluation of triazole-pyrimidine-methylbenzonitrile derivatives as dual A_2A_/A_2B_ adenosine receptor antagonists

**DOI:** 10.1080/14756366.2022.2077731

**Published:** 2022-05-26

**Authors:** Zhi Li, Lijuan Kou, Xinzhen Fu, Zeping Xie, Maolei Xu, Lin Guo, Tiantian Lin, Shizhou Gong, Shumin Zhang, Ming Liu

**Affiliations:** a School of Pharmacy, Binzhou Medical University, Yantai, China; b Luye Pharma Group, Yantai, China

**Keywords:** Dual A_2A_/A_2B_ adenosine receptor antagonists, quinoline, methylbenzonitrile, T cell activation, pharmacokinetics

## Abstract

A series of novel dual A_2A_/A_2B_ AR antagonists based on the triazole-pyrimidine-methylbenzonitrile core were designed and synthesised. The A_2A_ AR antagonist cAMP functional assay results were encouraging for most target compounds containing quinoline or its open-ring bioisosteres. In addition, compound **7i** displayed better inhibitory activity on A_2B_ AR (IC_50_ 14.12 nM) and higher potency in IL-2 production than AB928. Moreover, molecular docking studies were carried out to explain the rationality of molecular design and the activity of compound **7i**. Further studies on **7f** and **7i** revealed good liver microsomes stabilities and acceptable *in vivo* PK profiles. This study provides insight into the future development of dual A_2A_/A_2B_ AR antagonists for cancer immunotherapy.

## 1. Introduction

Adenosine is one of the most important signalling molecules in the human body, and it exerts its effects through G-protein coupled receptors, including A_1_, A_2A_, A_2B_, and A_3_ adenosine receptors (ARs)^1–3^. Upon activation by adenosine, A_2A_ AR and A_2B_ AR promote adenylyl cyclase (AC) activation and subsequent cyclic AMP (cAMP) production^4^^,^^5^. Elevated intracellular cAMP in T cells will result in T cell anergy by reducing its proliferation, maturation, cytokine production (e.g., IL-2), and tumour-killing activity^6–8^. The cell cytotoxicities of natural killer cells, dendritic cells, or macrophages are inhibited by this pathway as well^9–11^. In the tumour microenvironment (TME), the level of extracellular adenosine is higher than that of normal tissue, leading to immune evasion^4^^,^^12^^,^^13^. A_2A_ and A_2B_ ARs are widely considered critical to the immune functions of adenosine. The relevance of A_2_ receptors in tumour immunotherapy has stimulated the development of various selective antagonists for these receptors^14–19^.

**Figure 1.**
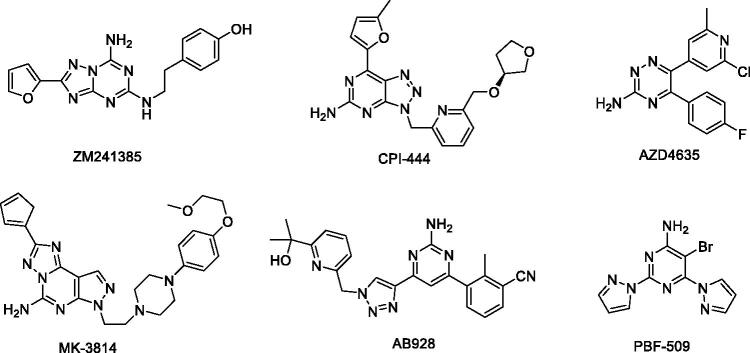
Chemical structures of A_2_ adenosine receptor antagonists.

In recent years, the discovery and translation of A_2_ AR antagonists from the bench to bedside for cancer immunotherapy have made significant progress, with some selective A_2_ AR antagonists entering clinical trials either alone or in combination with other immunotherapies (Figure 1)^1^^,^^20–23^. Previous studies mostly focussed on the discovery of inhibitors against the A_2A_ receptor. ZM241385 (Figure 1) is a very potent and selective A_2A_ AR antagonist developed by the AstraZeneca Group^24^. It can inhibit and delay tumour growth significantly^25^. The structural analysis of A_2A_ AR bound to ZM241385 confirmed the π–π stacking interaction with Phe168 and hydrogen bonds (H bonds) with Asn253 established by inhibitors were beneficial to improving the binding ability to the A_2A_ receptor, and this finding has facilitated the discovery of novel AR antagonists^26–29^. For example, the A_2A_ AR inhibitor CPI-444 (Figure 1) developed by Corvus Pharmaceuticals is considered to have a similar target binding form to ZM241385 and it is currently in a phase 1b/2 trial for the treatment of renal cell cancer^1^^,^^2^^,^^30^.

**Figure 2.**
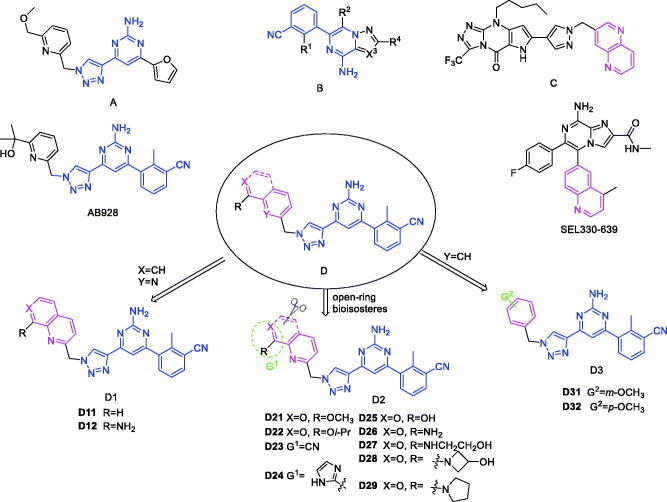
Design of dual A_2A_/A_2B_ AR antagonists containing quinoline or its open-ring bioisosteres based on the structure of triazole-pyrimidine-methylbenzonitrile.

Since the A_2B_ receptor is also important in adenosine signal transduction, especially in cells of myeloid origin, a dual A_2A_/A_2B_ AR antagonist usually exhibits better inhibition effects^9^^,^^18^^,^^31^^,^^32^. Azolopyrimidines (e.g., compound A in Figure 2) were first disclosed by Arcus Biosciences as A_2A_R antagonists^33^. However, AB928 contains methylbenzonitrile, instead of the furan fragment of compound A, and it can inhibit A_2A_ AR and A_2B_ AR with similar potencies and outperform others in preclinical testing^22^. In 2019, Selvita Group published a patent on imidazo[1,2-a]pyrazines for the treatment of tumour-related disorders^34^. Among these imidazo[1,2-a]pyrazines, SEL330-639 containing the quinoline structure was confirmed to be a dual A_2A_/A_2B_ receptor antagonist with nanomolar potency^9^. Through the structure–activity relationship of the above two compounds, methylbenzonitrile and quinoline structures may be advantageous scaffolds to obtain potent dual A_2A_/A_2B_ AR antagonists. Incyte Corporation developed two types of A_2A_/A_2B_ inhibitors (Figure 2, compounds B and C)^35^^,^^36^. Compounds containing benzonitrile structure or quinoline analogues also showed good inhibitory activities on both of these two receptors. Thus, we introduced quinoline or its open-ring bioisosteres to the structure of AB928 to develop new dual A_2A_/A_2B_ AR antagonists. Herein, a series of novel A_2_ AR antagonists containing triazole-pyrimidine-methylbenzonitrile core were designed and synthesised (Figure 2, compounds D). Their potential inhibitory activities to A_2A_ or A_2B_ ARs were further investigated by cAMP functional assay and T cell activation assay. In addition, the representative compound **7i** was subjected to molecular docking studies to reveal the binding behaviour. Moreover, microsomal metabolic stability and *in vivo* pharmacokinetic properties were assessed for optimised compounds.

## 2. Results and discussion

### 2.1. Chemistry

**Scheme 1.**
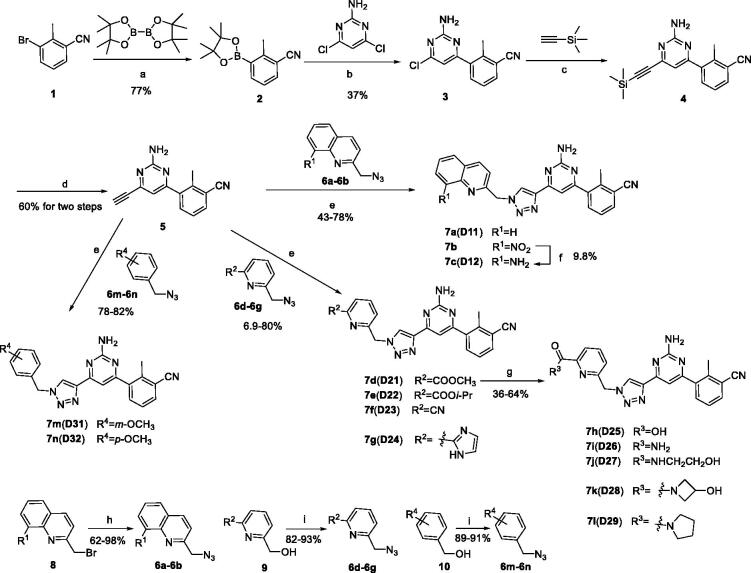
Synthetic route of target compounds **7a**–**7n**. Reagents and conditions: (a) Pd(dppf)Cl_2_, (Bpin)_2_, potassium acetate, 1,4-dioxane, N_2_, reflux, 4 h; (b) 4,6-dichloropyrimidin-2-amine, K_2_CO_3_, Pd(PPh_3_)_4_, DMF, N_2_, 45 °C, 5 min, then compound **2** was added, 115 °C, 5 h; (c) Et_3_N, TMSA, Pd(PPh_3_)_2_Cl_2_, CuI, dry THF, N_2_, reflux, 16 h; (d) TBAF(1M in THF), THF, 0 °C to r.t., 20 h; (e) Compound **6**, CuSO_4_·5H_2_O, sodium l-ascorbate, 60 °C, DMF, *t*-BuOH, 12 h; (f) SnCl_2_, 70 °C, 2 h; (g) for **7 h**: LiOH, H_2_O, *t*-BuOH, r.t., 8 h; for **7i**–**7l**: The corresponding amine derivatives, MeOH, THF, 45 °C, 18 h; (h) TMSiA, DIPEA, THF, r.t., 24 h; (i) DPPA, DBU, THF, r.t., 10 h.

The synthetic route of target compounds **7a**–**7l** is shown in Scheme 1. Compound **2** was synthesised from the reaction of 3-bromo-2-methylphenylacetonitrile (**1**) with (BPin)_2_ catalysed by Pd(dppf)Cl_2_. Then, compound **3** was acquired from the Suzuki coupling reaction between compound **2** and 4,6-dichloro-2-pyrimidinamine. Subsequently, compound **4** was synthesised via the Sonogashira cross-coupling reaction of compound **3** with trimethylsilylacetylene (TMSA), which further reacted with tetrabutylammonium fluoride (TBAF) in THF to get compound **5**. A copper and sodium l-ascorbate catalysed azide-alkyne coupling between **5** and **6** yielded compounds **7a**–**7b** and **7d**–**7g**. In addition, compound **7b** could be reduced with SnCl_2_ to obtain compound **7c**. Furthermore, treatment of compound **7d** with LiOH aqueous solution or various amine derivatives yielded **7h**–**7l**. The synthesis of intermediates **6** can be achieved by nucleophilic substitution of the corresponding alcohols or bromides with azidotrimethylsilane (TMSiA) or diphenyl azidophosphate (DPPA) with good yields (the synthesis of intermediates **6** can be found in the Supporting information).

**Scheme 2.**
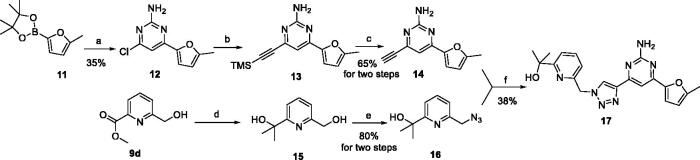
Synthetic route of the target compound **17**. Reagents and conditions: (a) 4,6-dichloropyrimidin-2-amine, K_2_CO_3_, Pd(PPh_3_)_4_, DMF, N_2_, 45 °C, 5 min, then compound **11** was added, 115 °C, 5 h; (b) Et_3_N, TMSA, Pd(PPh_3_)_2_Cl_2_, CuI, dry THF, N_2_, reflux, 16 h; (c) TBAF(1M in THF), THF, 0 °C to r.t., 20 h; (d) MeMgBr, THF, −10 °C to r.t., 12 h; (e) DPPA, DBU, THF, r.t., 10 h; (f) CuSO_4_·5H_2_O, sodium l-ascorbate, 60 °C, DMF, *t*-BuOH, 12 h.

**Scheme 3.**
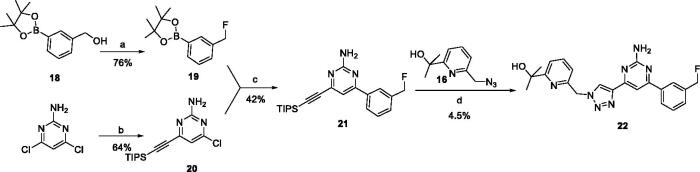
Synthetic route of the target compound **22**. Reagents and conditions: (a) MsCl, Et_3_N, 0 °C, DCM, 1 h, then TBAF (1 M in THF) was added, 50 °C, 12 h; (b) 4,6-dichloropyrimidin-2-amine, TMSA, Et_3_N, Pd(PPh_3_)_2_Cl_2_, CuI, dry THF, N_2_, 80 °C, 12 h; (c) Pd(dppf)Cl_2_, K_2_CO_3_, 1,4-dioxane, N_2_, 100 °C, 4 h; (d) TBAF (1 M in THF), CuSO_4_·5H_2_O, sodium l-ascorbate, H_2_O, *t*-BuOH, 60 °C,12 h.

To verify the effect of the benzonitrile and quinoline structure on A_2_ receptors, compounds **7m**, **7n**, **17**, and **22** were synthesised following the synthetic routes shown in Scheme 1–3. Compounds **7m**–**7n**, which contain a benzene ring instead of the quinoline structure, were synthesised by the azide-alkyne coupling between **5** and **6m**–**6n** in 78–82% yield. The synthesis of compound **17** (Scheme 2) started from 5-methylfuran-2-boronic acid pinacol ester (**11**), according to the synthesis method of compound **7a**, and **17** was generated in moderate yield. In the synthetic route of compound **22**, the intermediate **21** synthesised from compound **18** can be purchased from Sundia Company (the synthesis of intermediate **21** is found in the Supporting information). Intermediate **21** then reacted with TBAF and intermediate **16** in the presence of CuSO_4_·5H_2_O and sodium l-ascorbate to obtain target compound **22** in a one-pot reaction.

### 2.2. Biology studies

#### 2.2.1. cAMP functional assay

In the hypoxic TME, over-activation of A_2_ receptors upregulates cAMP levels by stimulating adenylate cyclase. The abilities of synthesised compounds **7**, **17**, and **22** to inhibit the hA_2A_ or hA_2B_ receptor were studied by evaluating their effect on cAMP production in Chinese hamster ovary (CHO) cells which stably express hA_2A_ AR or hA_2B_ AR^4^^,^^9^^,^^37^.

The cAMP assay results are summarised in Table 1. In the cAMP assay, CHO-K1/ADORA2A/Gα15 cells expressing human A_2A_ AR and CHO-K1/ADORA2b/Gα15 cells expressing human A_2B_ AR were activated by 5′-(N-ethylcarboxamido)adenosine (NECA) and triggered the accumulation of intracellular cAMP concentration. The agonist-induced cAMP intracellular accumulation was inhibited by compounds **7** or ZM241385. The inhibition rates of compounds **7** were determined by comparing the inhibitory activities of the target compounds at a certain concentration with that of ZM241385 at 1 μM. From the cAMP assay results, compounds **7** exhibited good to excellent inhibitory activities on the NECA-triggered cAMP intracellular accumulation in CHO cells expressing human A_2A_ AR (from 43.54% to 107.32%) at 1 μM. It was noted that **7g** and **7i** showed superior inhibition activity to AB928, as a control. At 100 nM, compound **7a** containing quinoline structure showed 16.52% and 3.51% inhibition, respectively, on A_2A_ AR- and A_2B_ AR-mediated cAMP production. It is worth mentioning that the introduction of an amino group as a hydrogen bond donor at C8 of the quinoline moiety was beneficial to the inhibitory activities (Table 1, **7a** versus **7c**), In particular, the inhibition of A_2B_ AR-mediated cAMP production was increased to an impressive 85.43%. Moreover, the replacement of the phenyl ring of the quinoline structure by a ring-opening structure further increased the activity. Compounds **7d**–**7l**, containing the pyridine structure, exhibited better inhibitory activities against A_2A_ AR than compound **7c** (from 50.68% to 95.93%) at 100 nM, among them, **7f** and **7i** showed 84.21% and 95.93% inhibition, respectively, which were equivalent to that of AB928. Interestingly, at lower concentration (10 nM), they displayed higher inhibition rates than AB928. In addition, the inhibitory activities of **7f** and **7i** on A_2B_ AR-mediated cAMP production were comparable to that of the control compound AB928.

**Table 1.** Antagonist activity (%inhibition) of compounds **7**, **17**, and **22** at 10–1000 nM on cAMP levels in CHO cells expressing hA_2A_ AR or hA_2B_ AR ^a^. 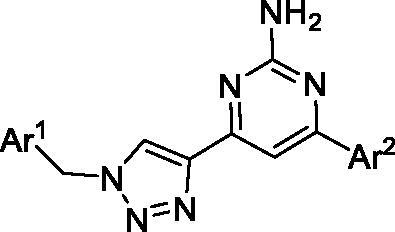

Compound	**Ar** ^1^	**Ar** ^2^	hA_2A_ AR (%)			hA_2B_ AR (%)
			1000 nM	100 nM	10 nM	100 nM
**7a**	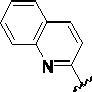	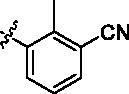	43.54 ± 0.77	16.52 ± 1.90	28.24 ± 8.79	3.51 ± 3.19
**7c**	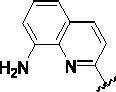	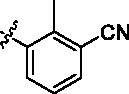	48.45 ± 1.45	38.16 ± 10.85	35.9 ± 5.39	85.43 ± 0.04
**7d**	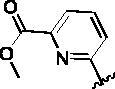	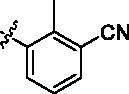	91.27 ± 4.28	63.38 ± 1.39	40.83 ± 9.01	80.27 ± 1.84
**7e**	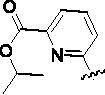	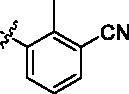	67.76 ± 4.91	50.68 ± 5.51	44.99 ± 1.25	82.45 ± 7.81
**7f**	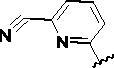	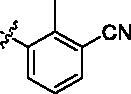	92.41 ± 0.43	84.21 ± 2.94	54.91 ± 1.19	102.64 ± 0.24
**7g**	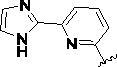	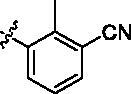	107.32 ± 0.89	56.02 ± 2.03	19.61 ± 2.13	52.94 ± 3.96
**7h**	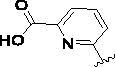	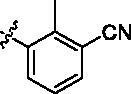	92.61 ± 1.91	55.18 ± 4.27	37.91 ± 2.32	54.75 ± 2.09
**7i**	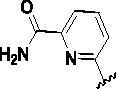	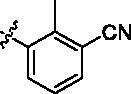	102.58 ± 2.82	95.93 ± 3.36	32.73 ± 1.80	103.33 ± 0.63
**7j**	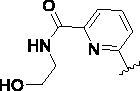	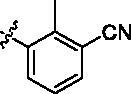	100.79 ± 4.62	82.17 ± 1.26	22.45 ± 6.68	82.38 ± 4.52
**7k**	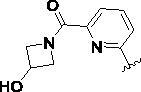	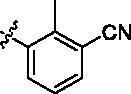	94.87 ± 2.05	70.52 ± 6.37	21.69 ± 5.43	78.28 ± 2.66
**7l**	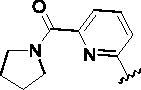	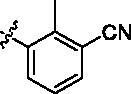	89.66 ± 1.31	52.3 ± 8.46	13.46 ± 3.71	84.34 ± 4.72
**7m**	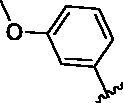	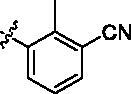	69.63 ± 1.01	28.64 ± 4.03	21.51 ± 4.32	69.38 ± 4.01
**7n**	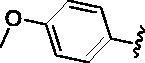	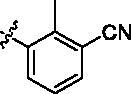	54.77 ± 2.41	29.78 ± 0.38	18.77 ± 3.88	63.52 ± 4.93
**17**	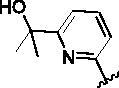	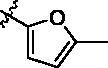	92.65 ± 2.87	92.28 ± 0.61	17.63 ± 2.78	64.42 ± 0.89
**22**	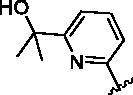	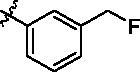	93.61 ± 2.76	94.73 ± 4.43	22.39 ± 11.87	19.88 ± 1.85
AB928^b^	AB928	AB928	101.35 ± 5.55	96.8 ± 7.16	20.04 ± 6.46	102.85 ± 1.04

^a^Data are expressed as means ± SEM.

^b^Used as a positive control.

The activities of compounds **7d**–**7l** at 100 nM indicate the substituents at the pyridine structure could influence the inhibitory activities of these compounds on both A_2_ ARs. The substituent should be a group that contains a hydrogen bond receptor such as an ester group, cyano group, or amide (**7d**–**7f**, and **7l**). A hydrogen bond donor such as –NH_2_ and –NH– could also increase the inhibition activities of structures bearing carbonyl group (**7d**–**7e** versus **7i**–**7j**) on both A_2A_ AR and A_2B_ AR. For compounds with an amide moiety, the size of the substituent at the nitrogen atom has a negative influence on the activity (**7i **>** 7j **>** 7k**). A hydrogen bond donor attached to the carbonyl group was necessary for the inhibitory activities of target compounds on A_2A_ AR, but it has no obvious effect on the inhibitory activities on A_2B_ AR (**7d**–**7e**, and **7l**).

To further validate our design strategy, compounds **7m**–**7n**, containing a benzene ring instead of the pyridine ring or quinoline structure, were synthesised. These compounds exhibited similar inhibitory activity to compounds **7a** and **7c** at concentrations of 1000 nM and 100 nM, but the activities were lower at 10 nM. Moreover, compounds **17** and **22** containing the same Ar^1^ fragment as AB928 were synthesised and showed good inhibitory activities at 10 nM, 100 nM, and 1000 nM against A_2A_ AR. However, their activities against A_2B_ AR decreased significantly at 100 nM because of the replacement of the Ar^2^ fragment by other structures. The changes in the activities of compounds **7m**–**7n**, **17**, and **22** relative to **7a**, **7c**–**7l**, and AB928 indicated the structures of benzonitrile and pyridine analogues are beneficial to improving the inhibitory activities of target compounds on human A_2_ receptor, which was consistent with our design strategy.

We further tested the IC_50_ values of compounds **7f**, **7i**, **17**, **22**, and AB928 in inhibiting the cAMP production of CHO cells expressing hA_2A_ AR or hA_2B_ AR (Table 2). The results indicate the inhibitory activity of compound **7i** on the A_2A_ receptor is similar to that of AB928, and compound **7f** is 4-fold less active than compound **7i**. The inhibitory activities of compounds **17** and **22** were 52-fold and 11-fold less active than compound **7i**, respectively. In addition, the competitive binding experiments of compounds **7f**, **7i**, and AB928 were performed using membrane preparation of HEK-293 cells expressing human A_2A_ AR (Table 3). Their IC_50_ values were 158.0 nM, 63.55 nM, and 25.23 nM, which are consistent with the results of the cAMP assay.

**Table 2.** IC_50_ values of selected compounds on cAMP assays in CHO cells expressing hA_2A_ or hA_2B_ AR^a^.

Compound	cAMP IC_50_ (nM)	
	hA_2A_ AR	hA_2B_ AR
**7f**	24.04 ± 0.06	102.64 ± 0.24%^b^, 10.79 ± 0.67%^c^
**7i**	6.007 ± 0.05	14.12 ± 2.05
**17**	320.5 ± 94.04	80.03 ± 13.45
**22**	67.19 ± 10.92	178.5 ± 9.89
AB928	2.754 ± 0.12	25.48 ± 0.35

^a^Data are expressed as means ± SEM.

^b^Percentage of inhibition (I%) determined at 100 nM concentration of compound **7f**.

^c^Percentage of inhibition (I%) determined at 10 nM concentration of compound **7f**.

**Table 3.** Radioligand binding affinity data (IC_50_) for selected compounds against hA_2A_ AR^a^.

Compound	cAMP assays IC_50_ (nM)	Radioligand binding assays IC_50_ (nM)
**7f**	24.04 ± 0.06	158.0 ± 56.42
**7i**	6.007 ± 0.05	63.55 ± 13.03
AB928	2.754 ± 0.12	25.23 ± 0.74

^a^Data are expressed as means ± SEM.

Compound **7i** was also the most effective in inhibiting A_2B_ AR-mediated cAMP production, and the IC_50_ value was 14.12 nM, which is 1.8-fold more active than compound AB928. Compounds without benzonitrile structure (**17** and **22**) had poor inhibitory activities indicating the cyano structure on the benzene ring is closely related to the inhibitory activity of the antagonist on A_2B_ AR.

#### 2.2.2. T Cell activation assay

**Figure 3.**
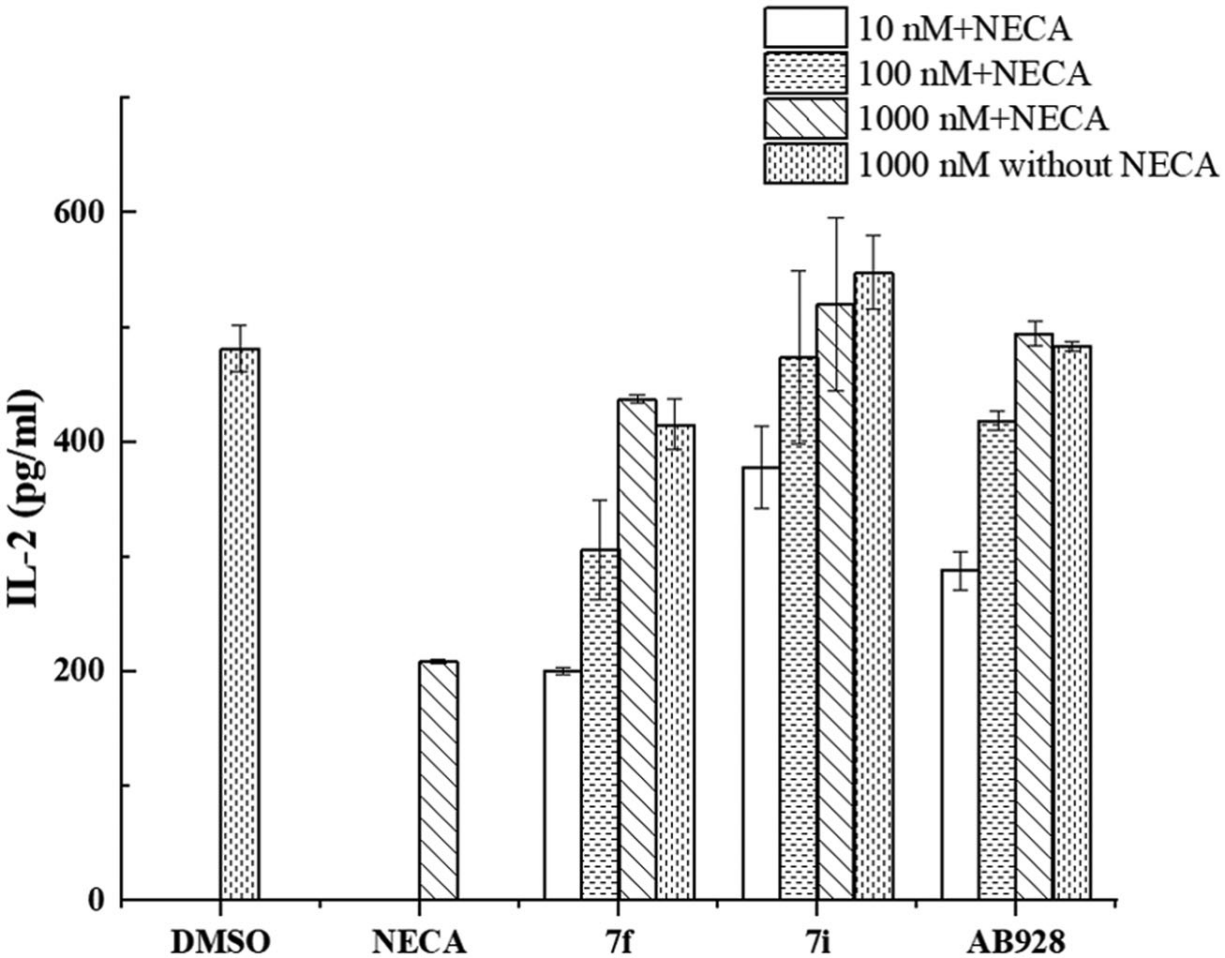
The efficiencies of selected compounds for IL-2 production. The efficiencies of selected antagonists on T-cell activation at 10 nM, 100 nM, and 1000 nM were tested in the presence or absence of 1000 nM NECA. The DMSO control was tested in the absence of both antagonists and NECA. The NECA control was tested without antagonists in the presence of 1000 nM NECA.

The effector functions of T cells that express A_2_ ARs can be evaluated by measuring specific cytokine production^4^^,^^9^^,^^38^. In this study, the efficiencies of selected antagonists on IL-2 production (T cell activation) were examined. Two compounds (**7f** and **7i**) were evaluated in this assay and showed sufficient activities to stimulate IL-2 production through T cell activation (Figure 3). The results show the concentration of IL-2 was significantly reduced in the NECA control, which suggests NECA inhibited T cell activation. However, when the antagonist was added, the concentration of IL-2 was restored, indicating the inhibition effect of NECA was blocked. Interestingly, **7i** demonstrated better activity than AB928, suggesting it has potential as an immunotherapy against tumours via T cell activation.

#### 2.2.3. Molecular docking studies

**Figure 4.**
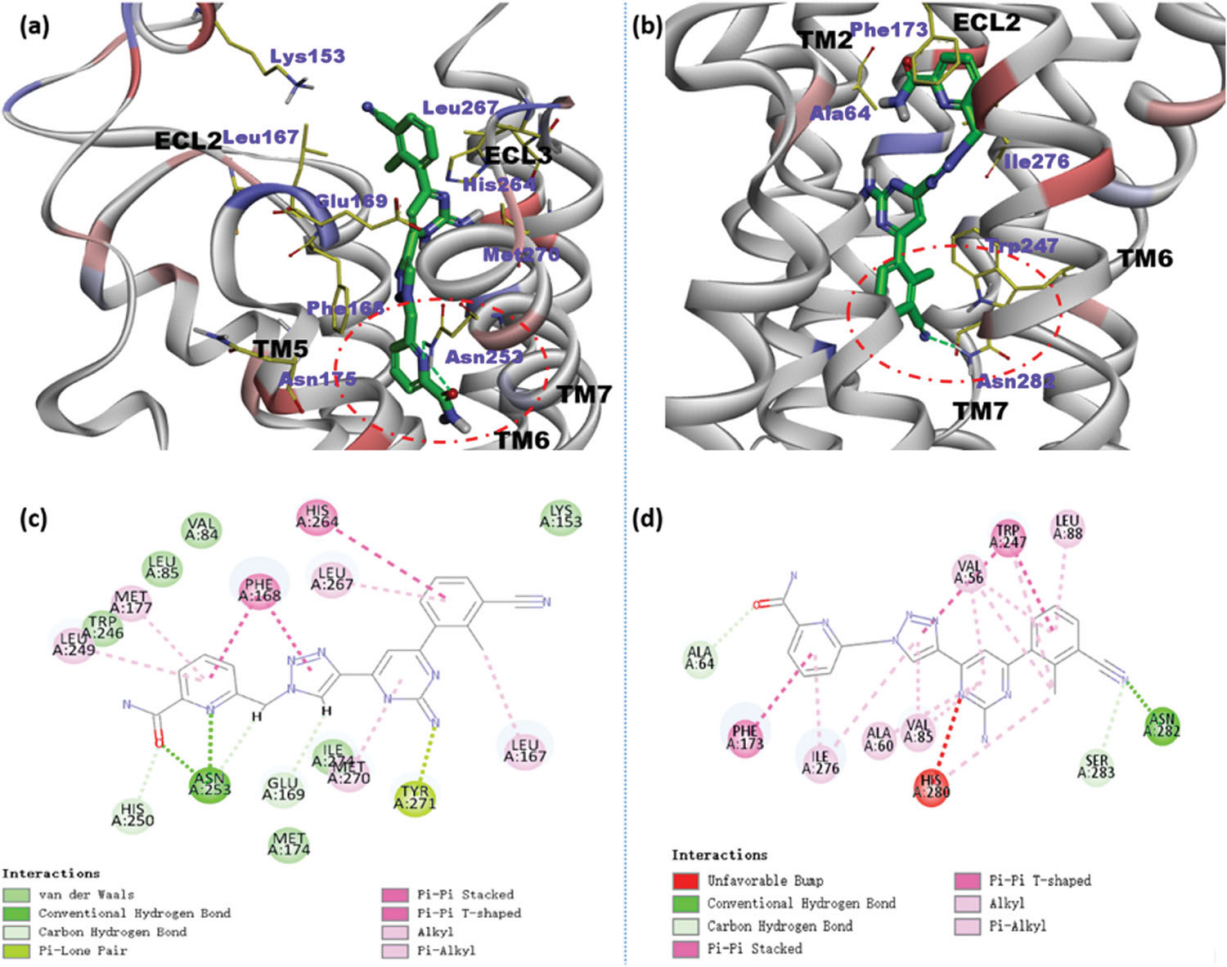
Binding modes of compound **7i** at A_2A_ AR (a) and hA_2B_ AR (b) binding cavities have indicated docking orientations and some key receptor residues. Depths of the two binding pockets have been circled with the red dotted line. 2D diagrams of ligand-target interactions for docking compound **7i** with the A_2A_ AR (c) and hA_2B_ AR (d) predicted by Discovery Studio.

Docking studies were performed at the hA_2A_ and hA_2B_ AR binding sites to simulate the interaction of compound **7i** with these two receptors. The binding modes of compound **7i** at the hA_2A_ AR cavity were analysed by docking simulations using the C-DOCKER protocol of Discovery Studio 2017 R2^39^. The crystal structure of hA_2A_ AR in complex with antagonist ZM241385 (PDB ID: 5IU4) was retrieved from the Protein Data Bank (http://www.rcsb.org/pdb) for the docking calculations. The docking simulation results show that compound **7i** could bind to the active site inside the transmembrane (TM) region and extracellular loops (ECLs) of the human A_2A_ AR similar to the co-crystallized ZM241385^27^. In this binding mode, the phenyl group is located in the proximity of His264, Leu167, Leu267, and Lys153 at the entrance of the hA_2A_ AR cavity (Figure 4(a,c); for more details see supporting information Figure S2). The pyridine and triazole scaffold position in the depth of the binding pocket and both give π–π interaction with the key receptor residue Phe168 (ECL2). This indicates the attachment of an aromatic ring to the triazole facilitates the binding of the inhibitor to the target. In addition, the asparagine residue in TM6 (Asn253) forms H-bond with the N1 atom at the pyridine scaffold and it forms an additional H-bond with the amide group attached to the pyridine ring. The docking results are consistent with the biological results.

The binding mode of hA_2B_ AR was also analysed with the same docking protocols in Discovery Studio 2017 R2. The homology model of hA_2B_ AR was developed using a hA_2A_ AR crystal structure as the template (PDB ID: 6PS7) and has been checked using the Ramachandran plot application within Discovery Studio^40^^,^^41^. In the binding mode (Figure 4(b,d); for more details see Supporting information Figure S3), the pyridine scaffold is located at the entrance of the cavity getting close to residue Phe173 in the ECL2 segment, and the methylbenzonitrile moiety is located in the depth of the binding pocket by a π–π interaction with Trp247. In addition, the cyano group has a polar interaction with the amide function of Asn282. The binding mode shows the methylbenzonitrile structure is beneficial to obtaining potent inhibitory activity on A_2B_ AR. Moreover, favourable interactions with the amide group and methyl group are established with the side chains of Ala64 and His280. Thus, the substituents on the pyridine ring may affect the compound affinity.

#### 2.2.4. Microsomal metabolic stability

The metabolic stability is an important focus for further compound optimisation. Compounds **7f**, **7i**, and AB928 were chosen to evaluate the stability in rat liver microsomes and human liver microsomes. For the microsomal metabolic experiment, 7-ethoxycoumarin (7-EC) was used as a control. As shown in Table 4 (see Supporting information for raw data), the CL_int(liver)_ of 7-EC in rats and humans was 10.0 and 9.0 ml/min/g liver, respectively, indicating the experimental test system was reliable. Compounds **7f** and **7i** exhibited good liver microsomes stabilities *in vitro*. They hardly metabolised on human liver microsomes but showed slow metabolism on rat microsomes with CL_int(liver)_ of 0.78 and 1.08 ml/min/g, and both compounds were more stable than AB928 (2.15 ml/min/g in rat liver microsomes).

**Table 4.** Metabolic stability assay in rat and human liver microsomes^a^.

Compound	Microsomal metabolic stability					
	*T*_1/2_ (min)		CL_int(liver)_(mL/min/g)		Remaining (%, *T* = 60min)	
	Hum	Rat	Hum	Rat	Hum	Rat
**7f**	n.c.	93.65	n.c.	0.78	104.65	64.04
**7i**	1155	67.28	0.06	1.08	98.76	50.67
AB928	433.13	33.8	0.17	2.15	94.40	28.10
7-EC	8.12	7.26	9.0	10.0	0.72	0.35

^a^*T*_1/2_, half-life, CL_int(liver)_, intrinsic clearance, n.c., not calculated.

#### 2.2.5. *In vivo* pharmacokinetics study

**Figure 5.**
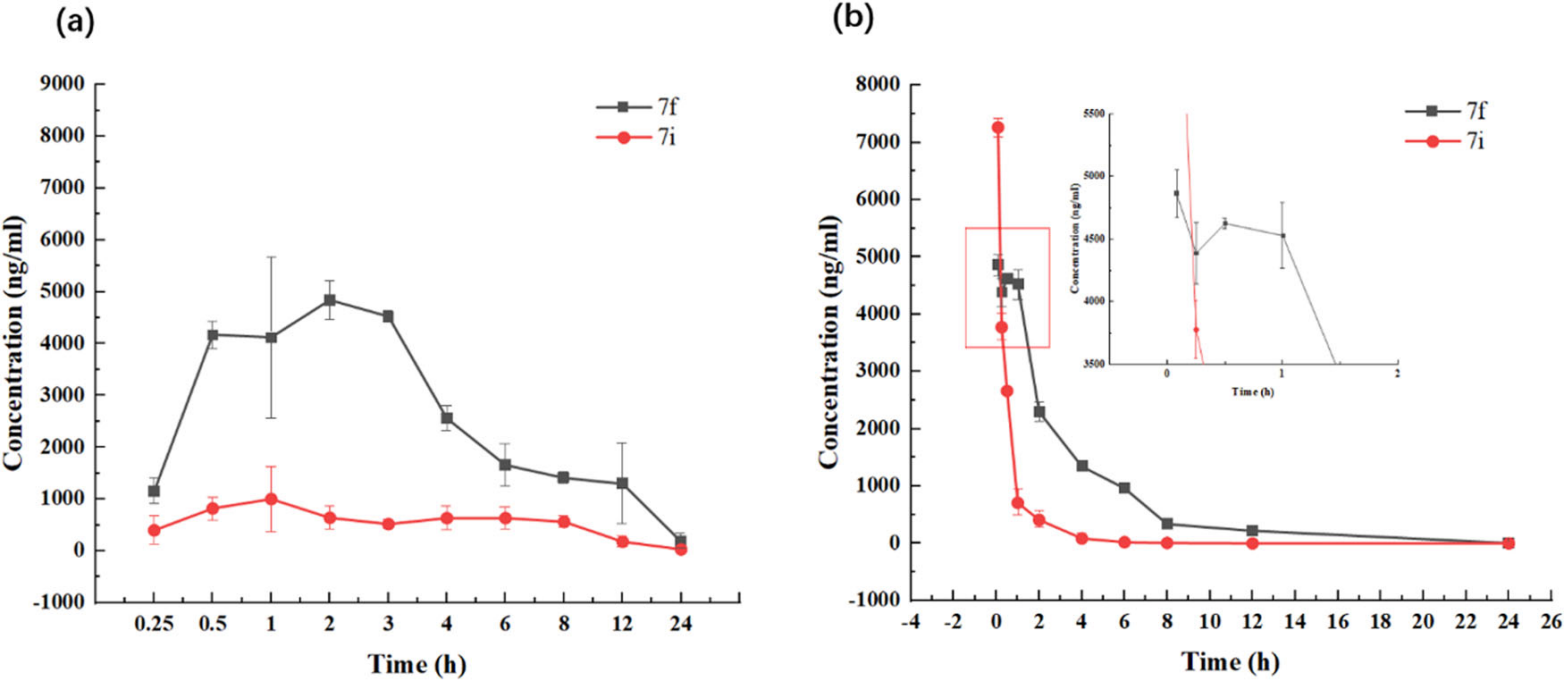
Plasma concentration-time profiles of compounds **7f** and **7i** following oral administration (a) and intravenous administration (b).

The *in vivo* pharmacokinetic (PK) profiles of compounds **7f** and **7i** were examined in male Balb/c mice (Table 5 and Figure 5). The two compounds showed similar pharmacokinetic properties. After the intravenous (i.v.) administration of 10 mg/kg, the maximum plasma concentration of compounds **7f** and **7i** were 5010.30 µg/l at 0.25 h and 7266.60 µg/l at 0.25 h, respectively. When orally administered at 30 mg/kg, these compounds showed moderate half-lives at a range of 4.12–4.82 h. The absolute bioavailabilities of compounds **7f** and **7i** were 87.7% and 58.8%, respectively. These PK parameters can be used to evaluate the drug-like properties of compounds **7f** and **7i**, so as to clarify their effectiveness and obtain better clinical treatment effects.

**Table 5.** Pharmacokinetic parameters of compounds **7f** and **7i** ^a^.

Parameter	7f		7i	
	Oral	i.v.	Oral	i.v.
*t*_1/2_ (h)	4.12	2.67	4.82	2.19
*t*_max_(h)	2.00	0.25	1.00	0.08
*C*_max_(µg/L)	7162.80	5010.30	1007.40	7266.60
*C*_max/Dose_^b^	238.76	501.03	33.58	726.66
AUC_0→t_(µg/L*h)	45,490.39	1728.68	7904.90	4484.58
AUC_0→t/Dose_^c^	1516.35	1728.68	263.50	448.46
AUC_0→∞_(µg/L*h)	46,232.45	17315.97	8145.18	4484.81
AUC_0→∞/Dose_^c^	1541.08	1731.60	271.51	448.48
F(%)	87.7		58.8	

^a^PK parameters (mean, *n* = 3), *C*_max_, maximum concentration; *T*_max_, time to reach *C*_max_; AUC_0→t_, Area under the curve from zero to the last measurable plasma concentration; AUC_0→∞_, Area under the curve from the last measurable plasma concentration to infinity.

^b^Unit is (µg/L)/(mg/kg).

^c^Unit is (µg/L*h)/(mg/kg).

## 3. Conclusion

A series of dual A_2A_/A_2B_ AR antagonists containing structures of methylbenzonitrile and quinoline or pyridine analogues were designed and synthesised. *In vitro* cAMP assays of target compounds on A_2A_ AR and A_2B_ AR showed good to excellent inhibitory activities. Among these compounds, the inhibitory activity on the A_2A_ receptor of compound **7i** approached that of AB928. Additionally, compound **7i** displayed better inhibitory activity on A_2B_ AR and higher potency in IL-2 production than AB928. Further studies on **7i** demonstrated good liver microsomes stabilities and acceptable *in vivo* PK properties. In future studies, we aim to further improve the potency and drug-like parameters of target compounds. Further optimizations of compound **7i** are still in progress.

## 4. Experimental section

### 4.1. General methods for chemistry

All chemicals were purchased from commercial suppliers and used without further purification. Air or moisture sensitive reactions were performed under a positive pressure of nitrogen with oven-dried glassware. Reactions were monitored by thin layer chromatography (TLC), and spots were visualised with iodine vapour or by irradiation with UV light. Flash column chromatography was performed using the Qingdao Haiyang flash silica gel (200–300 mesh). All yields were reported as isolated yields. The melting points were determined on an X-4 binocular microscope melting point apparatus (Beijing Tech Instruments Co., Beijing, China) and were uncorrected. ^1^H NMR and ^13^C NMR spectra were recorded on Bruker (^1^H, 400 MHz or 600 MHz) spectrometers with tetramethylsilane as the internal standard. Chemical shifts (*δ*) are reported in parts per million (ppm) relative to the reference solvents used. The following abbreviations were used to report the multiplicity: br, broad; m, multiplet; s, singlet; d, doublet; t, triplet; q, quartette; dd, doublet of doublets; dt, doublet of triplets. High-resolution mass spectrometry results were recorded on the Thermo Q-Exactive time-of-flight LC/MS system.

#### 4.1.1. Synthesis of 2-methyl-3-(4,4,5,5-tetramethyl-1,3,2-dioxaborolan-2-yl)benzonitrile (2)

To a solution of 3-bromo-2-methylbenzonitrile (10.00 g, 51 mmol) in 1,4-dioxane (100 ml), Bis(pinacolato)diboron (15.54 g, 61 mmol), potassium acetate (10.01 g, 102 mmol) and Pd(dppf)Cl_2_ (1.12 g, 1.53 mmol) were added under nitrogen. The reaction was refluxed for 5 h and was cooled down to room temperature. The precipitate formed in the mixture was filtered. 50 ml of water was added to the filtrate and then extracted with ethyl acetate (30 ml × 3). The combined organic layers were washed with brine and dried over Na_2_SO_4_. After filtration and evaporation, the condensation was purified with a silica gel column (petroleum ether/ethyl acetate = 20:1) to yield compound **2** as white solid (11.15 g, 77%). ^1^H NMR (600 MHz, DMSO-*d*_6_) *δ* (ppm) 7.91 (d, *J* = 7.2 Hz, 1H), 7.86 (t, *J* = 6.7 Hz, 1H), 7.38 (q, *J* = 6.9 Hz, 1H), 2.66 (s, 3H), 1.31 (s, 12H). MS (ESI), *m*/*z*: 244.23 [M + H]^+^.

#### 4.1.2. Synthesis of 3-(2-amino-6-chloropyrimidin-4-yl)-2-methylbenzonitrile (3)

4,6-Dichloropyrimidin-2-amine (4.55 g, 27.77 mmol), K_2_CO_3_ (7.68 g, 55.53 mmol) and Pd(PPh_3_)_4_ (1.07 g, 0.93 mmol) were dissolved in DMF (50 ml) under nitrogen. Then, water (5 ml) was added and the mixture was heated to 45 °C. A solution of compound **2** (4.50 g, 18.51 mmol) in DMF was added dropwise to the reaction mixture. The mixture was then heated and stirred at 115 °C for 5 h and was cooled down to room temperature. 50 ml of water was added and the mixture solution was extracted three times with ethyl acetate (30 ml × 3). The combined organic layers were washed with brine, dried over Na_2_SO_4,_ and concentrated. The condensation was purified with a silica gel column (petroleum ether/ethyl acetate = 10:1) to yield compound **3** as white solid (1.68 g, 37%).^1^H NMR (600 MHz, DMSO-*d*_6_) *δ* (ppm) 7.89 (d, *J* = 7.7 Hz, 1H), 7.70 (d, *J* = 7.2 Hz, 1H), 7.50 (t, *J* = 7.7 Hz, 1H), 7.29 (s, 2H), 6.86 (s, 1H), 2.51 (s, 3H). MS (ESI), *m*/*z*: 245.15 [M + H]^+^.

#### 4.1.3. Synthesis of 3-(2-amino-6-((trimethylsilyl)ethynyl)pyrimidin-4-yl)-2-methylbenzonitrile (4)

To a solution of compound **3** (1.00 g, 4.08 mmol) in dry THF (40 ml) were added triethylamine (TEA) (1.24 g, 12.24 mmol) and trimethylsilylacetylene (0.601 g, 6.12 mmol), and the reaction mixture was stirred under a nitrogen atmosphere. After stirring for 5 min, Pd(PPh_3_)_2_Cl_2_ (72 mg, 0.102 mmol) and CuI (39 mg, 0.204 mmol) were added. The reaction mixture was refluxed overnight before being cooled to room temperature. The mixture was evaporated under reduced pressure and dissolved in ethyl acetate (30 ml). The mixture was stirred for 5 min, Suction filtration to remove insoluble matter. Then the organic layer was washed with brine, dried over anhydrous Na_2_SO_4_, and concentrated under reduced pressure. The resulting compound **4** as black solid (1.12 g) was used in the next step without further purification. ^1^H NMR (600 MHz, DMSO-*d*_6_) *δ* (ppm) 7.63 (dd, *J* = 7.7, 1.2 Hz, 1H), 7.45 (dd, *J* = 7.7, 1.2 Hz, 1H), 7.24 (t, *J* = 7.8 Hz, 1H), 6.74 (s, 2H), 6.56 (s, 1H), 2.25 (s, 3H), 0.00 (s, 9H). MS (ESI), *m*/*z*: 307.40 [M + H]^+^.

#### 4.1.4. Synthesis of 3-(2-amino-6-ethynylpyrimidin-4-yl)-2-methylbenzonitrile (5)

Compound **4** (1.12 g) was dissolved in dry THF (15 ml) under a nitrogen atmosphere. And 6 ml of TBAF (1 M in THF) was added to the mixture dropwise under external ice bath cooling, and the reaction was stirred at room temperature for 20 h. The reaction mixture was quenched by pouring into aqueous NH_4_Cl (30 ml) at 0 °C and extracted with ethyl acetate (30 ml × 3). The combined organic layer was washed with water (30 ml), dried on MgSO_4_, evaporated under reduced pressure and purified by column chromatography (petroleum ether/ethyl acetate = 3:1) to obtain pure compound **5** as brown solid (0.575 g, 60% for two steps). ^1^H NMR (600 MHz, DMSO-*d*_6_) *δ* (ppm) 7.89 (dd, *J* = 7.7, 1.4 Hz, 1H), 7.70 (dd, *J* = 7.8, 1.4 Hz, 1H), 7.49 (t, *J* = 7.8 Hz, 1H), 7.00 (s, 2H), 6.86 (s, 1H), 4.52 (s, 1H), 2.51 (s, 3H). MS (ESI), *m*/*z*: 235.41 [M + H]^+^.

#### 4.1.5. General synthetic procedure for compounds 7a, 7d–7g, 7m–7n

To a solution of compound **5** (350 mg, 1.50 mmol) in DMF (3 ml), *t*-BuOH (15 ml) and water (9 ml) were added compound **6** (1.65 mmol), CuSO_4_·5H_2_O (11 mg, 0.045 mmol) and sodium l-ascorbate (13 mg, 0.068 mmol). The reaction was allowed to be stirred for 12 h at 65 °C and then concentrated under reduced pressure to remove the solvent. The concentrate was then purified by column chromatography (DCM/MeOH =50:1 to 25:1) to afford compounds **7a**, **7d**–**7g**, **7m**–**7n**.

3-(2-Amino-6–(1-(quinolin-2-ylmethyl)-1*H*-1,2,3-triazol-4-yl)pyrimidin-4-yl)-2-methylbenzonitrile (**7a**), tawny solid. Yield 78%; m.p. 203–204 °C; ^1^H NMR (600 MHz, DMSO-*d*_6_) *δ* (ppm) 8.78 (s, 1H), 8.43 (d, *J* = 8.5 Hz, 1H), 8.01 (d, *J* = 8.1 Hz, 1H), 7.97 (d, *J* = 8.5 Hz, 1H), 7.90 (d, *J* = 7.7 Hz, 1H), 7.81–7.74 (m, 2H), 7.63 (t, *J* = 7.5 Hz, 1H), 7.52 (t, *J* = 7.7 Hz, 1H), 7.47 (d, *J* = 8.5 Hz, 1H), 7.30 (s, 1H), 6.89 (s, 2H), 6.06 (s, 2H), 2.56 (s, 3H). ^13^C NMR (151 MHz, DMSO-*d*_6_) *δ* (ppm) 166.12, 163.00, 161.69, 157.57, 154.72, 146.37, 145.34, 139.44, 138.47, 136.97, 133.00, 132.66, 129.55, 128.02, 127.38, 126.53, 126.36, 126.31, 125.10, 117.40, 112.62, 104.47, 54.52, 17.67. HRMS (ESI) calcd for C_24_H_19_N_8_ [M + H]^+^: 419.17272, found: 419.17477.

Methyl 6-((4-(2-amino-6-(3-cyano-2-methylphenyl)pyrimidin-4-yl)-1*H*-1,2,3-triazol-1-yl)methyl)picolinate (**7d**), tawny solid. Yield 78%; m. p. 105–106 °C; ^1^H NMR (600 MHz, DMSO-*d*_6_) *δ* (ppm) 8.72 (s, 1H), 8.07–8.02 (m, 2H), 7.89 (dd, *J* = 7.7, 1.2 Hz, 1H), 7.75 (dd, *J* = 7.8, 1.2 Hz, 1H), 7.55–7.50 (m, 2H), 7.27 (s, 1H), 6.89 (s, 2H), 5.92 (s, 2H), 3.88 (s, 3H), 2.55 (s, 3H). ^13^C NMR (151 MHz, DMSO-*d*_6_) *δ* (ppm) 167.16, 165.28, 164.08, 158.60, 155.83, 147.81, 146.45, 140.46, 139.56, 139.50, 134.09, 133.74, 127.43, 126.28, 125.92, 124.80, 118.47, 113.67, 105.48, 54.86, 53.01, 18.72. HRMS (ESI) calcd for C_22_H_19_N_8_O_2_ [M + H]^+:^ 427.16255, found: 427.16251.

Isopropyl 6-((4-(2-amino-6-(3-cyano-2-methylphenyl)pyrimidin-4-yl)-1*H*-1,2,3-triazol-1-yl)methyl)picolinate (**7e**), white solid. Yield 80%; m.p. 128–129 °C; ^1^H NMR (600 MHz, DMSO-*d*_6_) *δ* (ppm) 8.73 (s, 1H), 8.05–7.98 (m, 2H), 7.90 (dd, *J* = 7.8, 1.4 Hz, 1H), 7.75 (dd, *J* = 7.7, 1.4 Hz, 1H), 7.52 (td, *J* = 7.8, 0.7 Hz, 1H), 7.48 (dd, *J* = 7.5, 1.4 Hz, 1H), 7.27 (s, 1H), 6.88 (s, 2H), 5.93 (s, 2H), 5.15 (hept, *J* = 6.3 Hz, 1H), 2.55 (s, 3H), 1.32 (d, *J* = 6.3 Hz, 6H). ^13^C NMR (151 MHz, DMSO-*d*_6_) *δ* (ppm) 166.07, 163.13, 163.01, 157.55, 154.76, 147.18, 145.38, 139.39, 138.47, 138.33, 133.00, 132.66, 126.36, 124.99, 124.77, 123.54, 117.39, 112.59, 104.40, 68.39, 53.75, 20.96, 17.64. HRMS (ESI) calcd for C_24_H_23_N_8_O_2_ [M + H]^+^: 455.19385, found: 455.19458.

6-((4-(2-Amino-6-(3-cyano-2-methylphenyl)pyrimidin-4-yl)-1*H*-1,2,3-triazol-1-yl)methyl)picolinonitrile (**7f**), off white solid. Yield 41%; m.p. 147–148 °C; ^1^H NMR (600 MHz, DMSO-*d*_6_) *δ* (ppm) 8.74 (s, 1H), 8.12 (t, *J* = 7.8 Hz, 1H), 8.03 (d, *J* = 7.7 Hz, 1H), 7.90 (d, *J* = 7.7 Hz, 1H), 7.76 (d, *J* = 7.7 Hz, 1H), 7.70 (d, *J* = 8.0 Hz, 1H), 7.52 (t, *J* = 7.7 Hz, 1H), 7.28 (s, 1H), 6.89 (s, 2H), 5.94 (s, 2H), 2.55 (s, 3H). ^13^C NMR (151 MHz, DMSO-*d*_6_) *δ* (ppm) 167.18, 164.08, 158.57, 157.30, 146.45, 140.45, 139.86, 139.56, 134.09, 133.74, 132.72, 129.02, 127.43, 126.96, 126.17, 118.47, 117.67, 113.67, 105.50, 54.30, 18.72. HRMS (ESI) calcd for C_21_H_16_N_9_ [M + H]^+^: 394.15232, found: 394.15234.

3-(6-(1-((6-(1*H*-imidazol-2-yl)pyridin-2-yl)methyl)-1*H*-1,2,3-triazol-4-yl)-2-aminopyrimidin-4-yl)-2-methylbenzonitrile (**7 g**), grey solid. Yield 6.9%; m.p. 139–140 °C; ^1^H NMR (600 MHz, DMSO-*d*_6_) *δ* (ppm) 12.67 (s, 1H), 8.79 (s, 1H), 7.99 (d, *J* = 7.9 Hz, 1H), 7.92–7.88 (m, 2H), 7.75 (dd, *J* = 7.8, 1.4 Hz, 1H), 7.52 (t, *J* = 7.7 Hz, 1H), 7.28 (s, 2H), 7.20 (d, *J* = 7.6 Hz, 1H), 7.09 (s, 1H), 6.85 (s, 2H), 5.85 (s, 2H), 2.55 (s, 3H). ^13^C NMR (151 MHz, DMSO-*d*_6_) *δ* (ppm) 167.10, 164.07, 158.68, 154.85, 149.37, 146.51, 145.44, 140.45, 139.55, 139.04, 134.09, 133.74, 130.18, 127.43, 125.72, 121.67, 119.49, 119.37, 118.46, 113.67, 105.63, 55.29, 18.72. HRMS (ESI) calcd for C_23_H_19_N_10_ [M + H]^+^: 435.17887, found: 435.17847.

3-(2-Amino-6-(1-(3-methoxybenzyl)-1*H*-1,2,3-triazol-4-yl)pyrimidin-4-yl)-2-methylbenzonitrile (**7 m**), grey solid. Yield 82%; m.p. 163–164 °C; ^1^H NMR (400 MHz, DMSO-*d*_6_) *δ* (ppm) 8.61(s, 1H), 7.84 (d, *J* = 7.7 Hz, 1H), 7.70 (d, *J* = 7.7 Hz, 1H), 7.47 (t, *J* = 7.7 Hz, 1H), 7.27 (t, *J* = 7.9 Hz, 1H), 7.21(s, 1H), 6.96–6.93 (m, 1H), 6.88 (dd, *J* = 7.7, 1.5 Hz, 2H), 6.83 (s, 2H), 5.63 (s, 2H), 3.71 (s, 3H), 2.50 (s, 3H). ^13^C NMR (101 MHz, DMSO-*d*_6_) *δ* (ppm) 167.19, 164.14, 160.02, 158.68, 146.52, 140.52, 139.62, 137.71, 134.15, 133.79, 130.55, 127.48, 125.20, 120.72, 118.53, 114.47, 114.15, 113.74, 105.55, 55.68, 53.55, 18.78. HRMS (ESI) calcd for C_22_H_20_N_7_O [M + H]^+^: 398.17238, found: 398.17227.

3-(2-Amino-6-(1-(4-methoxybenzyl)-1*H*-1,2,3-triazol-4-yl)pyrimidin-4-yl)-2-methylbenzonitrile (**7n**), white solid. Yield 78%; m.p. 149–150 °C; ^1^H NMR (400 MHz, DMSO-*d*_6_) *δ* (ppm) 8.54 (s, 1H), 7.84 (d, *J* = 7.7 Hz, 1H), 7.70 (dd, *J* = 7.7, 1.0 Hz, 1H), 7.46 (t, *J* = 7.7 Hz, 1H), 7.36–7.30 (m, 2H), 7.21 (s, 1H), 6.91 (m, 2H), 6.83 (s, 2H), 5.58 (s, 2H), 3.70 (s, 3H), 2.50 (s, 3H). ^13 ^C NMR (101 MHz, DMSO-*d*_6_) *δ* (ppm) 167.17, 164.14, 159.78, 158.71, 146.49, 140.52, 139.61, 134.14, 133.77, 130.35, 128.16, 127.46, 124.82, 118.53, 114.74, 113.74, 105.51, 55.69, 53.19, 18.77. HRMS (ESI) calcd for C_22_H_20_N_7_O [M + H]^+^: 398.17238, found: 398.17218.

#### 4.1.6. Synthesis of 3-(2-amino-6-(1-((8-aminoquinolin-2-yl)methyl)-1H-1,2,3-triazol-4-yl)pyrimidin-4-yl)-2-methylbenzonitrile (7c)

3-(2-Amino-6-(1-((8-nitroquinolin-2-yl)methyl)-1*H*-1,2,3-triazol-4-yl)pyrimidin-4-yl)-2-methylbenzonitrile (**7b**) was prepared in the same way as compound **7a** from compound **5** and 2-(azidomethyl)-8-nitroquinoline (**6b**), yellow solid. Yield 43%; MS (ESI), *m*/*z*: 464.31 [M + H]^+^.

Compound **7b** (100 mg, 0.216 mmol) was dissolved in ethanol (15 ml) at room temperature, then SnCl_2_ (205 mg, 1.08 mmol) was added and the mixture was stirred at 70 °C for 2 h. After the completion of the reaction, the mixture was poured into water (50 ml) and concentrated under reduced pressure. The crude residue was purified by reverse silica gel column chromatography (MeOH/H_2_O = 0% to 50%) and recrystallized from methanol to afford the compound **7c**, off white solid. Yield 9.8%; m.p. 268–269 °C; ^1^H NMR (600 MHz, DMSO-*d*_6_) *δ* (ppm) 8.79 (s, 1H), 8.23 (d, *J* = 8.5 Hz, 1H), 7.89 (dd, *J* = 7.7, 1.2 Hz, 1H), 7.76 (dd, *J* = 7.8, 1.1 Hz, 1H), 7.52 (t, *J* = 7.7 Hz, 1H), 7.42 (d, *J* = 8.5 Hz, 1H), 7.32–7.27 (m, 2H), 7.07 (dd, *J* = 8.1, 1.1 Hz, 1H), 6.90–6.83 (m, 3H), 5.99 (s, 2H), 5.81 (s, 2H), 2.55 (s, 3H). ^13^C NMR (151 MHz, DMSO-*d*_6_) *δ* (ppm) 167.11, 164.10, 158.71, 152.06, 146.38, 145.21, 140.46, 139.57, 137.82, 136.84, 134.11, 133.73, 128.43, 128.17, 127.42, 126.03, 120.37, 118.48, 114.06, 113.67, 109.65, 105.55, 55.60, 18.73. HRMS (ESI) calcd for C_24_H_20_N_9_ [M + H]^+^: 434.18362, found: 434.18289.

#### 4.1.7. Synthesis of 6-((4-(2-amino-6–(3-cyano-2-methylphenyl)pyrimidin-4-yl)-1H-1,2,3-triazol-1-yl)methyl)picolinic acid (7h)

Compound **7d** (128 mg, 0.30 mmol) were dissolved in a mixed solution of *tert* butanol (10 ml) and water (5 ml). Then LiOH (18.5 mg, 0.441 mmol) was added and the reaction mixture was continue stirred for 8 h. After reaction, aqueous HCl (1 M) was added to adjust the pH to 5–6. Next, the mixture solution was extracted with ethyl acetate (20 ml × 3). The combined organic layer was washed with brine (30 ml), dried over Na_2_SO_4_, then evaporation. The condensation was purified with a silica gel column (DCM/MeOH= 20: 1) to yield compound **7h**, white solid. Yield 65%; m.p. 176–177 °C; ^1^H NMR (600 MHz, DMSO-*d*_6_) *δ* (ppm) 13.32 (s, 1H), 8.72 (s, 1H), 8.01 (d, *J* = 6.3 Hz, 2H), 7.89 (dd, *J* = 7.8, 1.3 Hz, 1H), 7.76 (dd, *J* = 7.8, 1.3 Hz, 1H), 7.52 (t, *J* = 7.7 Hz, 2H), 7.27 (s, 1H), 6.89 (s, 2H), 5.91 (s, 2H), 2.55 (s, 3H). ^13^C NMR (151 MHz, DMSO-*d*_6_) *δ* (ppm) 167.15, 166.31, 164.09, 158.63, 155.48, 146.44, 140.46, 139.56, 139.30, 134.09, 133.73, 127.43, 125.89, 125.84, 124.59, 118.47, 113.67, 105.49, 55.38, 18.72. HRMS (ESI) calcd for C_21_H_15_N_8_O_2_ [M-H] ^-^: 411.13235, found: 411.13293.

#### 4.1.8. General synthetic procedure for compounds 7i–7l

A solution of compound **7d** (50 mg, 0.12 mmol) in MeOH (3 ml) and THF (3 ml) was cooled to −10 °C and ammonia or its derivatives (2.4 mmol) was added under nitrogen. The reaction mixture was stirred at 45 °C for 4–16 h. The reaction progress was monitored by TLC. After completion of the reaction, the reaction mixture was diluted with water (15 ml) and extracted with ethyl acetate (10 ml × 3). The combined organic phase was washed with brine (20 ml), dried over anhydrous Na_2_SO_4_, filtered, and concentrated under reduced pressure. The reaction residue thus obtained was purified by flash column chromatography (DCM/MeOH = 50:1 to 20:1) to afford the target compounds **7i-7l**.

6-((4-(2-Amino-6-(3-cyano-2-methylphenyl)pyrimidin-4-yl)-1*H*-1,2,3-triazol-1-yl)methyl)picolinamide (**7i**), white solid. Yield 44%; m.p. 151–152 °C; ^1^H NMR (600 MHz, DMSO-*d*_6_) *δ* (ppm) 8.83 (s, 1H), 8.03 (t, *J* = 7.7 Hz, 1H), 7.99 (dd, *J* = 7.7, 1.2 Hz, 1H), 7.94 (s, 1H), 7.89 (dd, *J* = 7.7, 1.2 Hz, 1H), 7.76 (dd, *J* = 7.8, 1.1 Hz, 1H), 7.73 (s, 1H), 7.52 (t, *J* = 7.7 Hz, 2H), 7.27 (s, 1H), 6.86 (s, 2H), 5.89 (s, 2H), 2.55 (s, 3H). ^13 ^C NMR (151 MHz, DMSO-*d*_6_) *δ* (ppm) 167.11, 165.89, 164.08, 158.67, 154.30, 150.49, 146.46, 140.45, 139.57, 139.56, 134.10, 133.74, 127.43, 126.01, 125.31, 121.71, 118.47, 113.67, 105.61, 54.83, 18.72. HRMS (ESI) calcd for C_21_H_18_N_9_O [M + H]^+^: 412.16288, found: 412.16293.

6-((4-(2-Amino-6-(3-cyano-2-methylphenyl)pyrimidin-4-yl)-1*H*-1,2,3-triazol-1-yl)methyl)-N-(2-hydroxyethyl)picolinamide (**7j**), white solid. Yield 64%; m.p. 208–209 °C; ^1^H NMR (600 MHz, DMSO-*d*_6_) *δ* (ppm) 8.80 (s, 1H), 8.55 (t, *J* = 5.9 Hz, 1H), 8.05–7.99 (m, 2H), 7.90 (dd, *J* = 7.7, 1.3 Hz, 1H), 7.76 (dd, *J* = 7.7, 1.3 Hz, 1H), 7.52 (t, *J* = 7.7 Hz, 1H), 7.45 (dd, *J* = 7.4, 1.4 Hz, 1H), 7.28 (s, 1H), 6.87 (s, 2H), 5.91 (s, 2H), 4.80 (t, *J* = 5.4 Hz, 1H), 3.52 (q, *J* = 5.8 Hz, 2H), 3.39 (q, *J* = 6.0 Hz, 2H), 2.55 (s, 3H). ^13^C NMR (151 MHz, DMSO-*d*_6_) *δ* (ppm) 167.13, 164.07, 163.76, 158.62, 154.42, 150.18, 146.55, 140.45, 139.74, 139.56, 134.09, 133.73, 127.42, 125.93, 125.21, 121.70, 118.46, 113.67, 105.59, 60.11, 54.93, 42.08, 18.72. HRMS (ESI) calcd for C_23_H_22_N_9_O_2_ [M + H]^+^: 456.18910, found: 456.18927.

3-(2-Amino-6-(1-((6–(3-hydroxyazetidine-1-carbonyl)pyridin-2-yl)methyl)-1*H*-1,2,3-triazol-4-yl)pyrimidin-4-yl)-2-methylbenzonitrile (**7k**), grey solid. Yield 36%; m.p. 243–244 °C; ^1^H NMR (600 MHz, DMSO-*d*_6_) *δ* (ppm) 8.71 (s, 1H), 8.01 (t, *J* = 7.8 Hz, 1H), 7.90 (ddd, *J* = 7.8, 4.8, 1.2 Hz, 2H), 7.76 (dd, *J* = 7.8, 1.4 Hz, 1H), 7.57 (dd, *J* = 7.8, 1.0 Hz, 1H), 7.52 (td, *J* = 7.7, 0.7 Hz, 1H), 7.27 (s, 1H), 6.86 (s, 2H), 5.91 (s, 2H), 5.64 (d, *J* = 6.3 Hz, 1H), 4.43 (ddd, *J* = 10.7, 6.7, 1.5 Hz, 1H), 4.41 − 4.36 (m, 1H), 4.20 (ddd, *J* = 10.9, 6.8, 1.7 Hz, 1H), 4.00 (ddd, *J* = 10.4, 4.1, 1.4 Hz, 1H), 3.73 (ddd, *J* = 10.7, 4.3, 1.3 Hz, 1H), 2.55 (s, 3H). ^13 ^C NMR (151 MHz, DMSO-*d*_6_) *δ* (ppm) 167.11, 164.20, 164.08, 158.67, 154.14, 151.77, 146.38, 140.49, 139.58, 139.06, 134.10, 133.72, 127.41, 126.18, 124.56, 122.99, 118.48, 113.66, 105.57, 64.45, 61.32, 59.07, 54.49, 18.71. HRMS (ESI) calcd for C_24_H_22_N_9_O_2_ [M + H]^+^: 468.18910, found: 468.18942.

3-(2-Amino-6-(1-((6–(2-(hydroxymethyl)pyrrolidine-1-carbonyl)pyridin-2-yl)methyl)-1*H*-1,2,3-triazol-4-yl)pyrimidin-4-yl)-2-methylbenzonitrile (**7l**), white solid. Yield 52%; m.p. 98–99 °C; ^1^H NMR (600 MHz, DMSO-*d*_6_) *δ* (ppm) 8.72 (s, 1H), 7.98 (t, *J* = 7.8 Hz, 1H), 7.89 (d, *J* = 7.7 Hz, 1H), 7.75 (d, *J* = 6.9 Hz, 1H), 7.71 (d, *J* = 7.6 Hz, 1H), 7.52 (t, *J* = 7.0 Hz, 2H), 7.28 (s, 1H), 6.88 (s, 2H), 5.91 (s, 2H), 3.44 (t, *J* = 7.0 Hz, 2H), 3.32 (t, *J* = 6.8 Hz, 2H), 2.55 (s, 3H), 1.75 (p, *J* = 6.8 Hz, 2H), 1.66 (p, *J* = 6.8 Hz, 2H). ^13^C NMR (151 MHz, DMSO-*d*_6_) *δ* (ppm) 167.11, 165.13, 164.09, 158.67, 154.22, 153.77, 146.31, 140.46, 139.52, 138.88, 134.05, 133.73, 127.43, 126.38, 123.69, 123.30, 118.46, 113.67, 105.41, 54.53, 48.78, 46.95, 26.46, 23.86, 18.69. HRMS (ESI) calcd for C_25_H_24_N_9_O [M + H]^+^: 466.20983, found: 466.20834.

#### 4.1.9. Synthesis of 2-(6-((4-(2-amino-6-(5-methylfuran-2-yl)pyrimidin-4-yl)-1H-1,2,3-triazol-1-yl)methyl)pyridin-2-yl)propan-2-ol (17)

Compound **17** was prepared in the same way as **7a** from 4-ethynyl-6-(5-methylfuran-2-yl)pyrimidin-2-amine (**14**) with 2-(6-(azidomethyl)pyridin-2-yl)propan-2-ol (**16**).

2-(6-((4-(2-Amino-6-(5-methylfuran-2-yl)pyrimidin-4-yl)-1*H*-1,2,3-triazol-1-yl)methyl)pyridin-2-yl)propan-2-ol (**17**), pale yellow solid. Yield 38%; m.p. 188–189 °C; ^1^H NMR (600 MHz, DMSO-*d*_6_) *δ* 8.61 (s, 1H), 7.80 (t, *J* = 7.8 Hz, 1H), 7.61 (d, *J* = 7.6 Hz, 1H), 7.46 (s, 1H), 7.14 (d, *J* = 3.3 Hz, 1H), 7.10 (d, *J* = 7.5 Hz, 1H), 6.70 (s, 2H), 6.33 (dd, *J* = 3.3, 1.0 Hz, 1H), 5.80 (s, 2H), 5.21 (s, 1H), 2.40 (s, 3H), 1.38 (s, 6H). ^13^C NMR (151 MHz, DMSO) *δ* 168.59, 164.25, 158.60, 156.78, 155.20, 153.56, 150.73, 146.48, 138.22, 125.52, 119.87, 118.43, 113.31, 109.37, 99.17, 72.73, 55.08, 31.03, 14.05. HRMS (ESI) calcd for C_20_H_22_N_7_O_2_ [M + H]^+^: 392.18295, found: 392.18250.

#### 4.1.10. Synthesis of 2-(6-((4-(2-amino-6-(3-(fluoromethyl)phenyl)pyrimidin-4-yl)-1H-1,2,3-triazol-1-yl)methyl)pyridin-2-yl)propan-2-ol (22)

To a solution of compound **21** (384 mg, 1.00 mmol) and compound **16** (288 mg, 1.50 mmol) in *t*-BuOH (4 ml) and water (4 ml) were added CuSO_4_·5H_2_O (25 mg, 0.10 mmol), sodium l-ascorbate (40 mg, 0.20 mmol) and TBAF (1 M in THF) (2.00 ml, 2.00 mmol). The reaction was allowed to be stirred for 12 h at 60 °C and then concentrated under reduced pressure to remove the solvent. The concentrate was then purified by column chromatography (DCM/MeOH = 50:1) to afford compound **22**, white solid. Yield 4.5%; m.p. 76–77 °C; ^1^H NMR (600 MHz, DMSO-*d*_6_) *δ* 8.68 (s, 1H), 8.21 (s, 1H), 8.17–8.13 (m, 1H), 7.80 (t, *J* = 7.8 Hz, 1H), 7.74 (s, 1H), 7.61 (d, *J* = 8.0 Hz, 1H), 7.58 (m, 2H), 7.12 (d, *J* = 7.6 Hz, 1H), 6.81 (s, 2H), 5.82 (s, 2H), 5.54 (d, *J* = 47.6 Hz, 2H), 5.22 (s, 1H), 1.38 (s, 6H). ^13^C NMR (151 MHz, DMSO) *δ* 167.53, 163.62, 163.41, 158.05, 152.49, 145.44, 137.16, 136.20 (d, *J* = 16.7 Hz), 130.87 (d, *J* = 9.5 Hz), 129.17 (d, *J* = 5.8 Hz), 128.14 (d, *J* = 11.8 Hz), 126.43 (d, *J* = 2.8 Hz), 125.42 (d, *J* = 5.9 Hz), 124.70, 118.79, 117.37, 100.42, 83.47 (d, *J* = 162.3 Hz), 71.66, 54.03, 29.96. HRMS (ESI) calcd for C_22_H_23_FN_7_O [M + H]^+^: 420.19426, found: 420.19394.

### 4.2. Pharmacology

#### 4.2.1. Cyclic AMP functional assay

CHO-K1/ADORA2A/Gα15 cells expressing human A_2A_ AR and CHO-K1/ADORA2b/Gα15 cells expressing human A_2B_ AR were both constructed by Genscript Biotech Corporation. These two types of cells were cultured in an F12K complete medium (Gibco) at 37 °C under 5% CO_2_. The 4X test compound (ZM241385, AB928, and target compounds) stock solutions and 4X NECA stock solution were prepared with assay buffer (Hank’s buffered saline solution) for use. The test stock solutions were diluted with different gradients for use. The procedure was exemplified by the cAMP functional assay on the A_2A_ receptor: 10,000 cells/well of CHO-K1/ADORA2A/Gα15 were seeded in a 384-well plate in 20 μl of assay buffer. Test compound solutions with different concentrations (2.5 μl) and NECA stock solution (2.5 μl) were added to indicated well of the 384-well plate prepared above and incubated at 37 °C for 30 min. Then, 10 μl of detection reagent (cAMP-d2 and anti-cAMP-Eu3+) was added into each well of the plate and incubated at room temperature for 1 h. The plate was transferred into the PHERA Star for HTRF detection and the data (ratio 665/620) was collected at the wavelength of 665 nm and 620 nm. The inhibition was calculated according to the formula: inhibition (%)=(*R_s_*–*R*_0_)/(*R*_1_–*R*_0_)×100%, where *R_s_* is the ratio 665/620 value in the presence of target compounds, *R*_0_ is the ratio 665/620 value during the blank assay and *R*_1_ is the ratio 665/620 value in the presence of ZM241385 at a concentration of 1 μM.

#### 4.2.2. A2a AR binding assay

The target compounds were tested to evaluate their affinity for the A_2A_ AR on HEK-293 cell membranes expressing human A_2A_ AR. [^3^H]-ZM241385 was used as a radioligand. The assay buffer (50 mM Tris-HCl, pH 7.4; 10 mM MgCl_2_; 1 mM EDTA; 1 μg/ml adenosine deaminase), wash buffer (50 mM Tris-HCl, pH 7.4; 154 mM NaCl), and 0.5% PEI solution were prepared and stored at 4 °C for use. The affinities were tested following the procedure reported by Hu et al^4^.

#### 4.2.3 T-cell activation assay

Cryopreserved PBMCs (Allcells) were thawed and recovered in RPMI1640 medium overnight before being used. And compounds to be tested were diluted with DMSO to 100 folds of working concentrations. Then PBMCs were added to 96-well U bottom plate (2.5 × 10^5^ cells per well). To the cells, 2 μl of NECA and 2 μl of each diluted compound (a final concentration of DMSO is 2%) were added. Then, cells were incubated at 37 °C under 5% CO_2_ for 1 h. Add 100 μl anti-CD3 (2 μg/ml) and anti-CD28 antibody (2 μg/ml) to each well and cells were cultured for 48 h. After 48 h, supernatants were collected and IL-2 concentration was detected by ELISA.

### 4.3. Molecular docking

The crystal structure of hA_2A_ AR in complex with antagonist ZM241385 (PDB ID: 5IU4) was retrieved from the Protein Data Bank. The primary sequence of hA_2B_ AR was obtained from the NCBI/UNIPROT online database (www.ncbi.nlm.nih.gov/ protein/P29275.1). Based on the primary sequence, the homology model of hA_2B_ AR was built using the Swiss Model program from a hA_2A_ AR crystal structure as the template (PDB ID: 6PS7)^40^ and has been checked using the Ramachandran plot application within Discovery Studio 2017 R2^41^. The molecular docking was performed under the C-DOCKER protocol of Discovery Studio. First, the 3D structure of **7i** was generated and the hydrogenation, dehydration, and CHARMm force field of the protein were executed. Then, the protein was remodelled by removing the antagonist and the 3D molecule of **7i** was placed at the active site for molecular docking. Next, the 2D and 3D predicted binding models of compound **7i** in hA_2A_ AR and hA_2B_ AR were generated.

### 4.4. Metabolic stability in human and rat liver microsomes

Metabolic stabilities of compounds **7f**, **7i**, and AB928 in liver microsomes were studied with pooled human (male) liver microsomes solution (20 mg/ml) and male Sprague–Dawley rats pooled liver microsomes solution (20 mg/ml) purchased from Research Institute for Liver Diseases (Shanghai) Co. Ltd. Both liver microsomes solutions (140 μl) were diluted by 3948 μl of water and 280 μl of phosphate buffered saline. The assay procedure: 640 μl of diluted liver microsomes solution was added to a solution of compound working solution (16 μl, 25 μM in DMSO) and the mixed solution was incubated at 37 °C for 5 min. Then 160 μl of NADPH generating system was added and take out 100 μl from the mixed solution was added 200 μl of acetonitrile solution to stop the reaction at the set incubation time (0 min, 10 min, 20 min, 30 min, 40 min, 50 min, and 60 min). All stopped reaction solutions were vortex mixed (3 min) and centrifuged (10,000 rpm, 4 °C for 10 min). Then 50 μl of the supernatant was analysed by UPLC-MS/MS system (details of the analytical method see Supporting information).

### 4.5. Pharmacokinetics assay

Animal experiments were performed according to the institutional ethical guidelines on animal care and approved by the Institute Animal Care and Use Committee at Binzhou Medical University.

The absolute bioavailabilities of synthetic compounds (**7f**, **7i)** were studied in female Bal b/c mice (Jinan Pengyue Experimental Animal Breeding Co., Ltd.). The mice were divided into four groups each having six mice and were collected at crossover time points, with three mice at each point. Pharmacokinetics (PK) was evaluated after a single dose of 30 mg/kg oral gavage (Oral) or 10 mg/kg intravenous (i.v.) administration. After oral administration and i.v. injection, blood samples were obtained from the suborbital veniplex at pre, 15 min, 30 min, 1 h, 2 h, 4 h, 6 h, 8 h, 12 h, 24 h (Oral) and pre, 5 min, 15 min, 30 min, 1 h, 2 h, 4 h, 6 h, 8 h, 12 h, 24 h (i.v.). Approximately 200 μl of blood was collected at each time point. All blood samples were put into plastic microcentrifuge tubes containing Heparin-Na as an anticoagulant. Microcentrifuge tubes with blood samples and anticoagulant were inverted several times for proper mixing of the tube contents to centrifugation for plasma. Plasma samples will be centrifuged at 12,000 rpm for 8 min at 4 °C to obtain the supernatant. The serum sample (25 μl) was treated with acetonitrile (100 μl), after which the mixture was vortex-mixed for 8 min and centrifuged at 10,000 rpm for 10 min at 4 °C. The supernatant layer was collected and then 50 μl of supernatant was injected for the UPLC-MS/MS analysis (details of the analytical method see Supporting information).

## Supplementary Material

Supplemental Material
