# Supplementary material for: Design, synthesis, and biological evaluation of triazole-pyrimidine-methylbenzonitrile derivatives as dual A2A/A2B adenosine receptor antagonists
Source: J Enzyme Inhib Med Chem. 2022 May 26;37(1):1514–26. doi: 10.1080/14756366.2022.2077731 (PMC9154793; doi:10.1080/14756366.2022.2077731)
Supplement: Supplemental Material [file IENZ_A_2077731_SM6369.pdf]

Supporting Information

Design, synthesis, and biological evaluation of triazole-pyrimidine-methylbenzonitrile derivatives as dual A_{2A}/A_{2B} adenosine receptor antagonists

Zhi Li[a], Lijuan Kou[a], Xinzhen Fu[a], Zeping Xie[a], Maolei Xu[a], Lin Guo[a], Tiantian Lin[b], Shizhou Gong[b], Shumin Zhang[a] *, Ming Liu[a] *

[a] *School of Pharmacy, Binzhou Medical University, Yantai 264003, China*
[b] *Luye Pharma Group, Yantai 264670, China*

* Corresponding author.
E-mail addresses: shumin_zhang@outlook.com (S. Zhang), ytliuming@163.com (M. Liu)

Contents

1. General Information S2
2. Synthesis of intermediates 6, 12-16, 19-21 .... S2
3. ^{1}H NMR spectra of intermediates S7
4. NMR spectra of target compounds 7, 17, 22 .... S14
5. Copies of HRMS S29
6. The Ramachandran plot for the homology model of hA_{2B} AR S37
7. Binding modes of compound 7i with hA_{2A} AR or hA_{2B} AR S38
8. Metabolic stability in human and rat liver microsomes S40
9. Analytical method of pharmacokinetics assay S42

1. General Information

All chemicals were purchased from commercial suppliers and used without further purification. Air or moisture sensitive reactions were performed under positive pressure of nitrogen with oven-dried glassware. Reactions were monitored by thin layer chromatography (TLC), and spots were visualized with iodine vapor or by irradiation with UV light. Flash column chromatography was performed using the Qingdao Haiyang flash silica gel (200-300 mesh). All yields were reported as isolated yields. ^{1}H NMR and ^{13}C NMR spectra were recorded on the Bruker (^{1}H, 400 or 600MHz) spectrometer with tetramethylsilane as the internal standard. Chemical shifts (δ) are reported in parts per million (ppm) relative to the reference solvents used. The following abbreviations were used to report the multiplicities of the peaks: br, broad; m, multiplet; s, singlet; d, doublet; t, triplet; q, quartet; dd, doublet of doublets; dt, doublet of triplets. High resolution mass spectrometry results were recorded on Thermo Q-Exactive time-of-flight LC/MS system.

2. Synthesis of intermediates 6, 12-16, 19-21

2.1 Synthesis of intermediates ***6a-6b***

A solution of compound **8** (4.50 mmol) in dry THF was cooled down to -10 °C under nitrogen. Trimethylsilyl azide (TMSiA) (1.04 g, 9.02 mmol) and N,N-diisopropyl ethylamine (DIPEA) (1.16 g, 8.98 mmol) were added to the solution slowly and then stirred at room temperature for another 24 h. The mixture was filtered, and concentrated under reduced pressure to afford corresponding crude product, which was purified by column chromatography (petroleum ether/ethyl acetate =10:1) to provide compound **6a-6b**.

2-(Azidomethyl) quinoline (**6a**), yellow oil. Yield 62%; ^{1}H NMR (400 MHz, $CDCl_3$) δ (ppm) 8.21 (d, J = 8.4 Hz, 1H), 8.10 (d, J = 8.6 Hz, 1H), 7.83 (dd, J = 8.2, 1.3 Hz, 1H), 7.74 (ddd, J = 8.4, 6.9, 1.5 Hz, 1H), 7.56 (ddd, J = 8.1, 6.9, 1.2 Hz, 1H), 7.48 (d, J = 8.5 Hz, 1H), 4.69 (s, 2H).

2-(Azidomethyl)-8-nitroquinoline (**6b**), black solid. Yield 98%; MS (ESI) m/z:

252.33 $[M+Na]^+$.

2.2 Synthesis of compounds ***6d-6g, 6m-6n***

Compound **9** (7.45 mmol) was dissolved in dry THF (15 mL) under nitrogen. The mixture was then cooled down to -5 °C, diphenyl azidophosphate (DPPA) (2.46 g, 8.95 mmol) and 1,8-Diazabicyclo[5.4.0]undecane-7-ene (DBU) (1.36 g,8.95 mmol) were added to the mixture slowly and then stirred at room temperature for another 12 h. The mixture solution was extracted with EtOAc (30 mL) and water (30 ml). The combined organic phase was washed with brine (30 mL), dried with anhydrous Na_2SO_4, filtered, and concentrated under reduced pressure to afford corresponding crude product, which was purified by column chromatography (petroleum ether/ethyl acetate =10:1) to afford the compound **6d**-**6g**, **6m**-**6n**.

Methyl-6-(azidomethyl)picolinate (**6d**), yellow oil. Yield 80%; ^{1}H NMR (600 MHz, DMSO-d_6) δ (ppm) 8.04 (dt, J = 15.3, 7.5 Hz, 2H), 7.70 (d, J = 7.5 Hz, 1H), 4.64 (s, 2H), 3.90 (s, 3H).

Isopropyl 6-(azidomethyl)picolinate (**6e**), yellow oil. Yield 82%; ^{1}H NMR (600 MHz, DMSO-d_6) δ (ppm) 8.04 (t, J = 7.7 Hz, 1H), 8.00 (dd, J = 7.7, 1.0 Hz, 1H), 7.68 (dd, J = 7.6, 0.8 Hz, 1H), 5.17 (hept, J = 6.3 Hz, 1H), 4.63 (s, 2H), 1.34 (d, J = 6.3 Hz, 6H).

6-(Azidomethyl)picolinonitrile (**6f**), yellow oil. Yield 93%; ^{1}H NMR (600 MHz, DMSO-d_6) δ (ppm) 8.12 (t, J = 7.8 Hz, 1H), 8.03 (d, J = 7.6 Hz, 1H), 7.79 (d, J = 7.9 Hz, 1H), 4.66 (s, 2H).

2-(Azidomethyl)-6-(1H-imidazol-2-yl) pyridine (**6g**), yellow oil. Yield 93%; ^{1}H NMR (600 MHz, DMSO-d_6) δ (ppm) 12.57 (s, 1H), 7.99 (d, J = 7.8 Hz, 1H), 7.92 (t, J = 7.7 Hz, 1H), 7.41 (d, J = 7.5 Hz, 1H), 7.16 (d, J = 69.2 Hz, 2H), 4.60 (s, 2H).

1-(Azidomethyl)-3-methoxybenzene (**6m**), yellow oil. Yield 91%; ^{1}H NMR (400 MHz, DMSO-d_6) δ (ppm) 7.32 – 7.24 (m, 1H), 6.94 – 6.86 (m, 3H), 4.37 (s, 2H), 3.73 (s, 3H).

1-(Azidomethyl)-4-methoxybenzene (**6n**), yellow oil. Yield 89%; ^{1}H NMR (400 MHz, DMSO-d_6) δ (ppm) 7.30 – 7.22 (m, 2H), 6.97 – 6.88 (m, 2H), 4.31 (s, 2H), 3.72 (s, 3H).

2.3 Synthesis of 4-chloro-6-(5-methylfuran-2-yl)pyrimidin-2-amine ***12***

4,6-Dichloropyrimidin-2-amine (0.512 g, 3.12 mmol), K_2CO_3 (0.981 g, 7.20 mmol) and $Pd(PPh_3)_4$ (0.139 g, 0.12 mmol) were dissolved in DMF (15 mL) under nitrogen. The mixture was then heated to 45 °C. A solution of compound **11** (0.500 g, 2.40 mmol) in DMF was added dropwise to the reaction mixture. The mixture was heated and stirred at 115 °C for 5 h. 10 mL of water was added and the mixture solution was extracted with EtOAc (10 mL×3). The combined organic layers were washed with brine, dried over Na_2SO_4 and concentrated. The condensation was purified with a silica gel column (petroleum ether/ethyl acetate = 10:1) to yield compound **12** as white solid. Yield 35%; ^{1}H NMR (400 MHz, DMSO-d_6) δ (ppm) 7.14 (d, J = 3.3 Hz, 1H), 7.10 (s, 2H), 6.84(s, 1H), 6.27 (d, J = 1.0 Hz, 1H), 2.32(s, 3H).

2.4 Synthesis of 4-ethynyl-6-(5-methylfuran-2-yl)pyrimidin-2-amine ***14***

To a solution of compound **12** (0.170 g, 0.81 mmol) in dry THF (10 ml) were added triethylamine (TEA) (0.246g, 2.43 mmol) and trimethylsilylacetylene (TMSA) (0.120 g, 1.22 mmol), and the reaction mixture was stirred under a nitrogen atmosphere. After stirring for 5 min, $Pd(PPh_3)_2Cl_2$ (14 mg, 0.02 mmol) and CuI (8 mg, 0.04 mmol) were added. The reaction mixture was refluxed overnight before being cooled to room temperature. The mixture was evaporated under reduced pressure and dissolved in EtOAc (10 mL). The mixture was stirred for 5 min, Suction filtration to remove insoluble matter. Then the organic layer was washed with brine, dried over anhydrous Na_2SO_4, and concentrated under reduced pressure. The resulting compound **13** as black solid (0.200 g) was used in the next step without further purification. MS (ESI) *m/z*: 272.14 $[M+H]^+$.

Compound **13** (0.200 g) was dissolved in dry THF (15 mL) under a nitrogen atmosphere. And 3 mL of tetrabutylammonium fluoride (TBAF) (1M in THF) was added to the mixture dropwise under external ice bath cooling, and the reaction was stirred at room temperature for 10 h. The reaction mixture was quenched by pouring into aqueous NH_4Cl (10 mL) at 0 °C and extracted with EtOAc (10 mL×3). The combined organic layer was dried with Na_2SO_4, evaporated under reduced pressure and

purified by column chromatography (petroleum ether/ethyl acetate =1:1) to obtain pure compound **14** as brown solid (0.105 g, 65% for two steps). MS (ESI) *m/z*: 200.12 $[M+H]^+$.

2.5 Synthesis of 2-(6-(azidomethyl)pyridin-2-yl)propan-2-ol ***16***

To a solution of compound **9a** (1.00 g，5.98 mmol) in dry THF (15 mL) under N_2 was added methylmagnesium bromide (1 M in THF, 25 mL, 25 mmol) at -10 °C. The mixture was allowed to warm to room temperature and stirred for 12 h. The reaction was then quenched with NH_4Cl aq (30 mL) and extracted with EtOAc (30 mL×3). The combined organic extracts were washed with saturated aqueous NaCl (30 mL), dried with Na_2SO_4, filtered and concentrated in vacuo to give the compound **15** (0.96 g) as a pale yellow oil which was used in the next step without further purification. MS (ESI) *m/z*: 168.10 $[M+H]^+$. Compound **16** was then prepared in the same way as **6d**.

2-(6-(Azidomethyl)pyridin-2-yl)propan-2-ol (**16**), pale yellow oil. Yield 80%; ^1H NMR (400 MHz, DMSO-d_6) δ (ppm) 7.76 (t, J = 7.7 Hz, 1H), 7.58 (d, J = 9.4 Hz, 1H), 7.19 (d, J = 8.8 Hz, 1H), 5.19 (s, 1H), 4.39 (s, 2H), 1.41 (s, 6H).

2.6 Synthesis of 2-(3-(fluoromethyl)phenyl)-4,4,5,5-tetramethyl-1,3,2-dioxaborolane ***19***

To a solution of compound **18** (2.34 g, 10 mmol) and TEA (3.03 g, 30 mmol) in DCM (30 mL) was added MsCl (1.72 g, 15 mmol) dropwise at 0 °C. And the reaction mixture was stirred at 0 °C for 1 h before 80 mL of DCM was added. Then the mixture was washed with water (30 mL×2) and evaporated under reduced pressure. After that, the concentrate was dissolved in 100 mL of TBAF (1M in THF) and was stirred at 50 °C overnight. Then 150 mL of EtOAc was added and the mixture solution was washed with water (50 mL×5). The organic layer was dried with Na_2SO_4, evaporated under reduced pressure and purified by column chromatography (petroleum ether/ethyl acetate =5:1) to obtain pure compound **19** as yellow solid. Yield 76%; MS (ESI) *m/z*: 237.19 $[M+H]^+$.

2.7 Synthesis of 4-chloro-6-((triisopropylsilyl)ethynyl)pyrimidin-2-amine ***20***

To a solution of 4,6-Dichloropyrimidin-2-amine (16.40 g, 0.100 mol) in dry THF (300 mL) were added ethynyltriisopropylsilane (36.50 g, 0.200 mol), $Pd(PPh_3)_2Cl_2$ (7.00 g, 0.010 mol), CuI (1.90 g, 0.010 mol), and TEA (30.30 g， 0.300 mol). Then the reaction mixture was stirred at 80 °C under a nitrogen atmosphere overnight. 500 mL of EtOAc was added and the mixture solution was washed with water (200 mL×3). The organic layer was dried with Na_2SO_4, evaporated under reduced pressure and purified by column chromatography (petroleum ether/ethyl acetate =3:1) to obtain pure compound **20** as pale yellow solid. Yield 64%; MS (ESI) *m/z*: 310.17 $[M+H]^+$.

*2.8 Synthesis of 4-(3-(fluoromethyl)phenyl)-6-((triisopropylsilyl)ethynyl)pyrimidin-2-amine **21***

Compound **19** (1.77 g, 7.5 mmol), compound **20** (1.55 g, 5 mmol), K_2CO_3 (2.07 g, 15 mmol) and $Pd(dppf)Cl_2$ (350 mg, 0.5 mmol) were dissolved in 1,4-dioxane (20 mL) and water (5 mL) under nitrogen. The mixture was then heated to 100°C and stirred for 4 h. 100 mL of EtOAc was added and the mixture solution was washed with water (30 mL×3). The organic layer was dried with Na_2SO_4, evaporated under reduced pressure and purified by column chromatography (petroleum ether/ethyl acetate =1:1) to obtain compound **21** as pale yellow solid. Yield 42%; MS (ESI) *m/z*: 384.10 $[M+H]^+$.

3. ^{1}H NMR spectra of intermediates

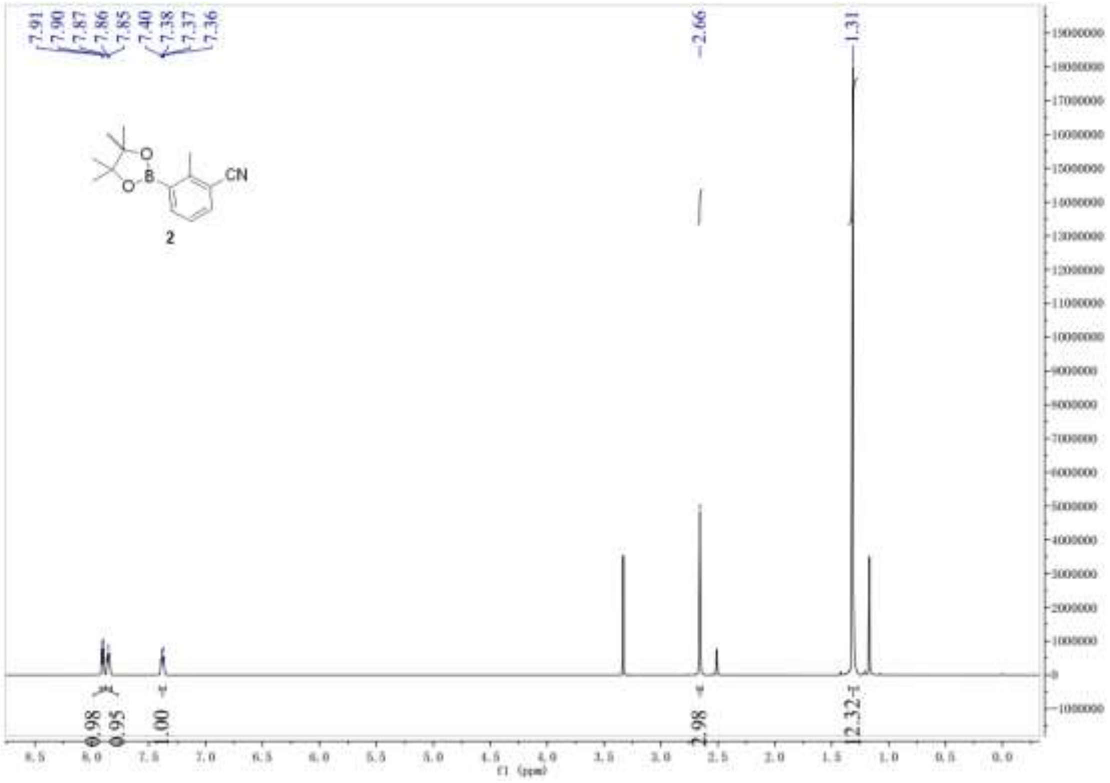

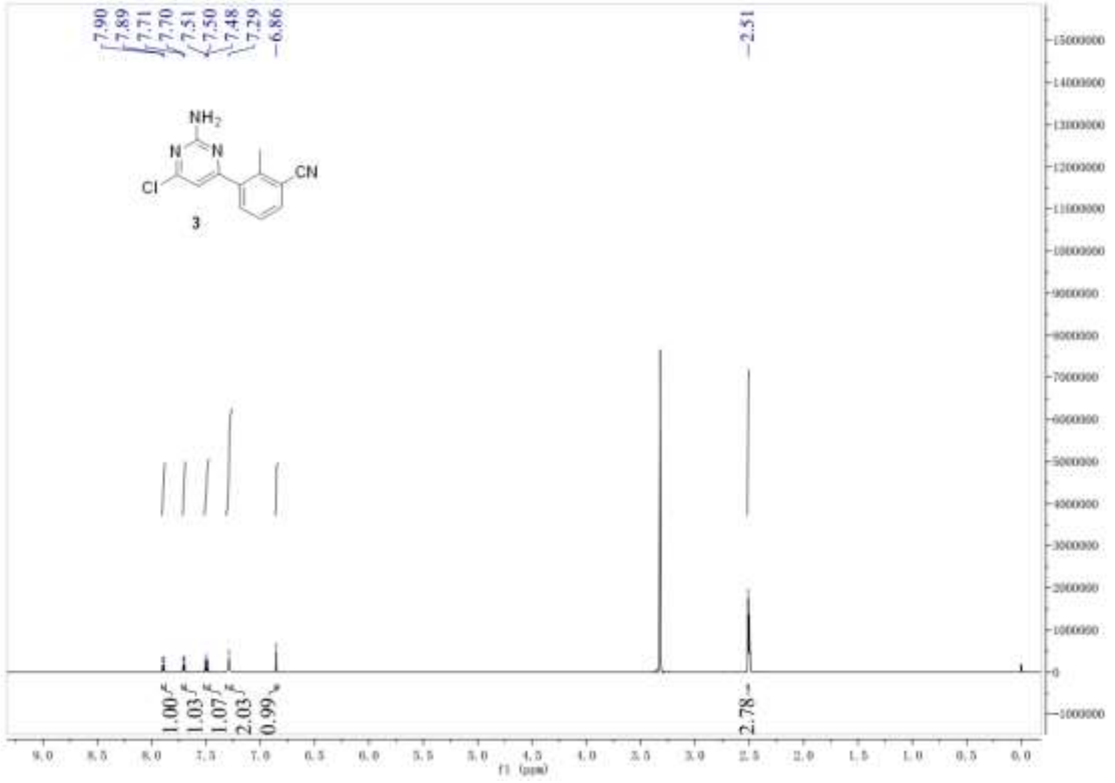

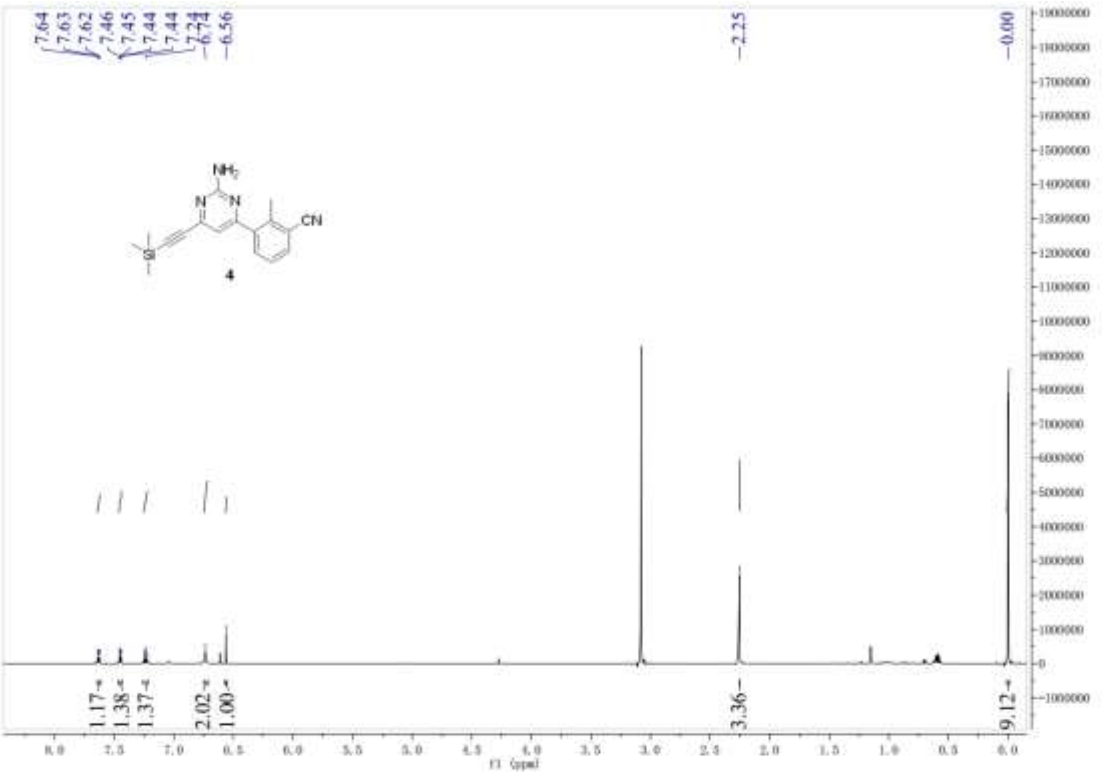

7.64
7.63
7.62
7.46
7.45
7.44
7.44
7.24
6.74
6.56
2.25
0.00
NH2
N
N
CN
Si
4
1.17
1.38
1.37
2.02
1.00
3.36
9.12
f1 (ppm)

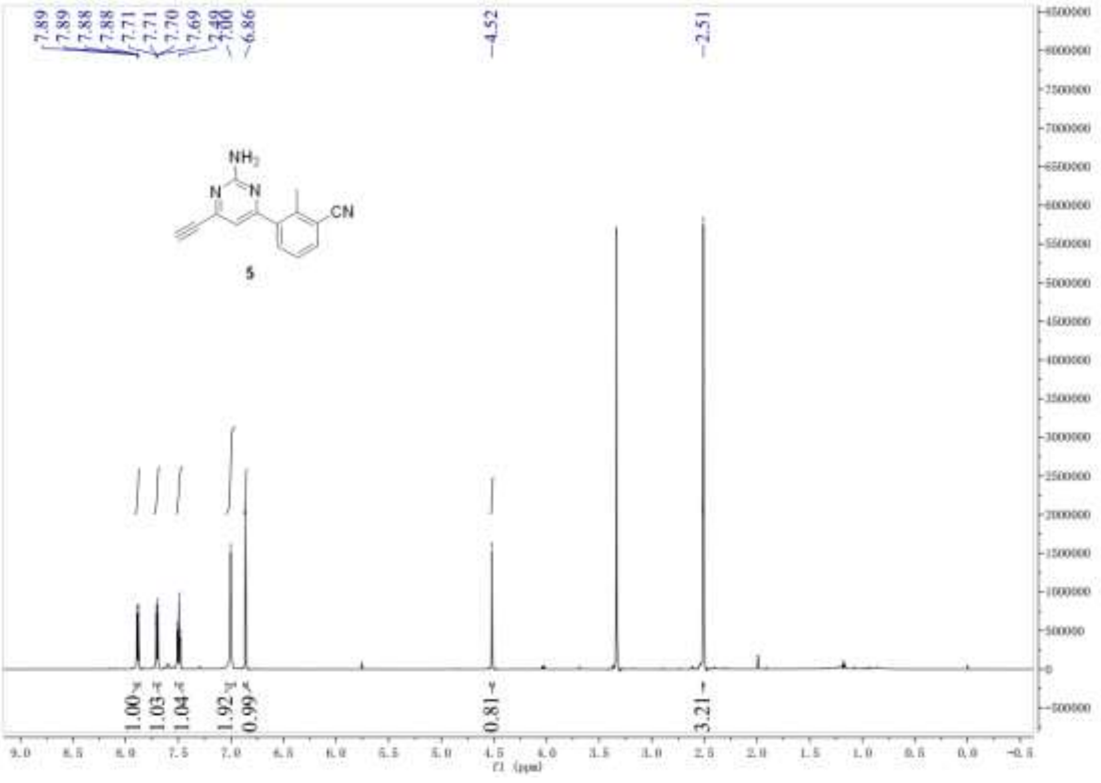

7.89
7.89
7.88
7.88
7.71
7.71
7.70
7.69
7.00
6.86
4.52
2.51
NH2
N
N
CN
5
1.00
1.03
1.04
1.92
0.99
0.81
3.21
f1 (ppm)

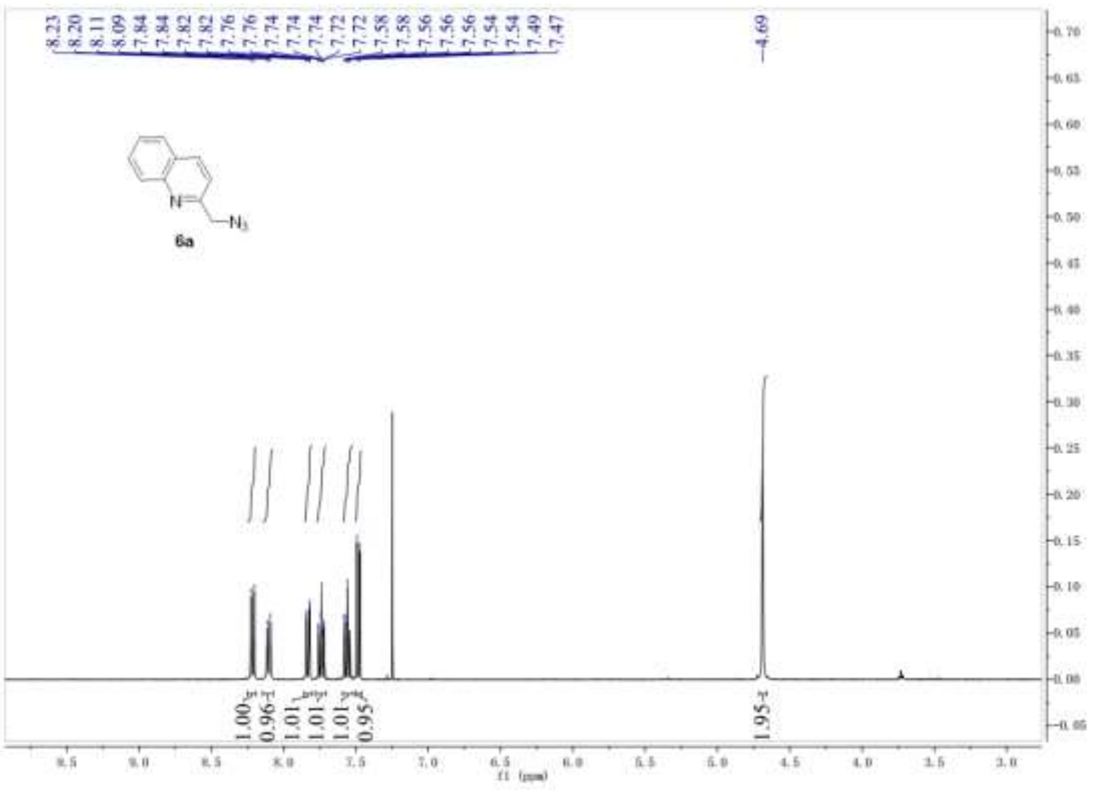
6a
N
N3
f1 (ppm)

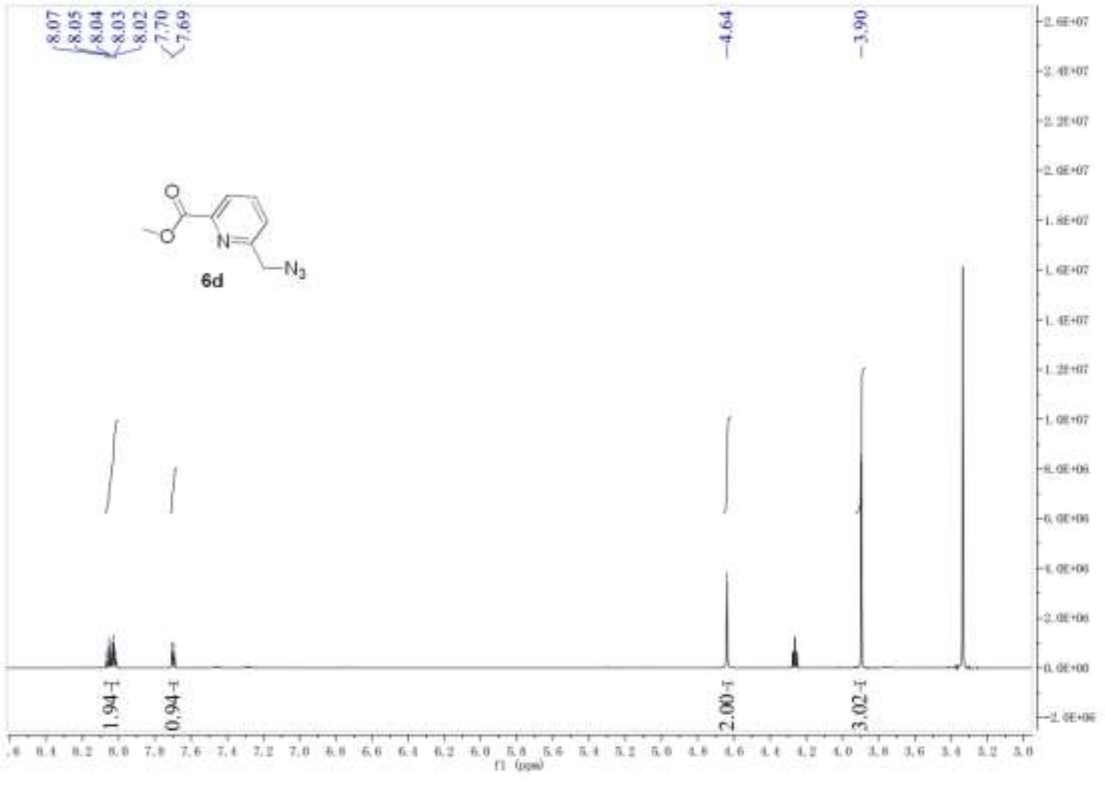
6d
O
N
N3
f1 (ppm)

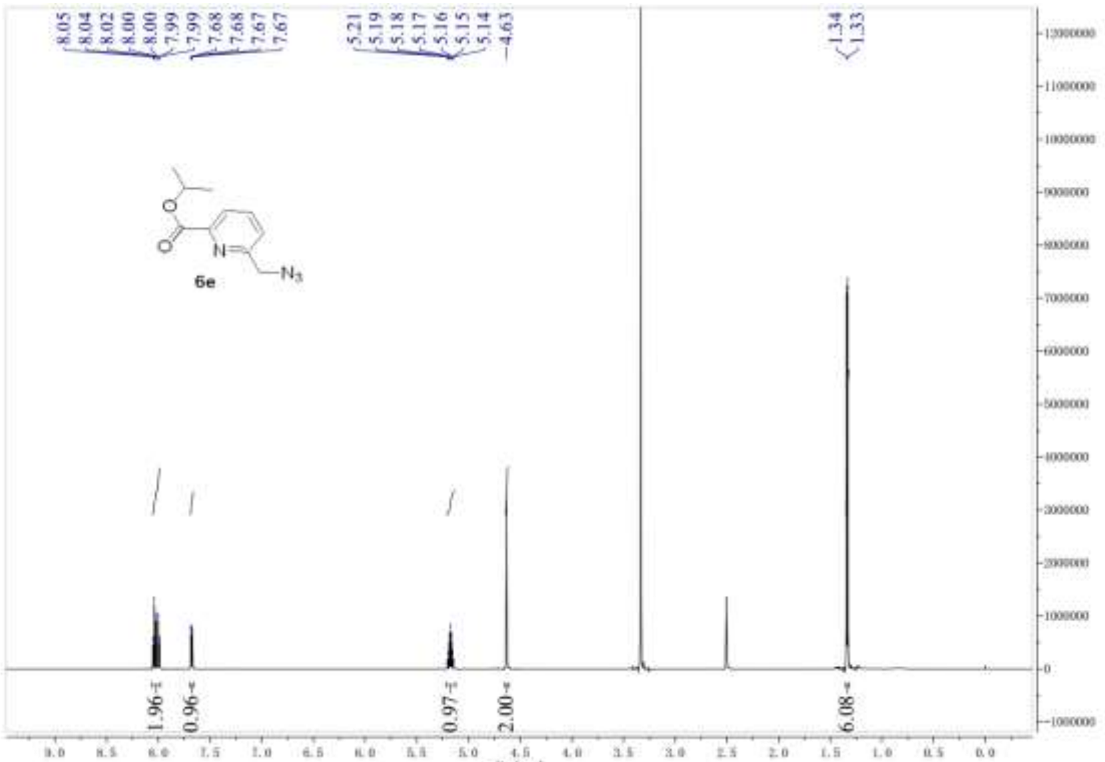

8.05
8.04
8.02
8.00
8.00
7.99
7.99
7.68
7.68
7.67
7.67
5.21
5.19
5.18
5.17
5.16
5.15
5.14
4.63
1.34
1.33
6e
N
O
O
N3
1.96
0.96
0.97
2.00
6.08
f1 (ppm)

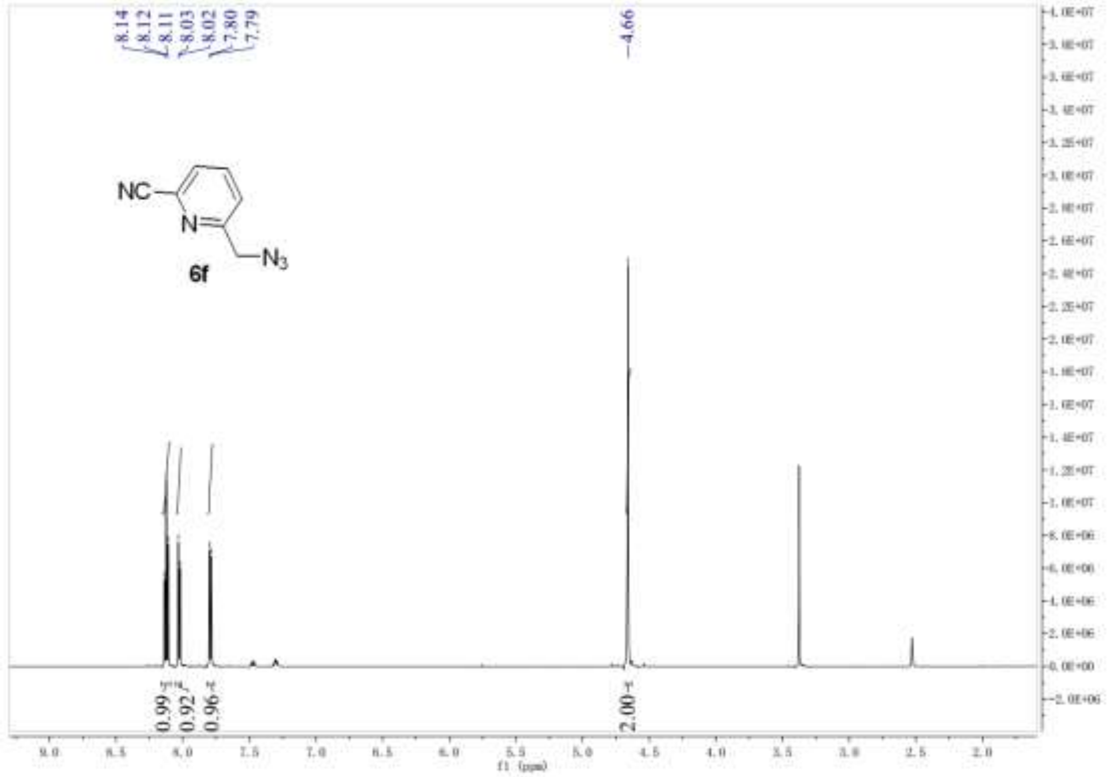

8.14
8.12
8.11
8.03
8.02
7.80
7.79
4.66
NC
N
N3
6f
0.99
0.92
0.96
2.00
f1 (ppm)

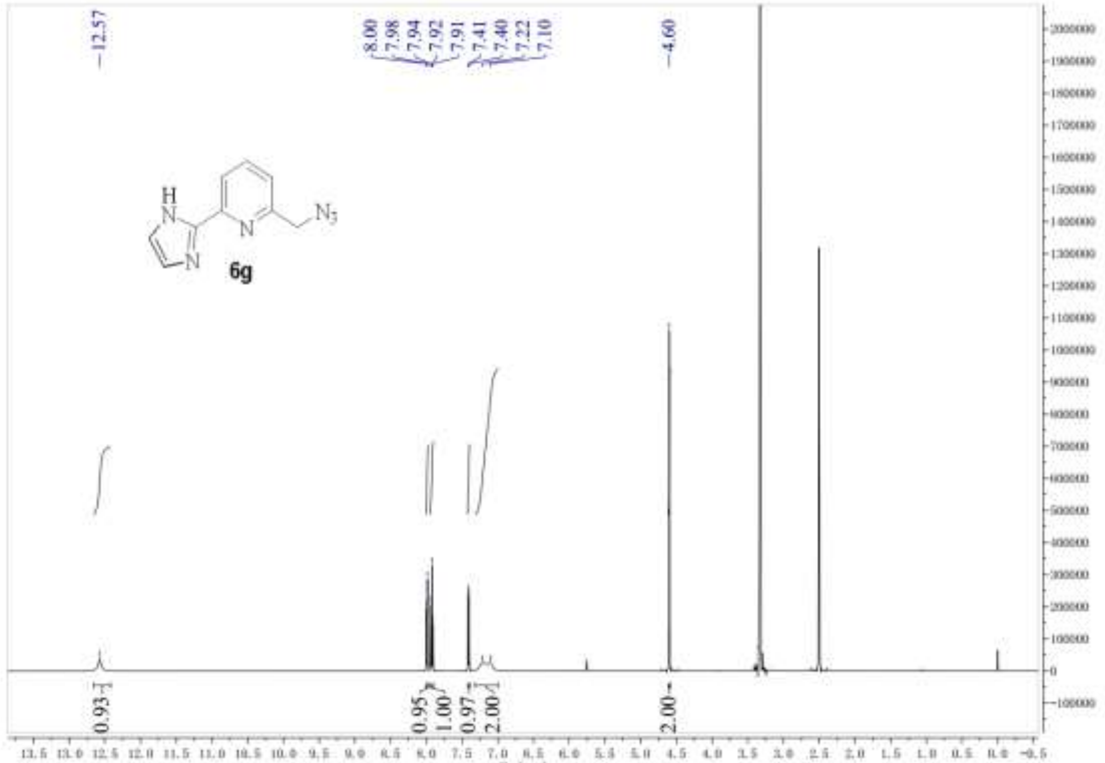
12.57
8.00
7.98
7.94
7.92
7.91
7.41
7.40
7.22
7.10
4.60
6g
0.93
0.95
1.00
0.97
2.00
2.00
f1 (ppm)

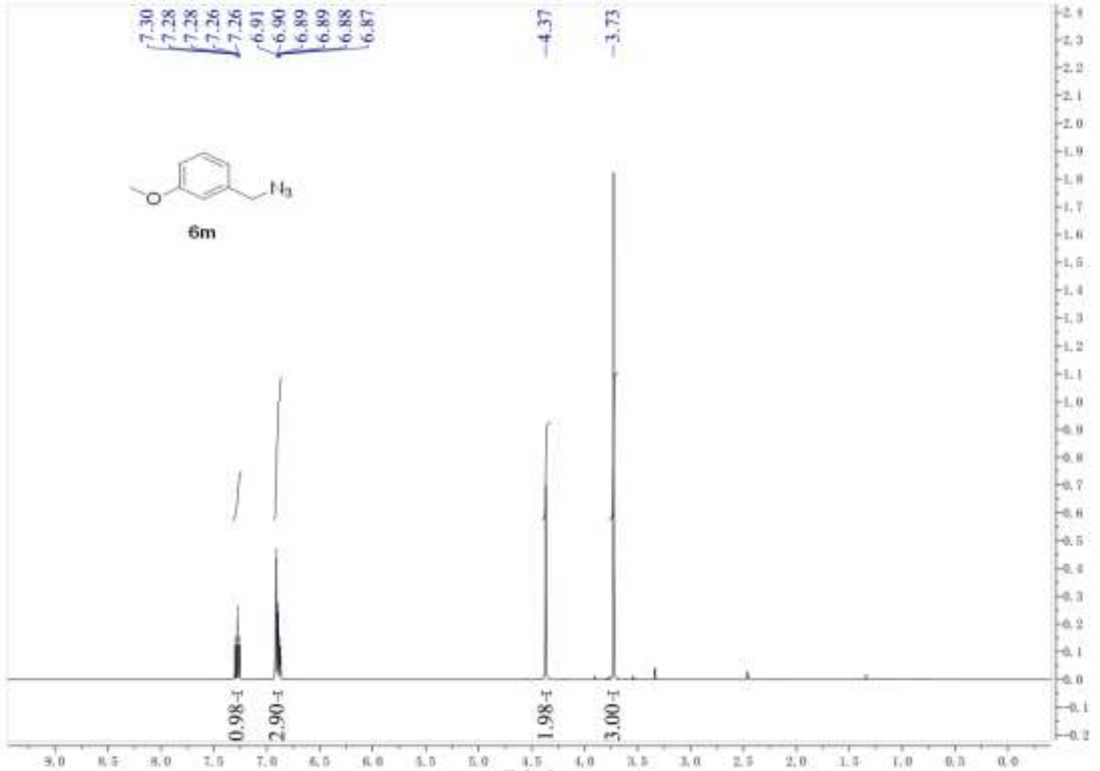
7.30
7.28
7.28
7.26
7.26
6.91
6.90
6.89
6.88
6.87
4.37
3.73
6m
0.98
2.90
1.98
3.00
f1 (ppm)

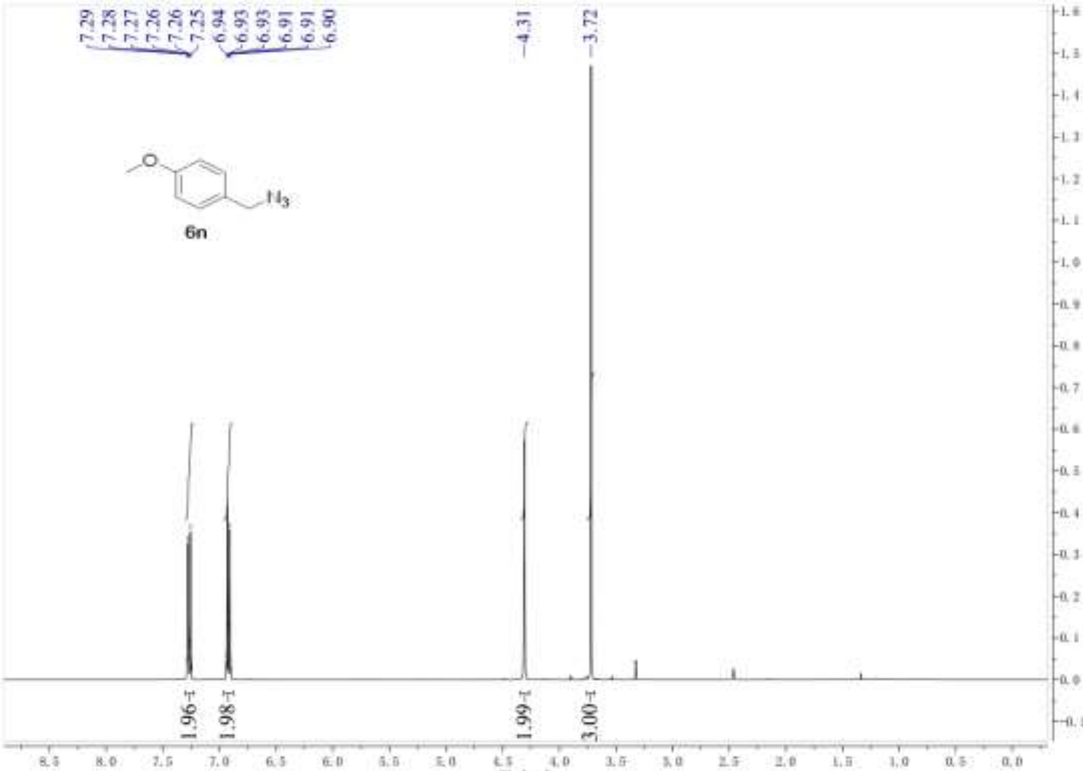
7.29
7.28
7.27
7.26
7.26
7.25
6.94
6.93
6.93
6.91
6.91
6.90
4.31
3.72
N3
6n
1.96
1.98
1.99
3.00
f1 (ppm)

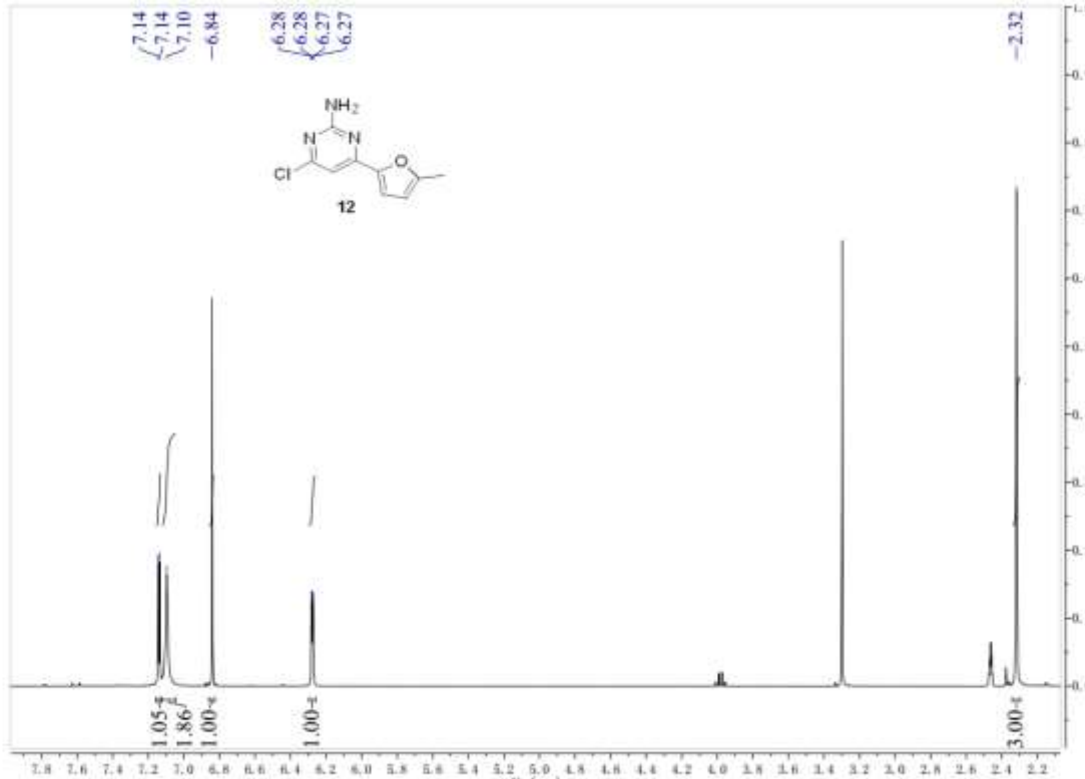
7.14
7.14
7.10
6.84
6.28
6.28
6.27
6.27
2.32
NH2
N
N
Cl
O
12
1.05
1.86
1.00
1.00
3.00
f1 (ppm)

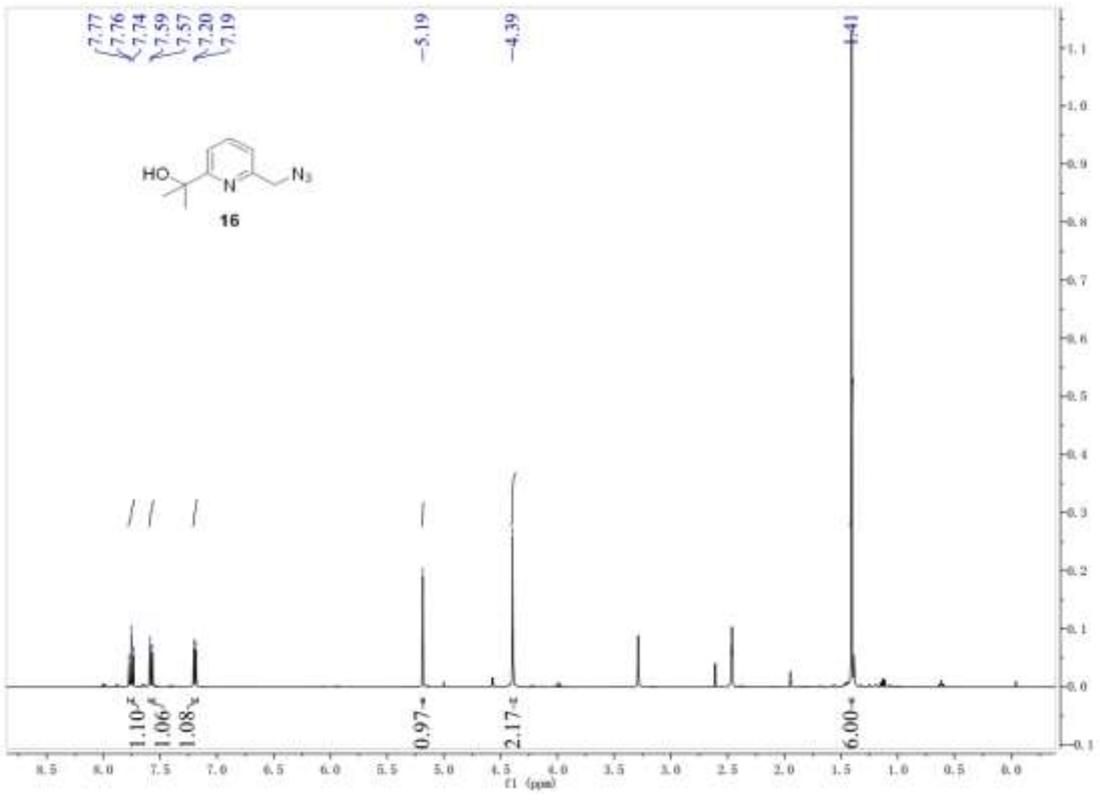
7.77
7.76
7.74
7.59
7.57
7.20
7.19
5.19
4.39
1.41
HO
N
N3
16
1.10
1.06
1.08
0.97
2.17
6.00
f1 (ppm)

4. NMR spectra of target compounds 7, 17, 22

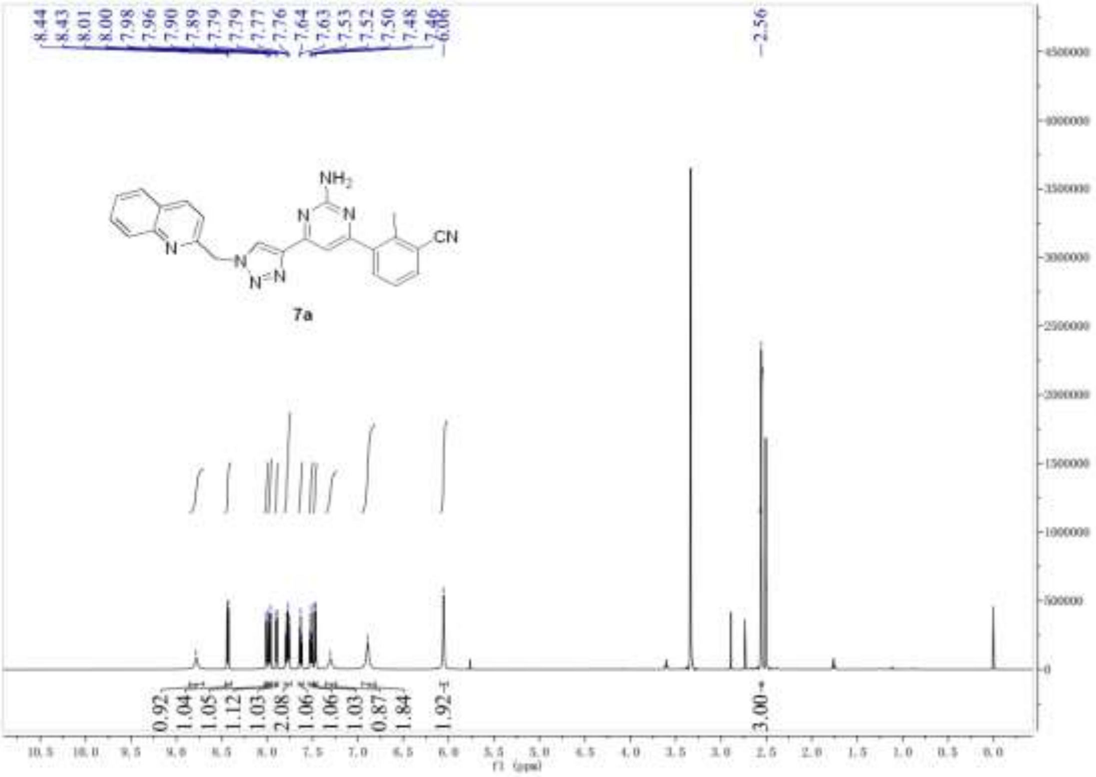

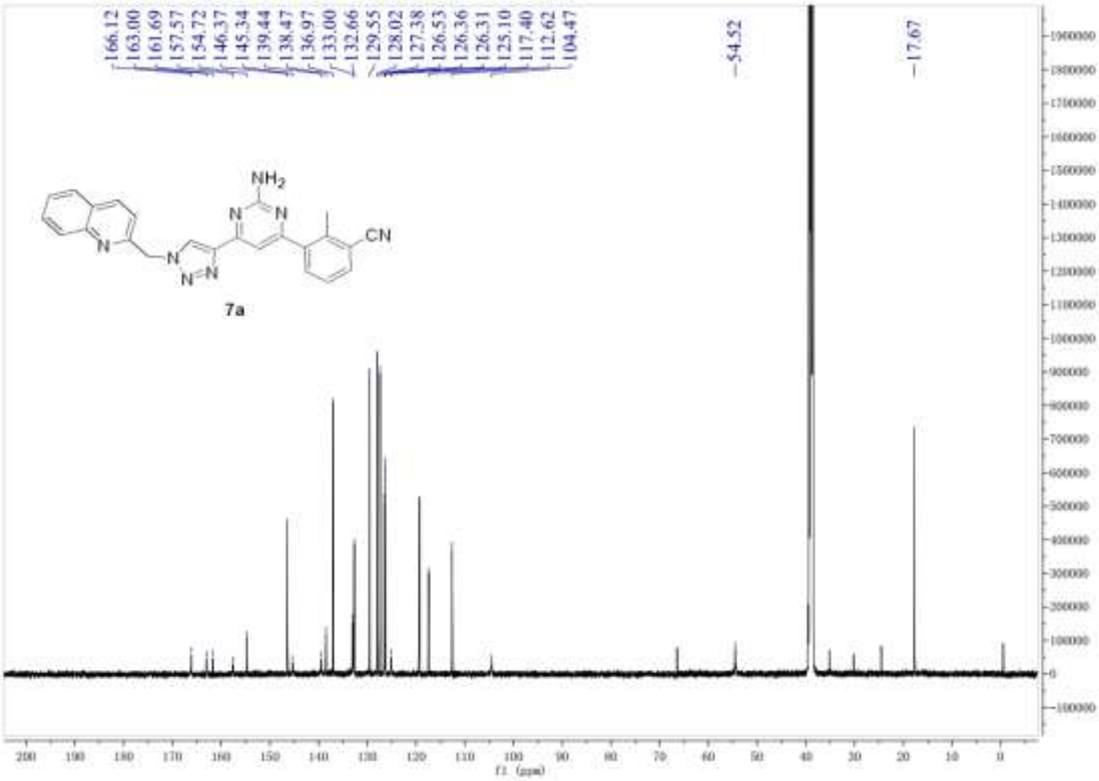

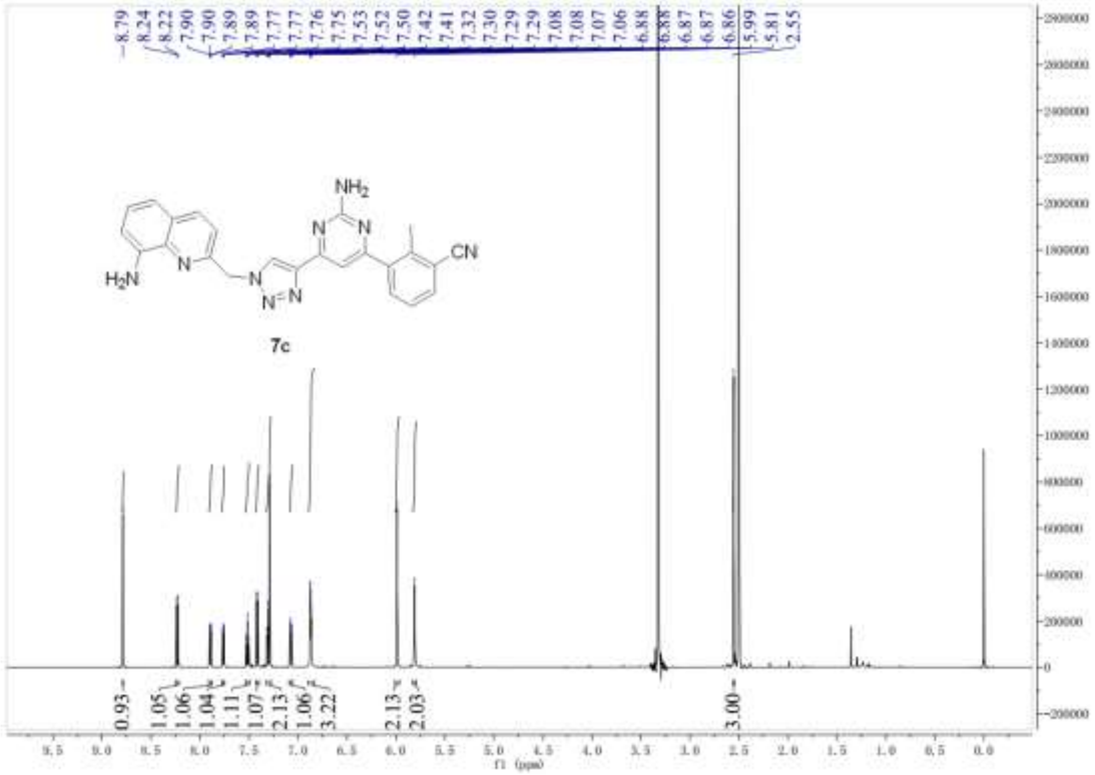
8.79 8.24 8.22 7.90 7.90 7.89 7.89 7.77 7.77 7.76 7.75 7.53 7.52 7.50 7.42 7.41 7.32 7.30 7.29 7.29 7.08 7.08 7.07 7.06 6.88 6.88 6.87 6.87 6.86 5.99 5.81 2.55
NH2
N
CN
H2N
7c
0.93 1.05 1.06 1.04 1.11 1.07 2.13 1.06 3.22 2.13 2.03 3.00
f1 (ppm)
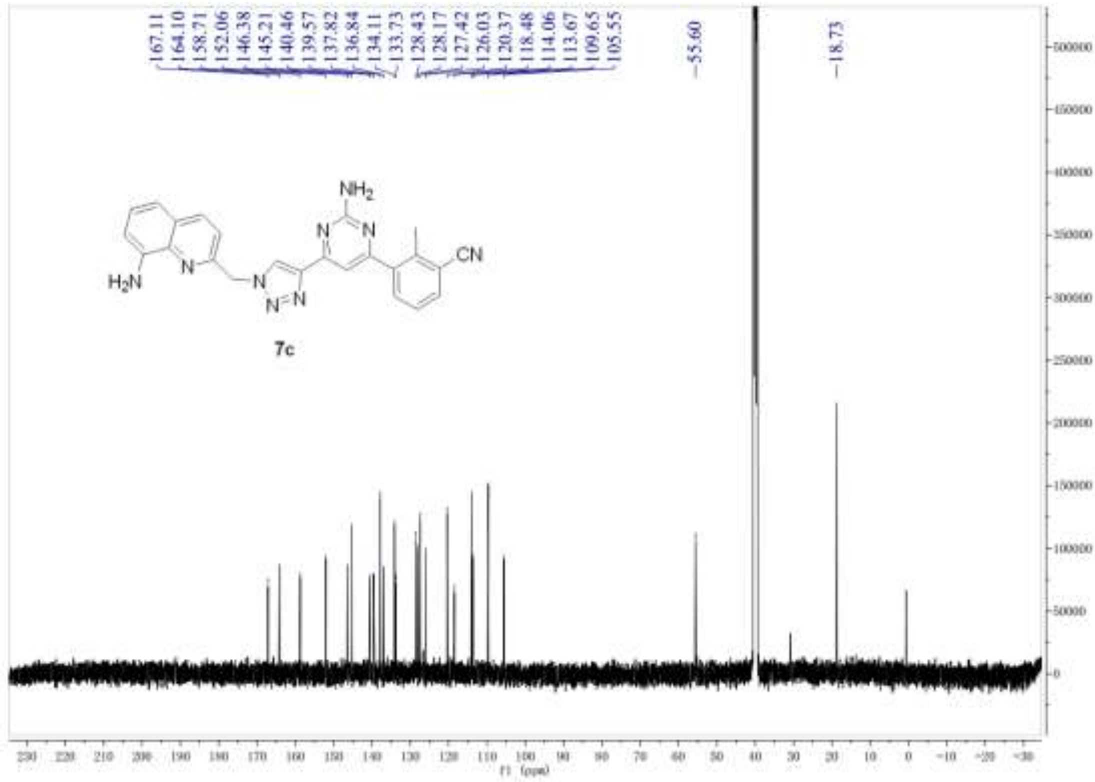
167.11 164.10 158.71 152.06 146.38 145.21 140.46 139.57 137.82 136.84 134.11 133.73 128.43 128.17 127.42 126.03 120.37 118.48 114.06 113.67 109.65 105.55
55.60
18.73
NH2
N
CN
H2N
7c
f1 (ppm)

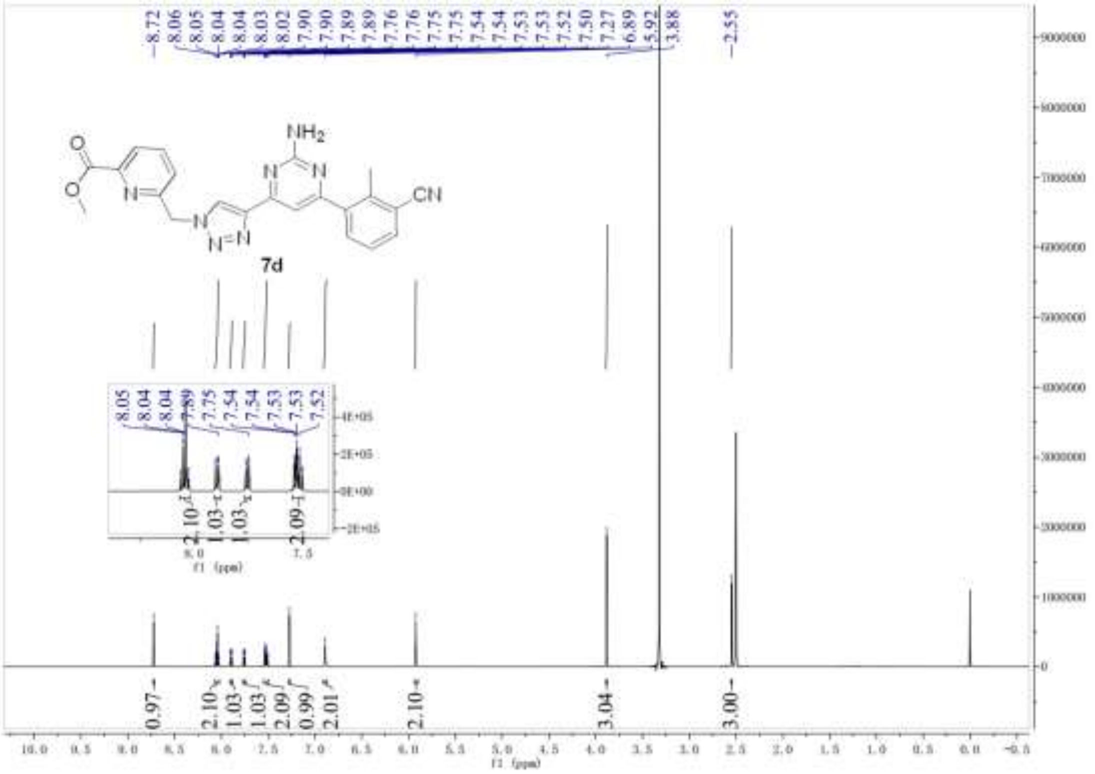
8.72
8.06
8.05
8.04
8.04
8.03
8.02
7.90
7.90
7.89
7.89
7.76
7.76
7.75
7.75
7.54
7.54
7.53
7.53
7.52
7.50
7.27
6.89
5.92
3.88
2.55
NH2
N
CN
N
N
N=N
O
O
7d
0.97
2.10
1.03
1.03
2.09
0.99
2.01
2.10
3.04
3.00
f1 (ppm)

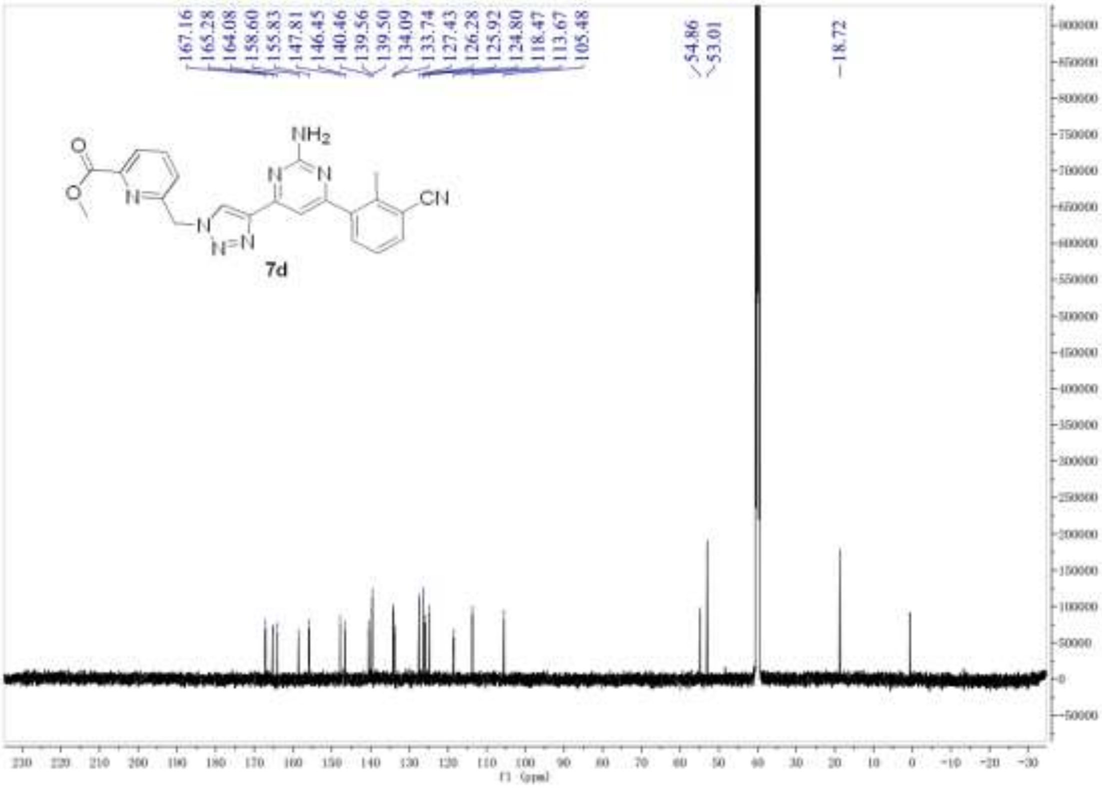
167.16
165.28
164.08
158.60
155.83
147.81
146.45
140.46
139.56
139.50
134.09
133.74
127.43
126.28
125.92
124.80
118.47
113.67
105.48
54.86
53.01
18.72
NH2
CN
7d
f1 (ppm)

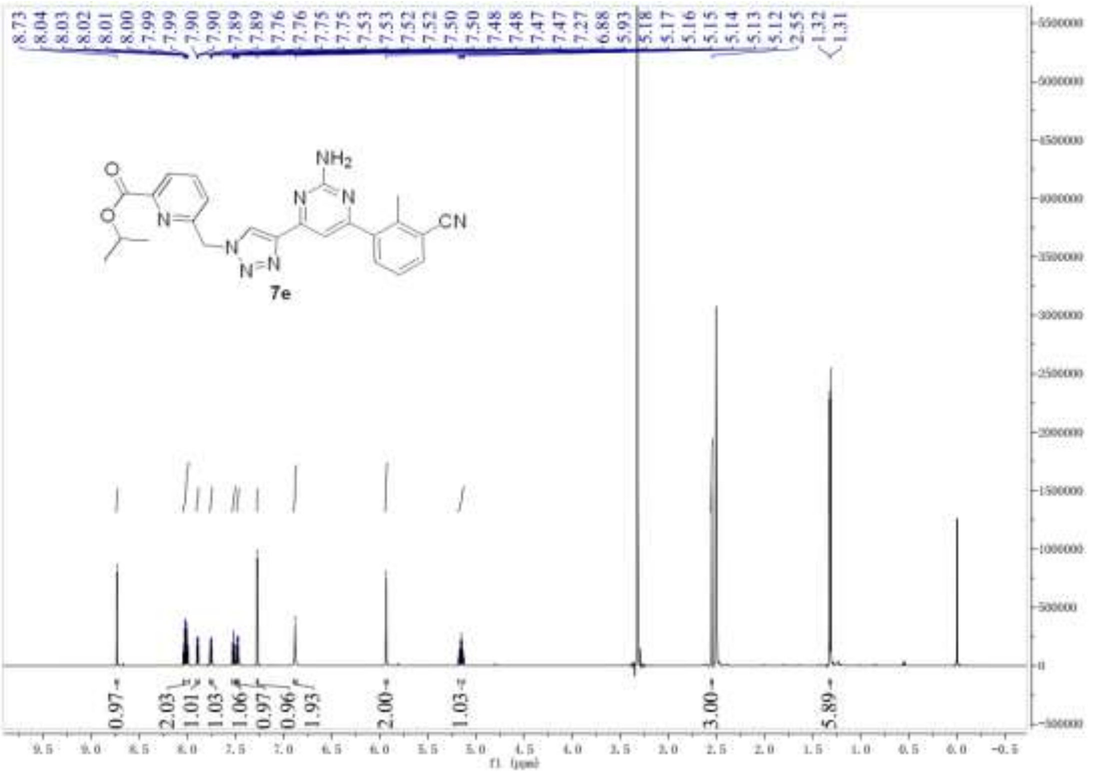

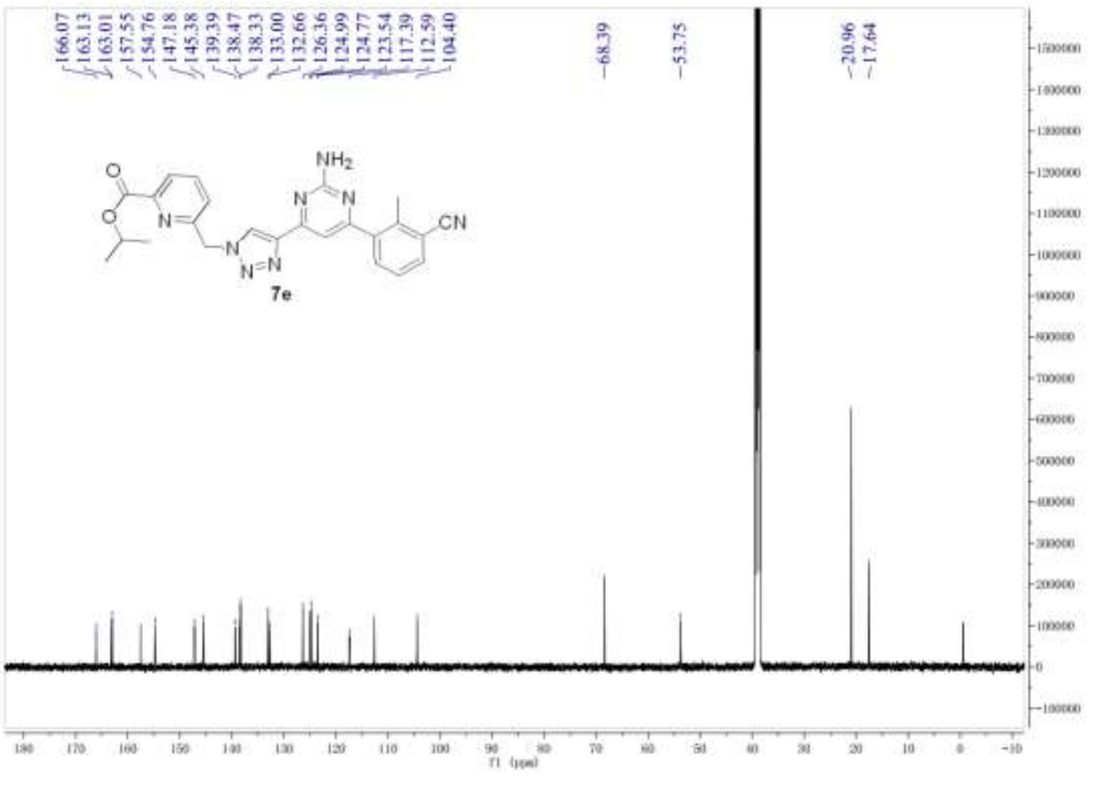

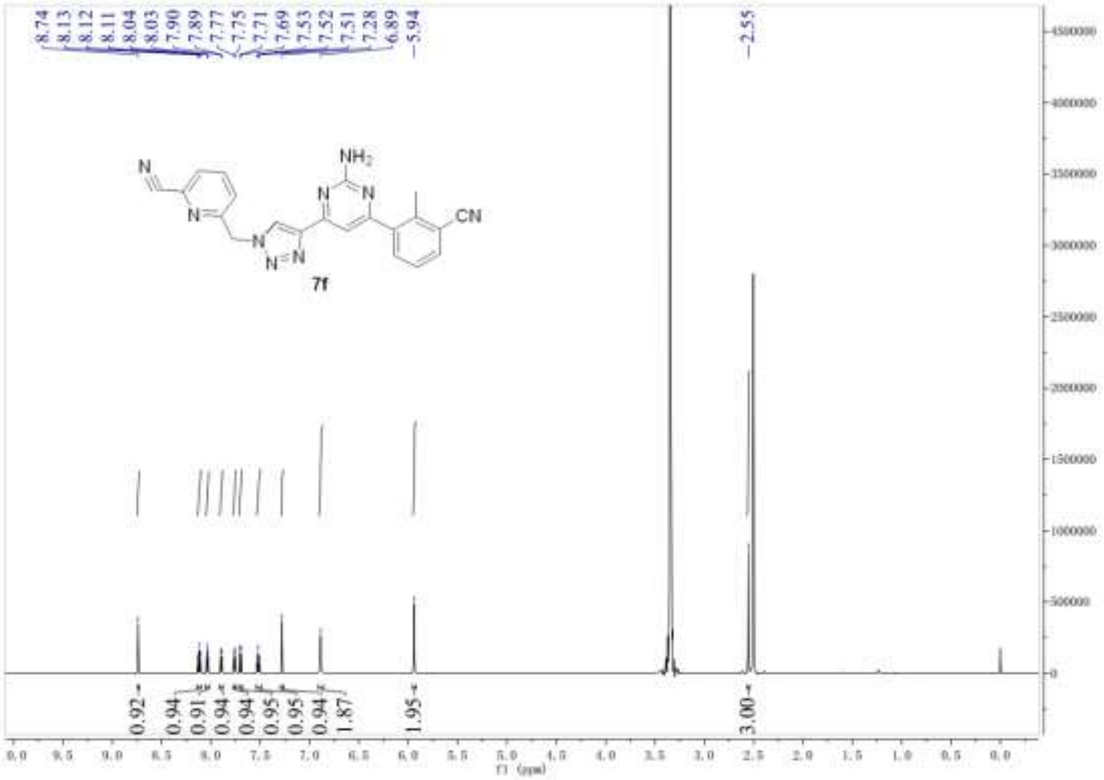
8.74
8.13
8.12
8.11
8.04
8.03
7.90
7.89
7.77
7.75
7.71
7.69
7.53
7.52
7.51
7.28
6.89
5.94
2.55
NH2
N
N
N
N
CN
N
N
N
7f
0.92
0.94
0.91
0.94
0.94
0.95
0.95
0.94
1.87
1.95
3.00
f1 (ppm)

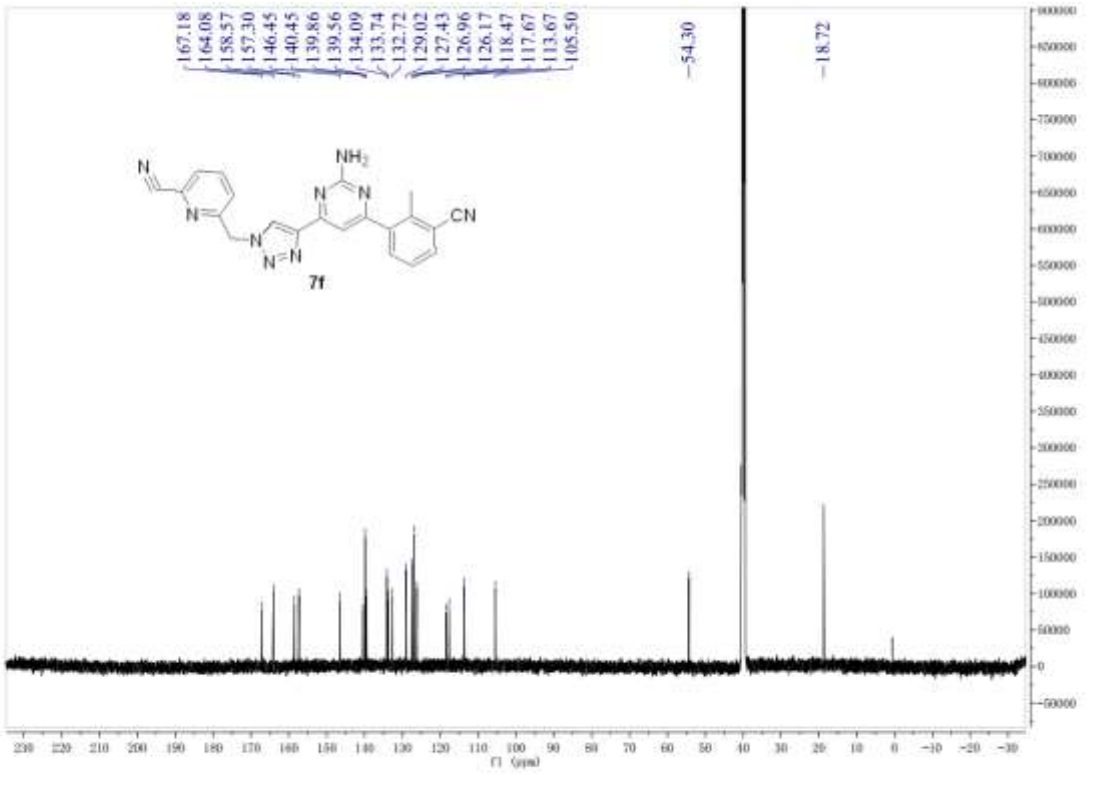
167.18
164.08
158.57
157.30
146.45
140.45
139.86
139.56
134.09
133.74
132.72
129.02
127.43
126.96
126.17
118.47
117.67
113.67
105.50
54.30
18.72
NH2
N
N
N
N
CN
N
N
N
7f
f1 (ppm)

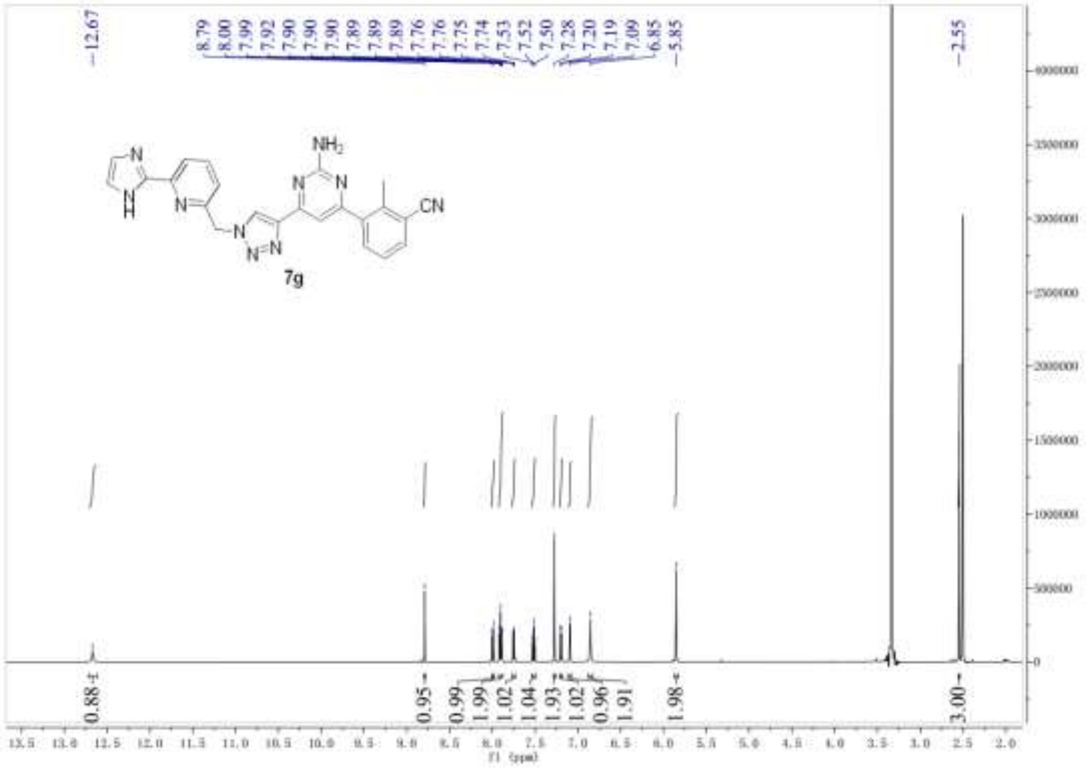
12.67
8.79
8.00
7.99
7.92
7.90
7.90
7.90
7.89
7.89
7.89
7.76
7.76
7.75
7.74
7.53
7.52
7.50
7.28
7.20
7.19
7.09
6.85
5.85
2.55
NH2
CN
7g
0.88
0.95
0.99
1.99
1.02
1.04
1.93
1.02
0.96
1.91
1.98
3.00
f1 (ppm)

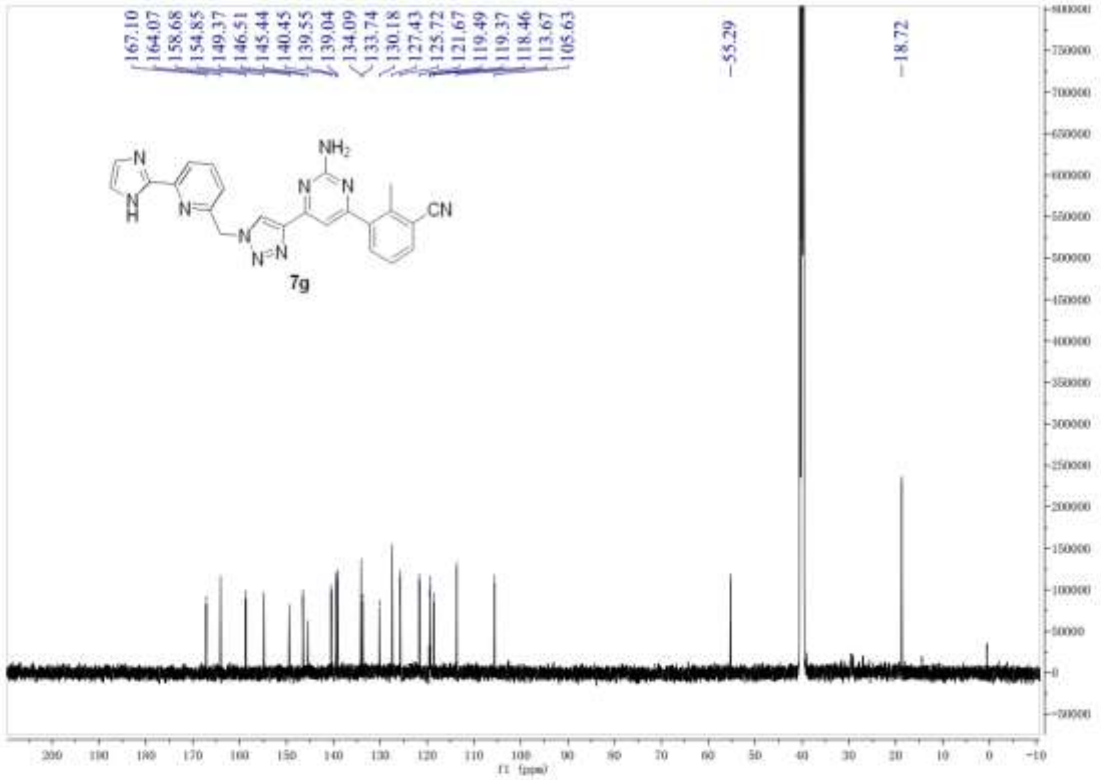
167.10
164.07
158.68
154.85
149.37
146.51
145.44
140.45
139.55
139.04
134.09
133.74
130.18
127.43
125.72
121.67
119.49
119.37
118.46
113.67
105.63
55.29
18.72
NH2
CN
7g
f1 (ppm)

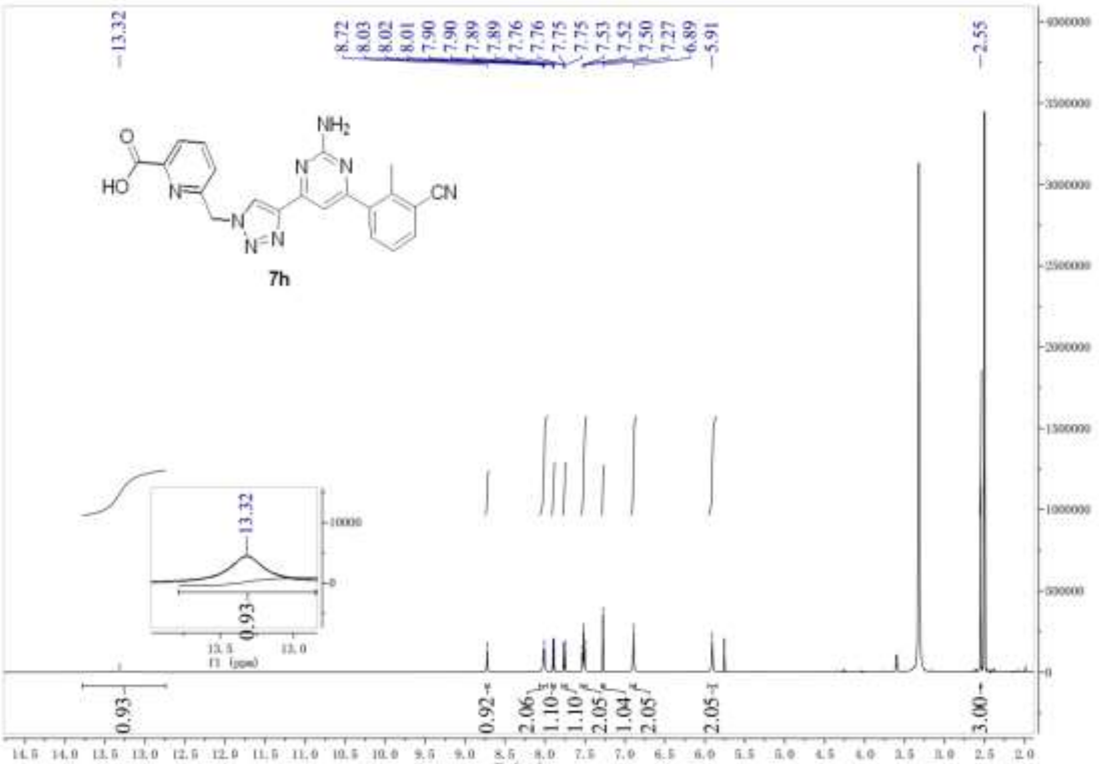
13.32
8.72 8.03 8.02 8.01 7.90 7.90 7.89 7.89 7.76 7.76 7.75 7.75 7.53 7.52 7.50 7.27 6.89 5.91
2.55
NH2
O
HO
N
CN
7h
0.93
0.92 2.06 1.10 1.10 2.05 1.04 2.05 2.05 3.00
f1 (ppm)

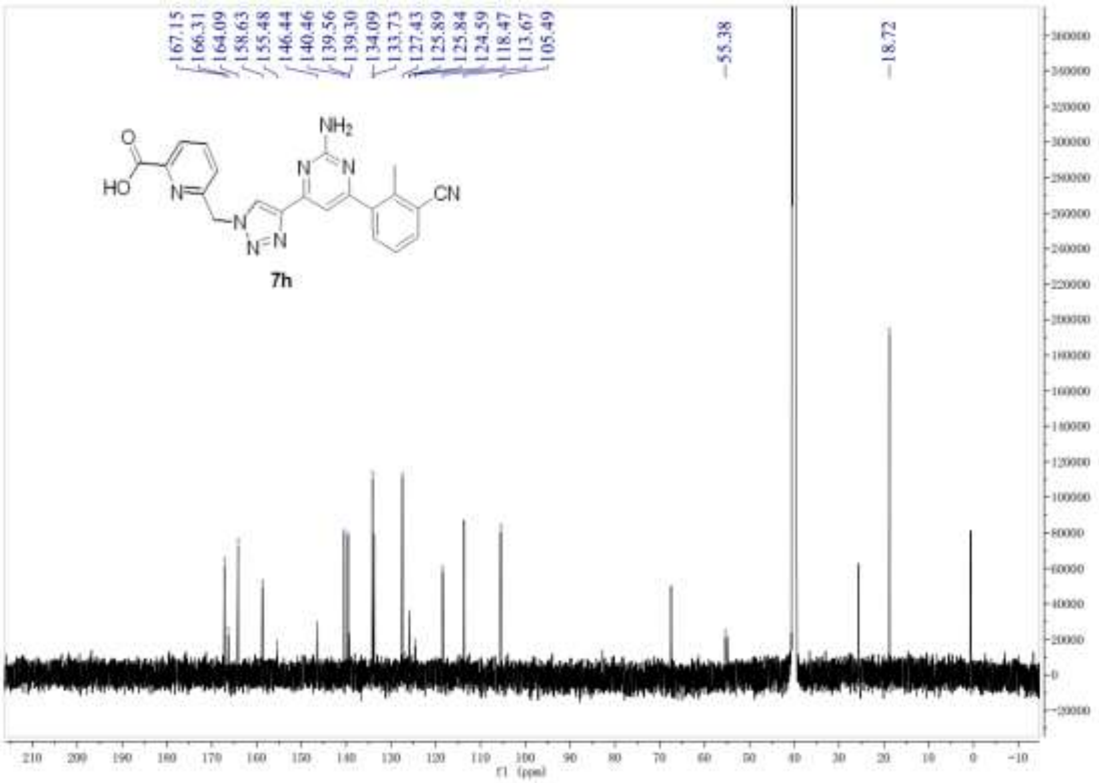
167.15 166.31 164.09 158.63 155.48 146.44 140.46 139.56 139.30 134.09 133.73 127.43 125.89 125.84 124.59 118.47 113.67 105.49
55.38
18.72
NH2
O
HO
N
CN
7h
f1 (ppm)

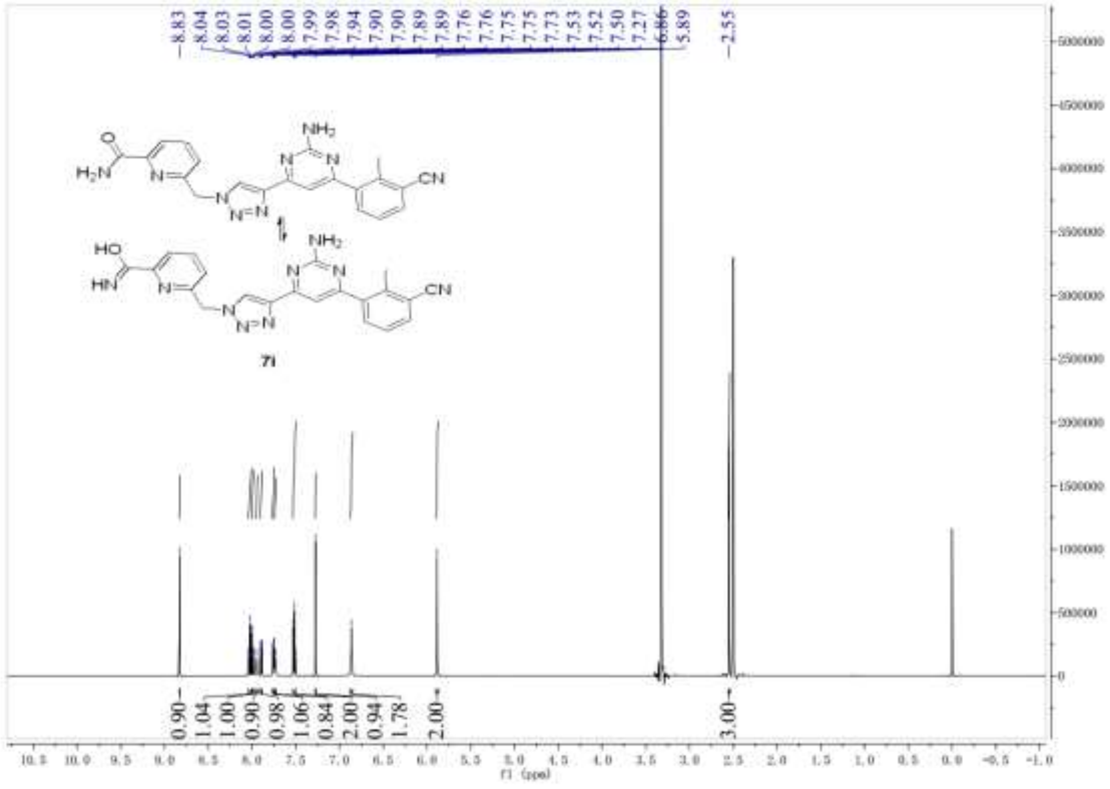

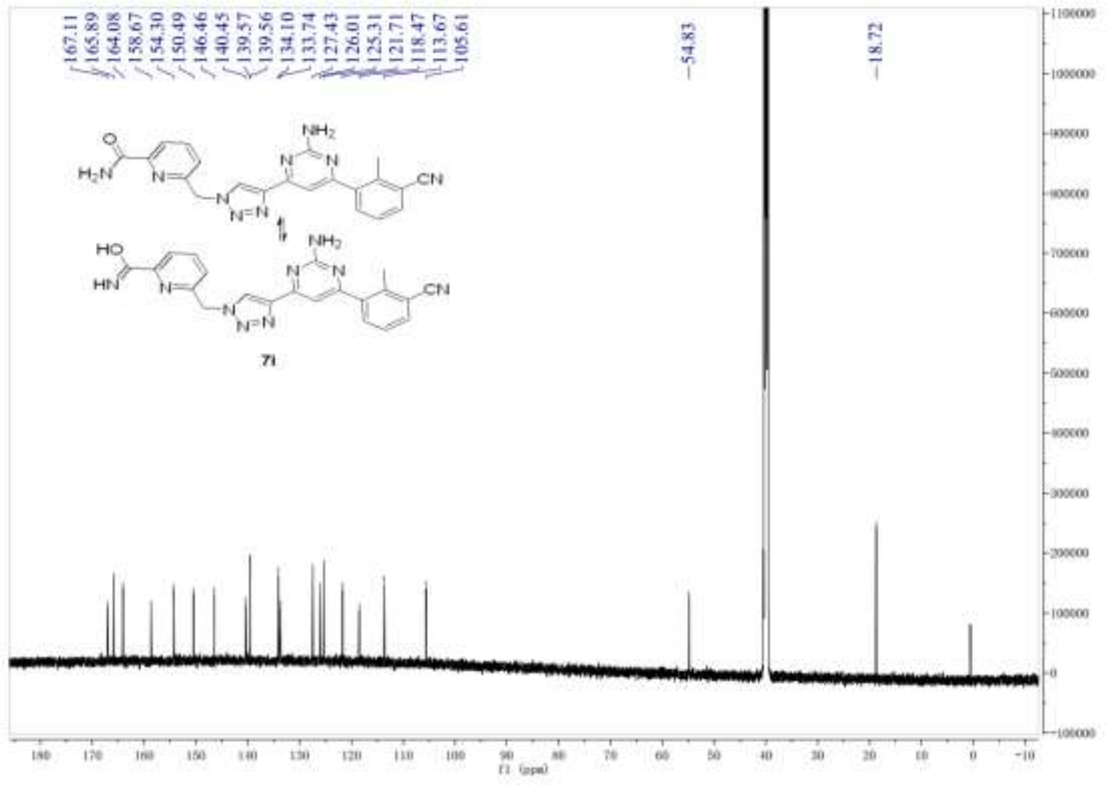

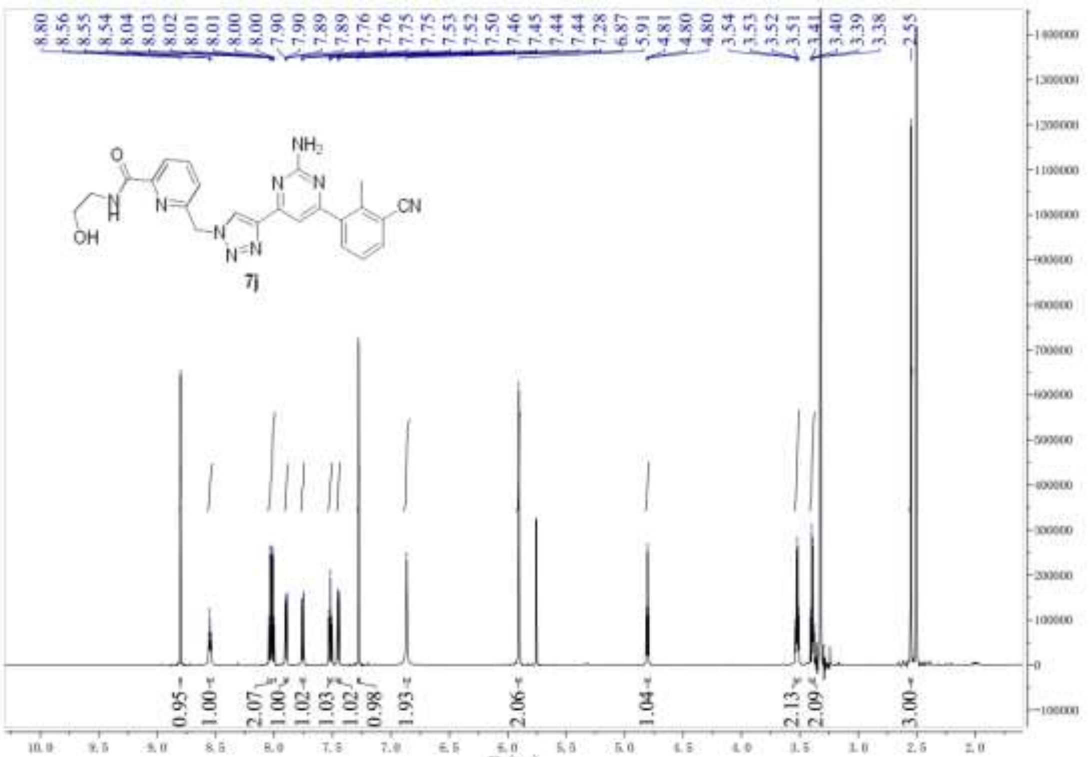
8.80
8.56
8.55
8.54
8.04
8.03
8.02
8.01
8.01
8.00
8.00
7.90
7.90
7.89
7.89
7.76
7.76
7.75
7.75
7.53
7.52
7.50
7.46
7.45
7.44
7.44
7.28
6.87
5.91
4.81
4.80
4.80
3.54
3.53
3.52
3.51
3.41
3.40
3.39
3.38
2.55
NH2
O
N
H
N
OH
N
N
N
N
N
CN
7j
0.95
1.00
2.07
1.00
1.02
1.03
1.02
0.98
1.93
2.06
1.04
2.13
2.09
3.00
f1 (ppm)

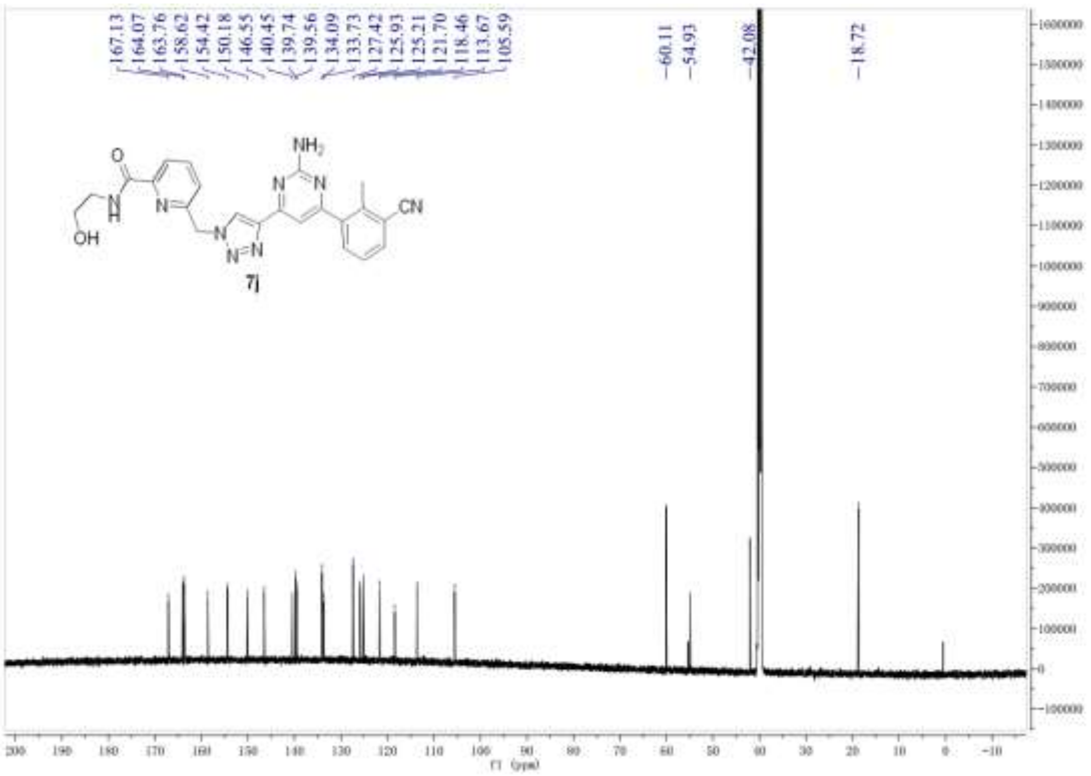
167.13
164.07
163.76
158.62
154.42
150.18
146.55
140.45
139.74
139.56
134.09
133.73
127.42
125.93
125.21
121.70
118.46
113.67
105.59
60.11
54.93
42.08
18.72
NH2
O
N
H
N
OH
N
N
N
N
N
CN
7j
f1 (ppm)

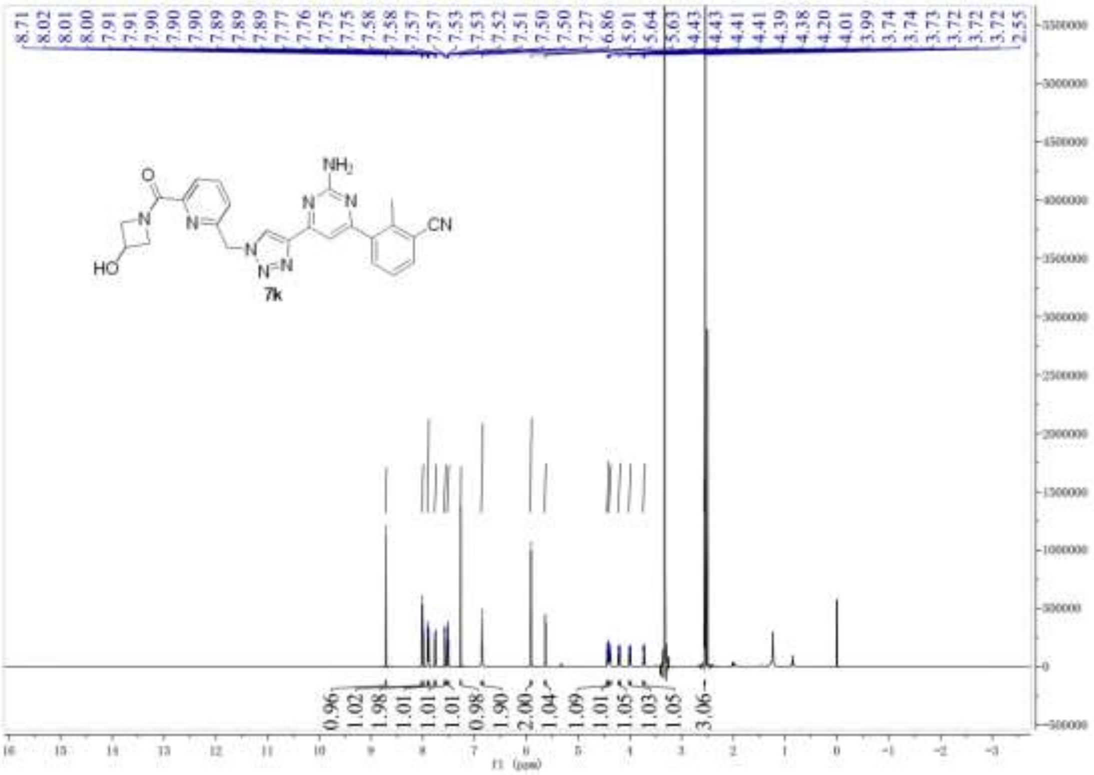
8.71
8.02
8.01
8.00
7.91
7.91
7.90
7.90
7.89
7.89
7.89
7.77
7.76
7.75
7.75
7.58
7.58
7.57
7.57
7.53
7.53
7.52
7.51
7.50
7.50
7.27
6.86
5.91
5.64
5.63
4.43
4.43
4.41
4.41
4.39
4.38
4.20
4.01
3.99
3.74
3.74
3.73
3.72
3.72
3.72
2.55
NH2
N
N
O
N
N
CN
N
N
N
HO
7k
0.96
1.02
1.98
1.01
1.01
1.01
0.98
1.90
2.00
1.04
1.09
1.01
1.05
1.03
1.05
3.06
f1 (ppm)

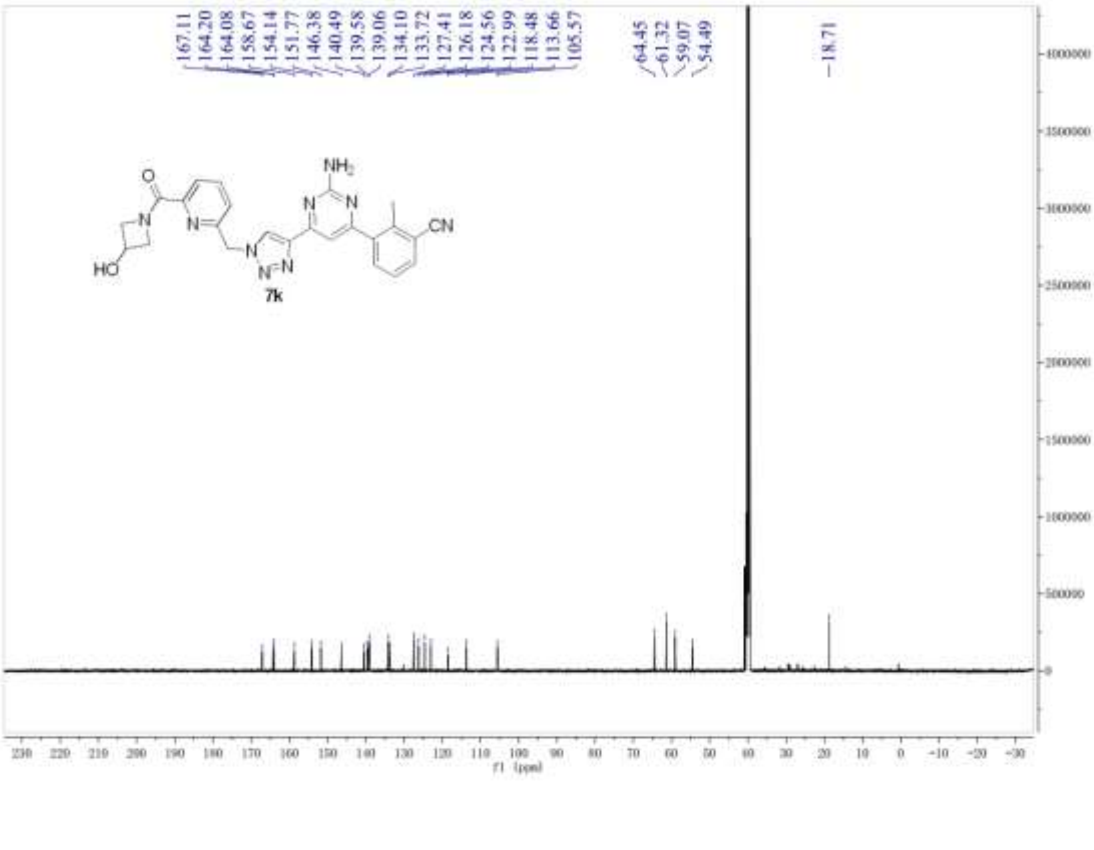
167.11
164.20
164.08
158.67
154.14
151.77
146.38
140.49
139.58
139.06
134.10
133.72
127.41
126.18
124.56
122.99
118.48
113.66
105.57
64.45
61.32
59.07
54.49
18.71
NH2
N
N
O
N
N
CN
N
N
N
HO
7k
f1 (ppm)

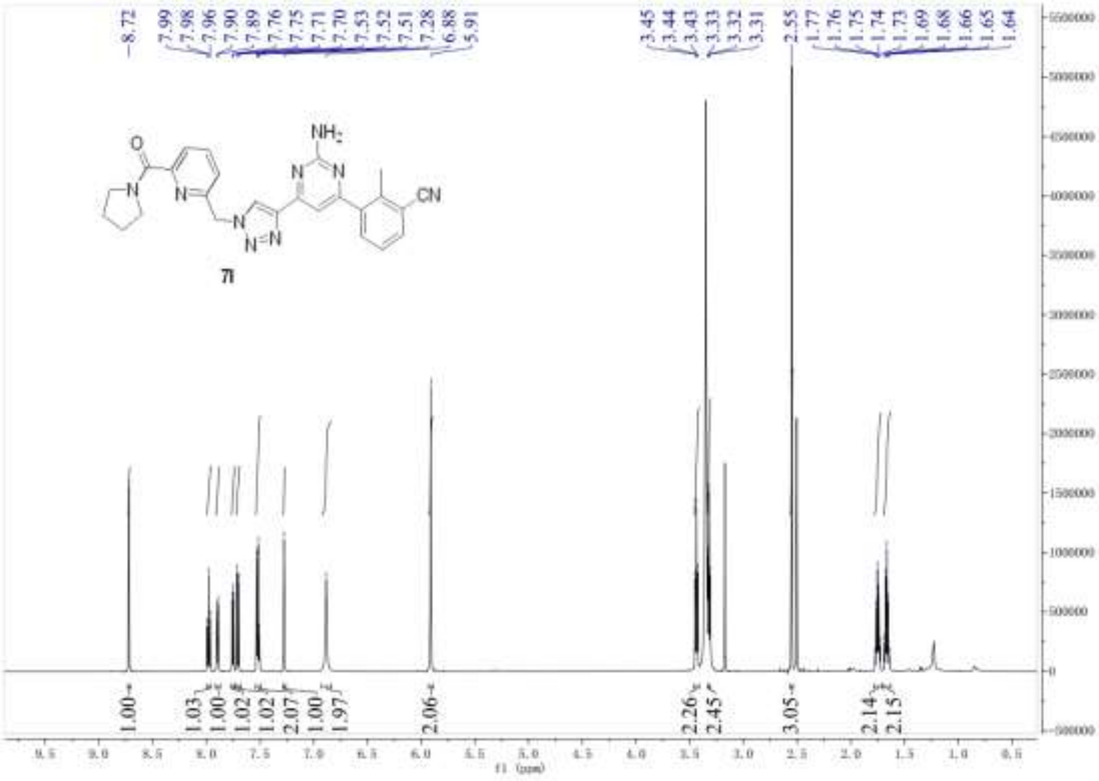
8.72
7.99
7.98
7.96
7.90
7.89
7.76
7.75
7.71
7.70
7.53
7.52
7.51
7.28
6.88
5.91
3.45
3.44
3.43
3.33
3.32
3.31
2.55
1.77
1.76
1.75
1.74
1.73
1.69
1.68
1.66
1.65
1.64
NH2
O
N
N
N
N
N
N=N
CN
7l
1.00
1.03
1.00
1.02
1.02
2.07
1.00
1.97
2.06
2.26
2.45
3.05
2.14
2.15
f1 (ppm)

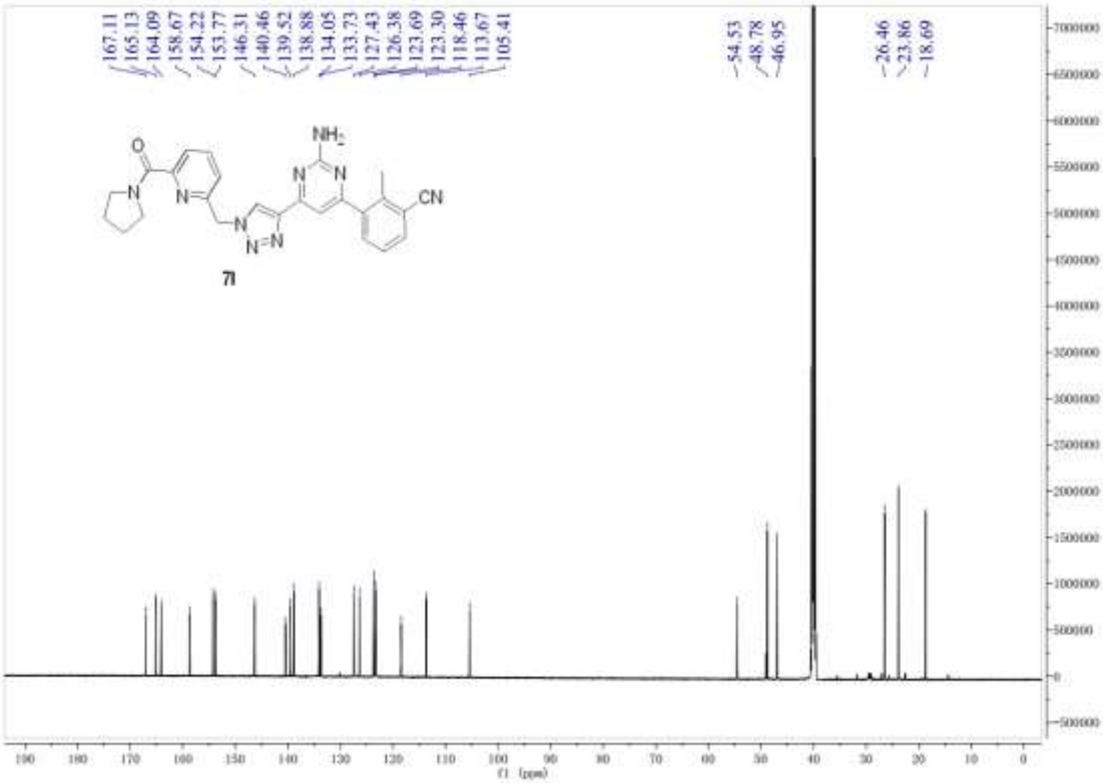
167.11
165.13
164.09
158.67
154.22
153.77
146.31
140.46
139.52
138.88
134.05
133.73
127.43
126.38
123.69
123.30
118.46
113.67
105.41
54.53
48.78
46.95
26.46
23.86
18.69
NH2
O
N
N
N
N
N
N=N
CN
7l
f1 (ppm)

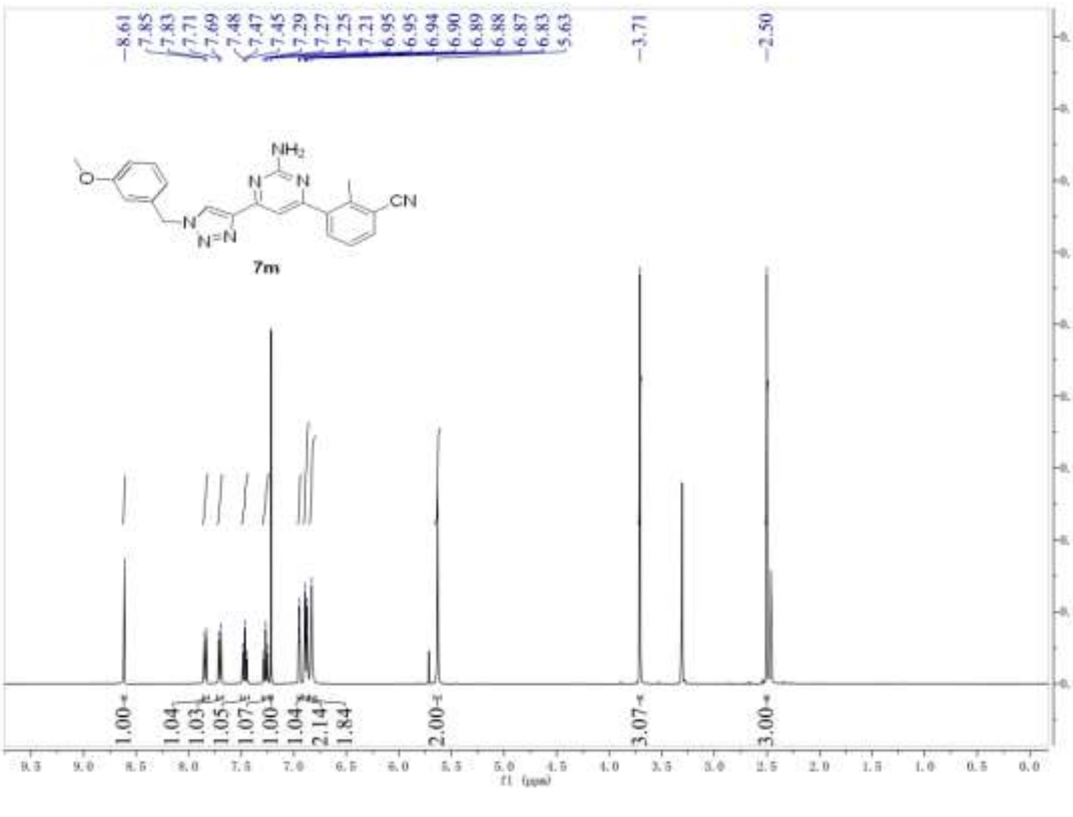
8.61
7.85
7.83
7.71
7.69
7.48
7.47
7.45
7.29
7.27
7.25
7.21
6.95
6.95
6.94
6.90
6.89
6.88
6.87
6.83
5.63
3.71
2.50
NH2
N
N
CN
O
N
N=N
7m
1.00
1.04
1.03
1.05
1.07
1.00
1.04
2.14
1.84
2.00
3.07
3.00
f1 (ppm)

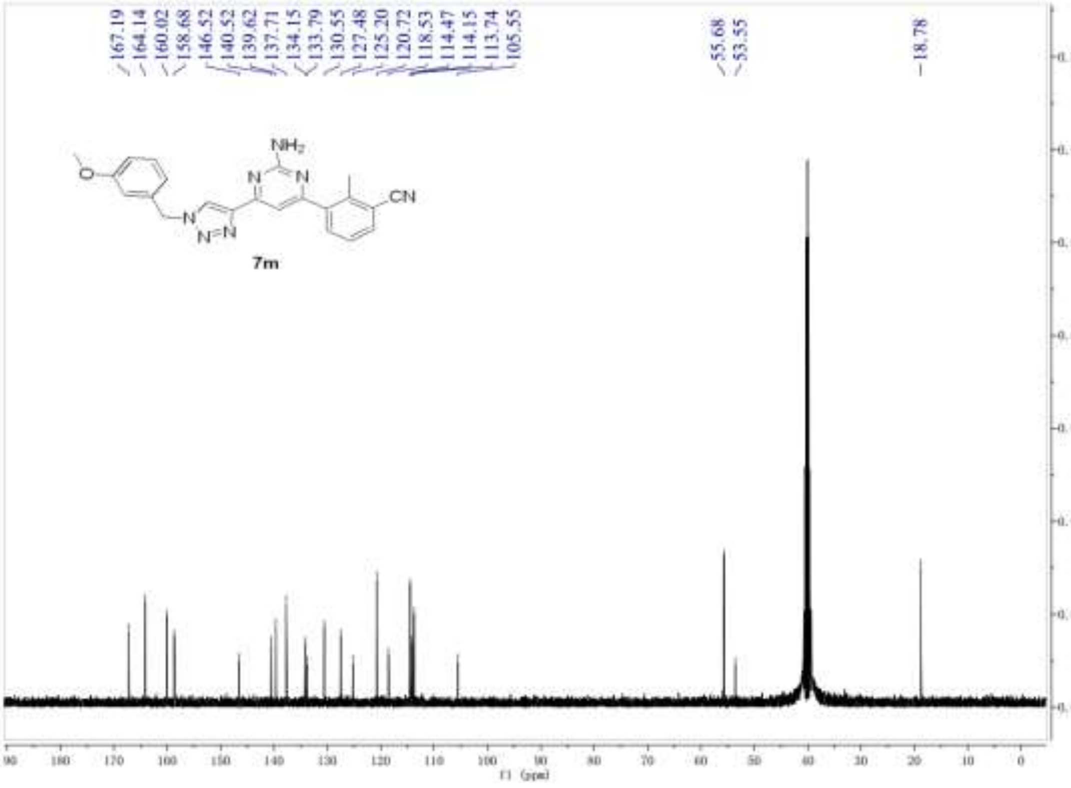
167.19
164.14
160.02
158.68
146.52
140.52
139.62
137.71
134.15
133.79
130.55
127.48
125.20
120.72
118.53
114.47
114.15
113.74
105.55
55.68
53.55
18.78
NH2
N
N
CN
O
N
N=N
7m
f1 (ppm)

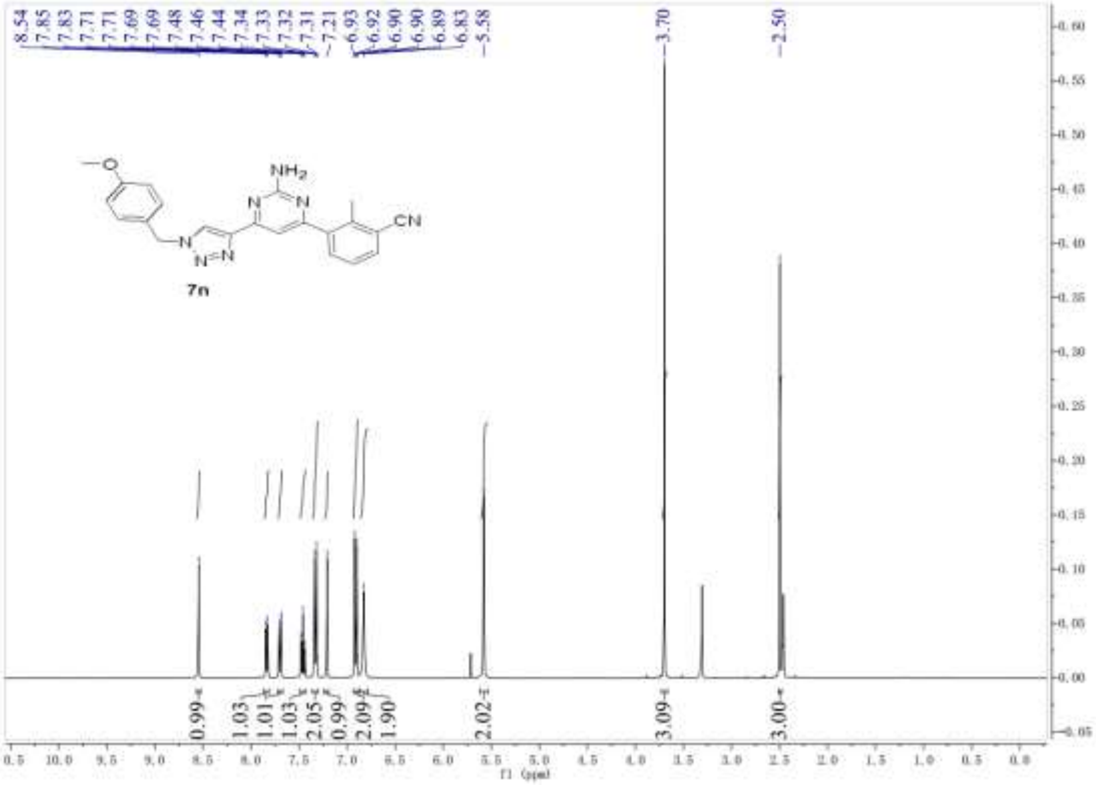

8.54
7.85
7.83
7.71
7.71
7.69
7.69
7.48
7.46
7.44
7.34
7.33
7.32
7.31
7.21
6.93
6.92
6.90
6.90
6.89
6.83
5.58
3.70
2.50
NH2
N
N
CN
N
N=N
7n
0.99
1.03
1.01
1.03
2.05
0.99
2.09
1.90
2.02
3.09
3.00
f1 (ppm)

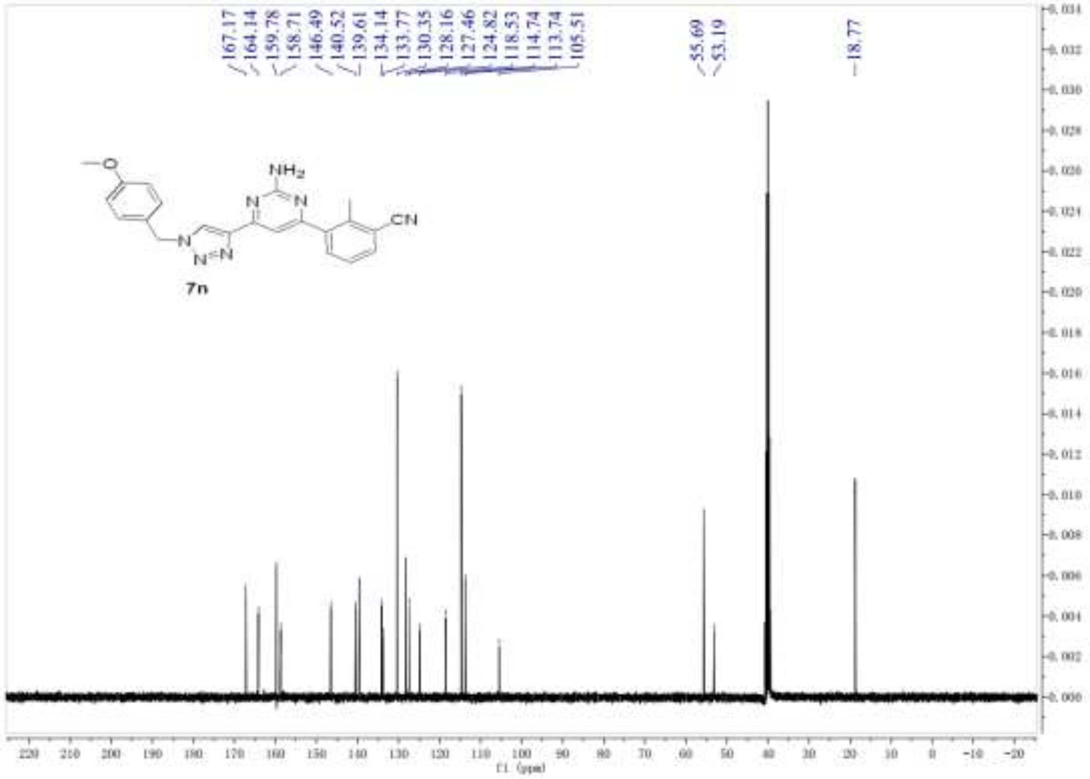

167.17
164.14
159.78
158.71
146.49
140.52
139.61
134.14
133.77
130.35
128.16
127.46
124.82
118.53
114.74
113.74
105.51
55.69
53.19
18.77
NH2
N
N
CN
N
N=N
7n
f1 (ppm)

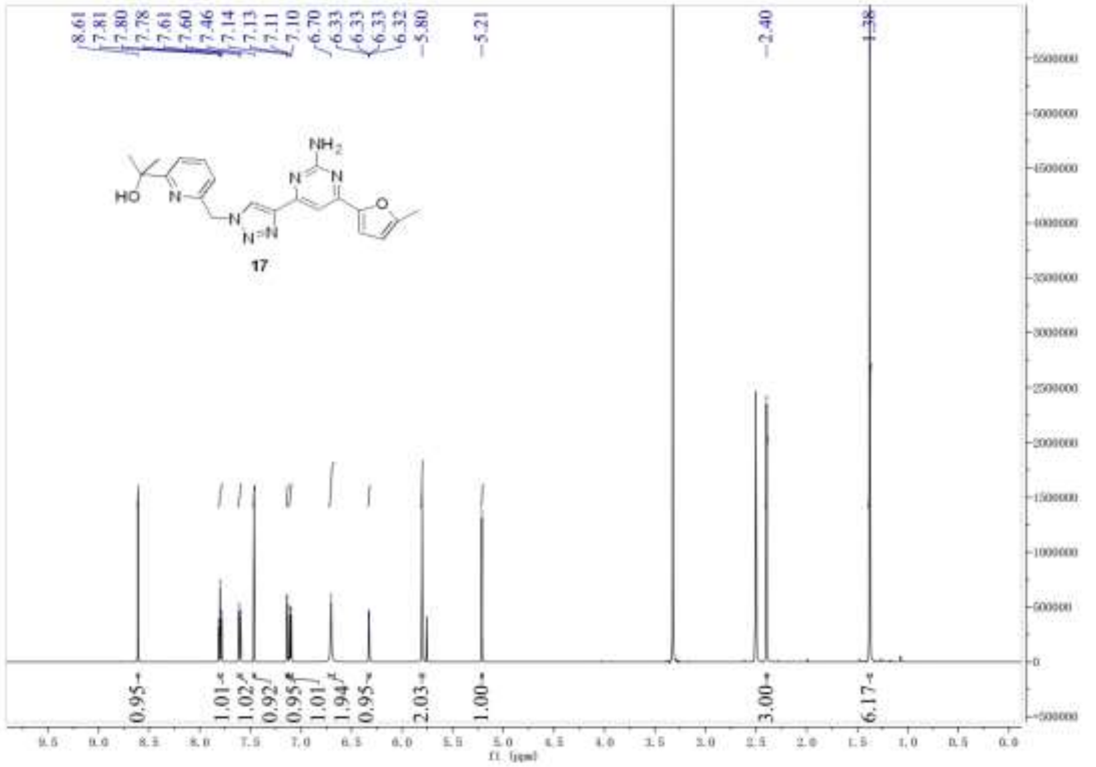

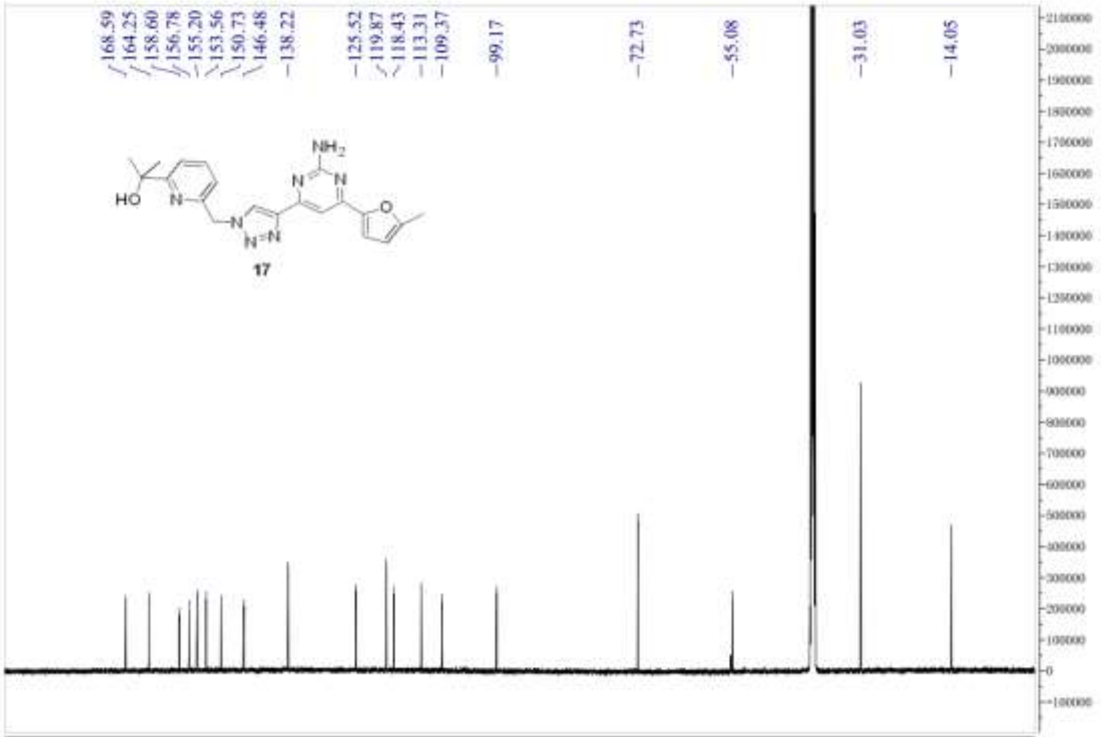

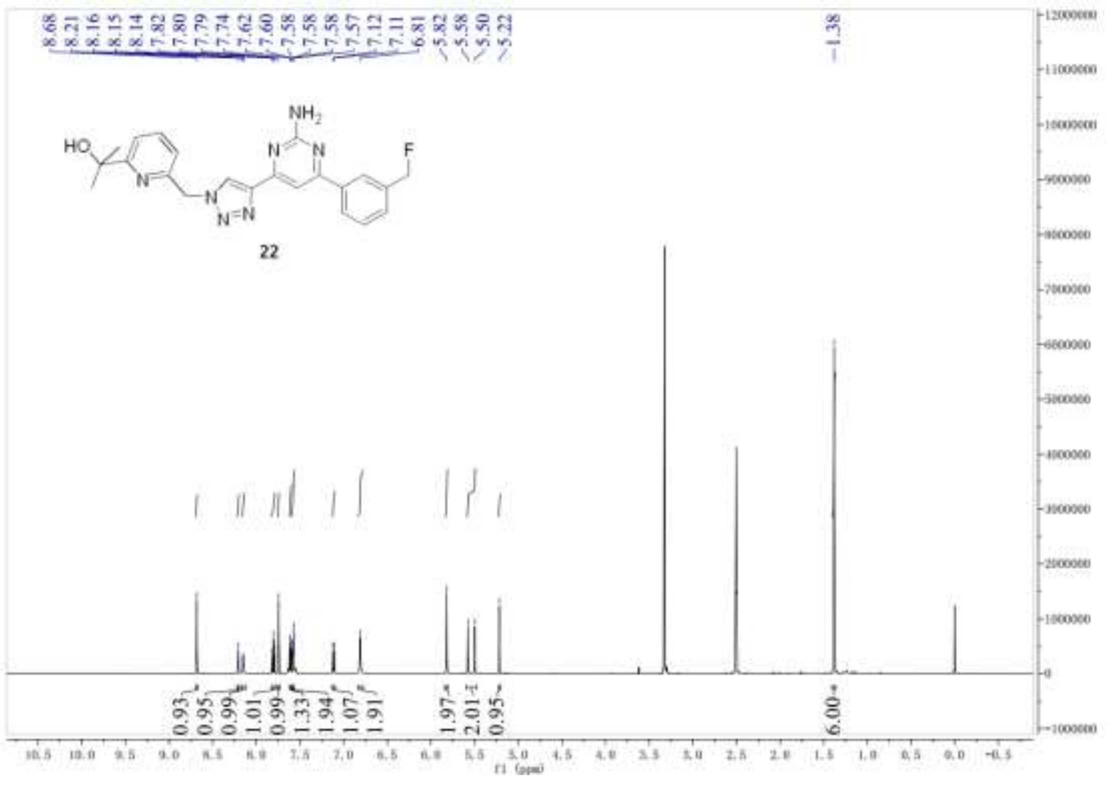

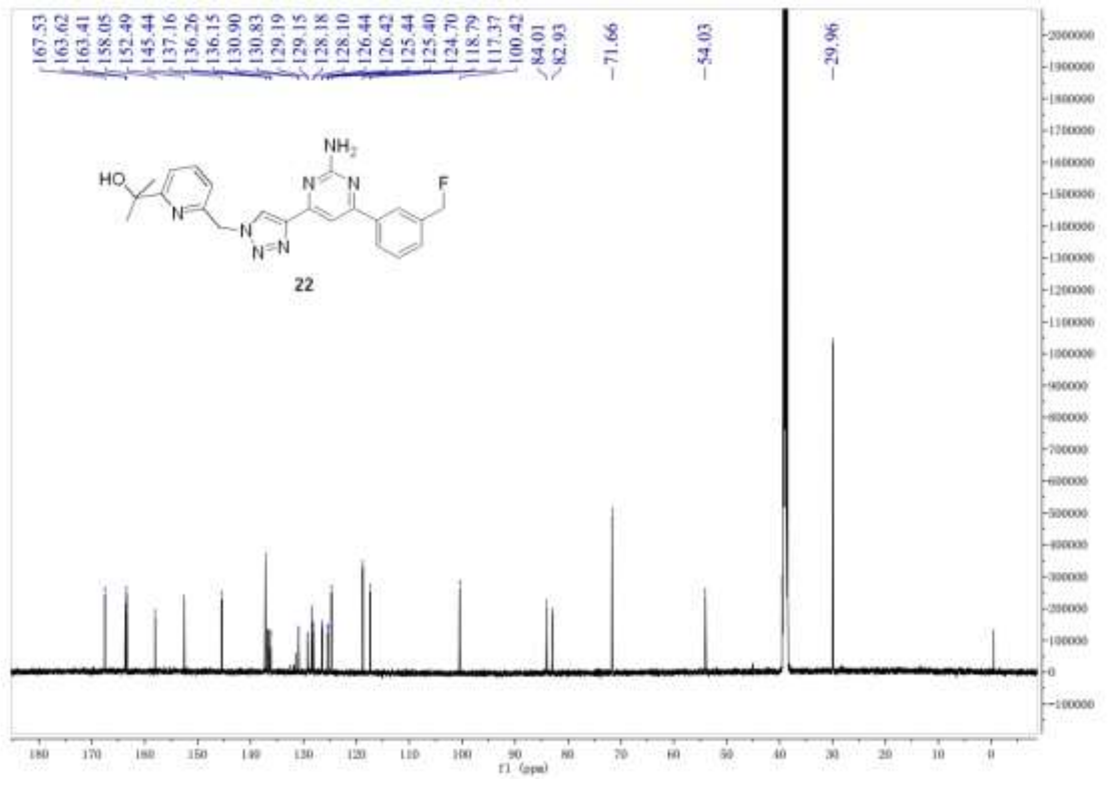

5. Copies of HRMS

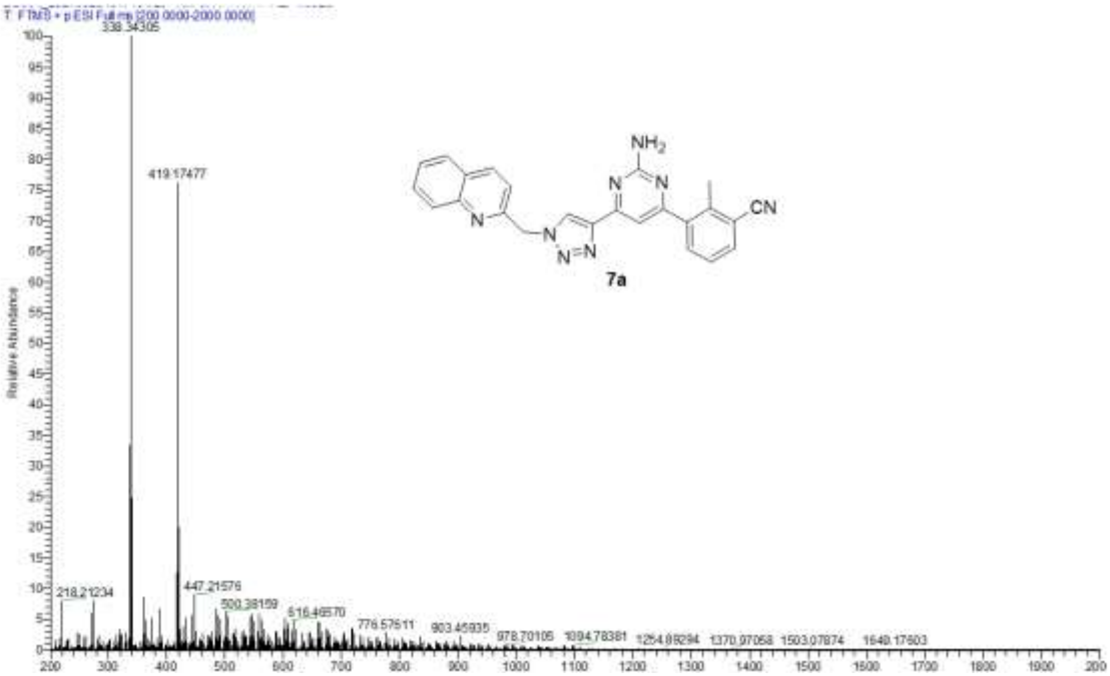

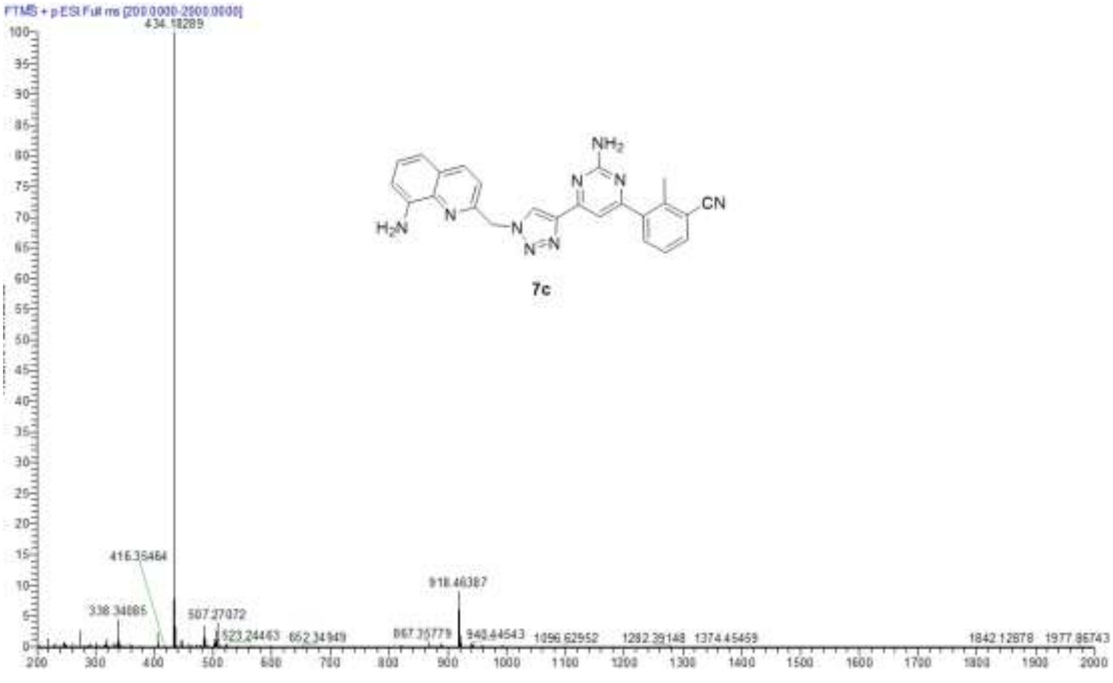

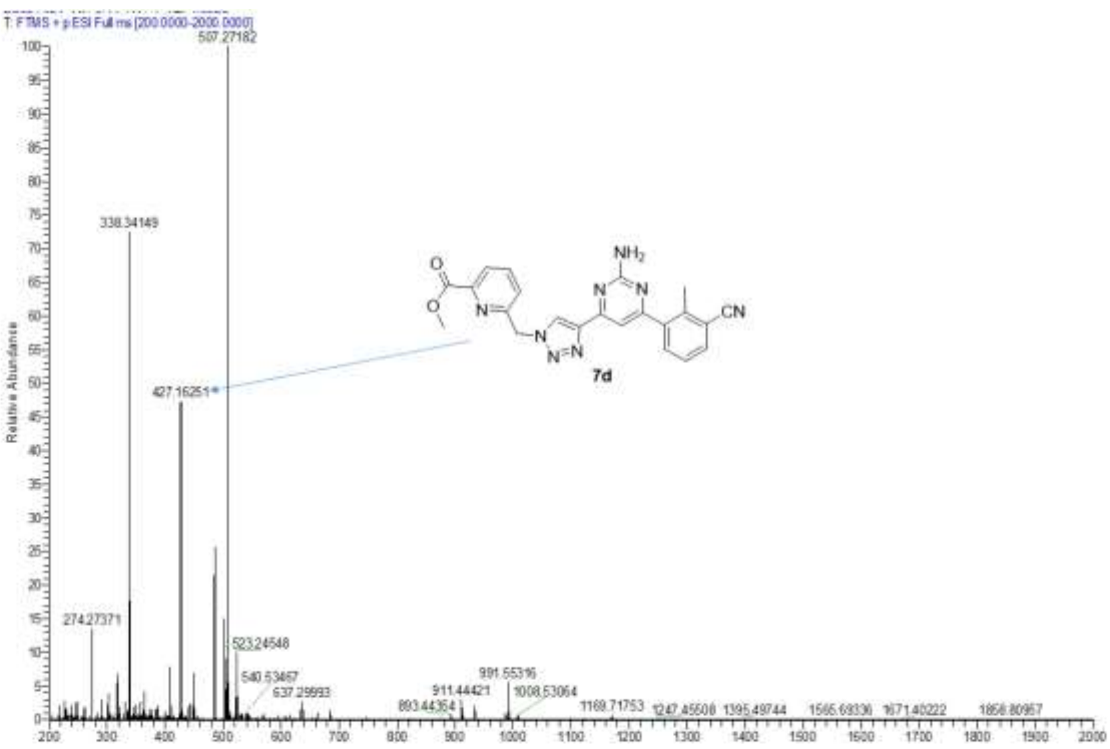
T: FTMS + p ESI Full ms [200.0000-2000.0000]
507.27182
338.34149
427.16251
274.27371
523.24548
540.53467
637.29993
893.44354
911.44421
991.55316
1008.53064
1169.71753
1247.45508
1395.49744
1565.69336
1671.40222
1856.80957
Relative Abundance
NH2
CN
7d

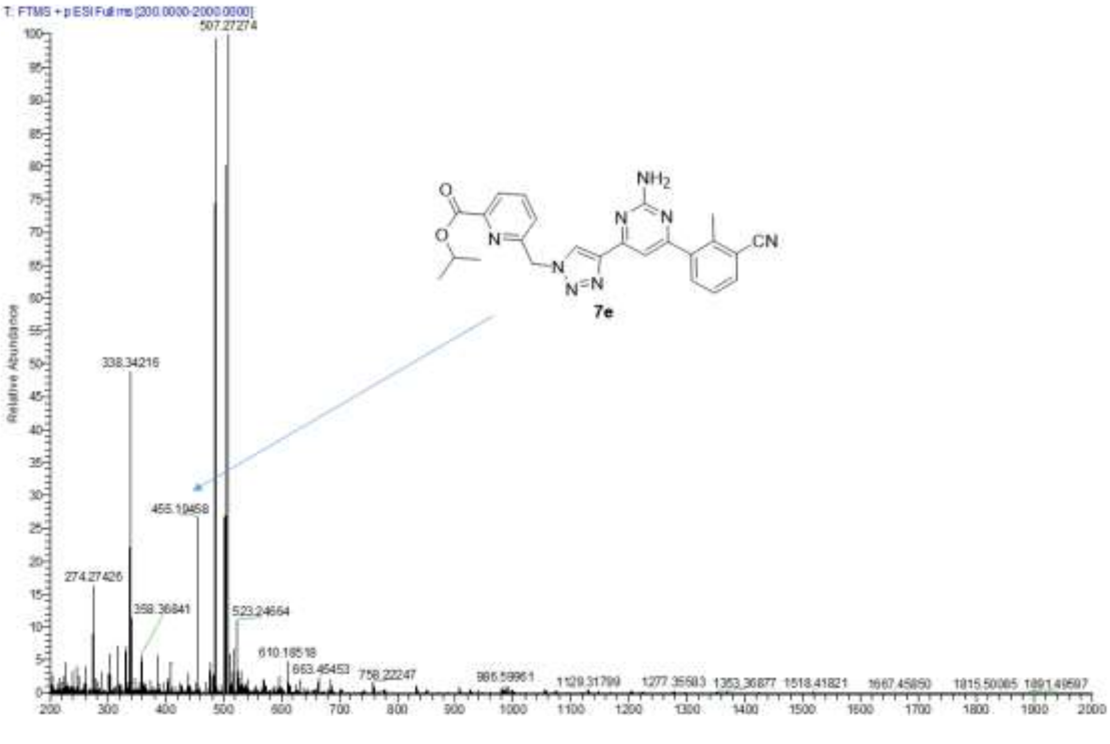
T: FTMS + p ESI Full ms [200.0000-2000.0000]
507.27274
338.34216
455.19458
274.27426
358.36841
523.24664
610.18518
663.45453
758.22247
986.59961
1129.31799
1277.35583
1353.36877
1518.41821
1667.45850
1815.50085
1891.49597
Relative Abundance
NH2
CN
7e

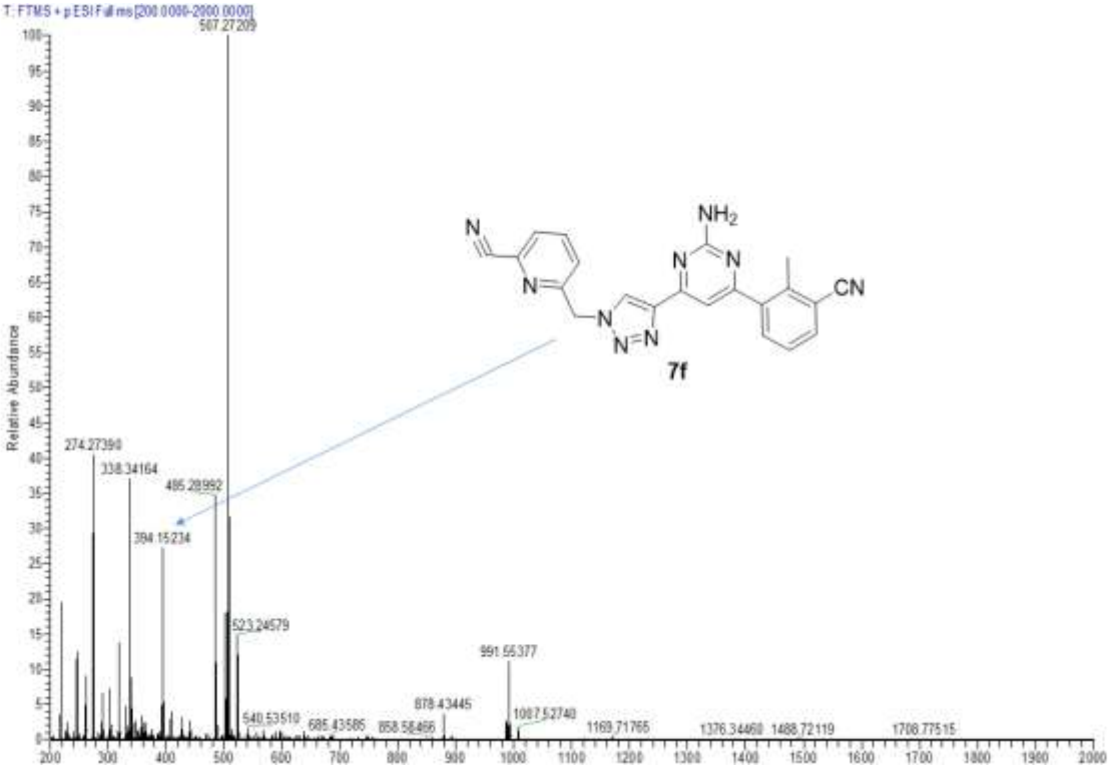
T: FTMS + p ESI Full ms [200.0000-2000.0000]
507.27209
274.27390
338.34164
485.28992
394.15234
523.24579
991.55377
878.43445
1007.52740
540.53510
685.43585
858.58466
1169.71765
1376.34460
1488.72119
1708.77515
Relative Abundance
NH2
CN
7f

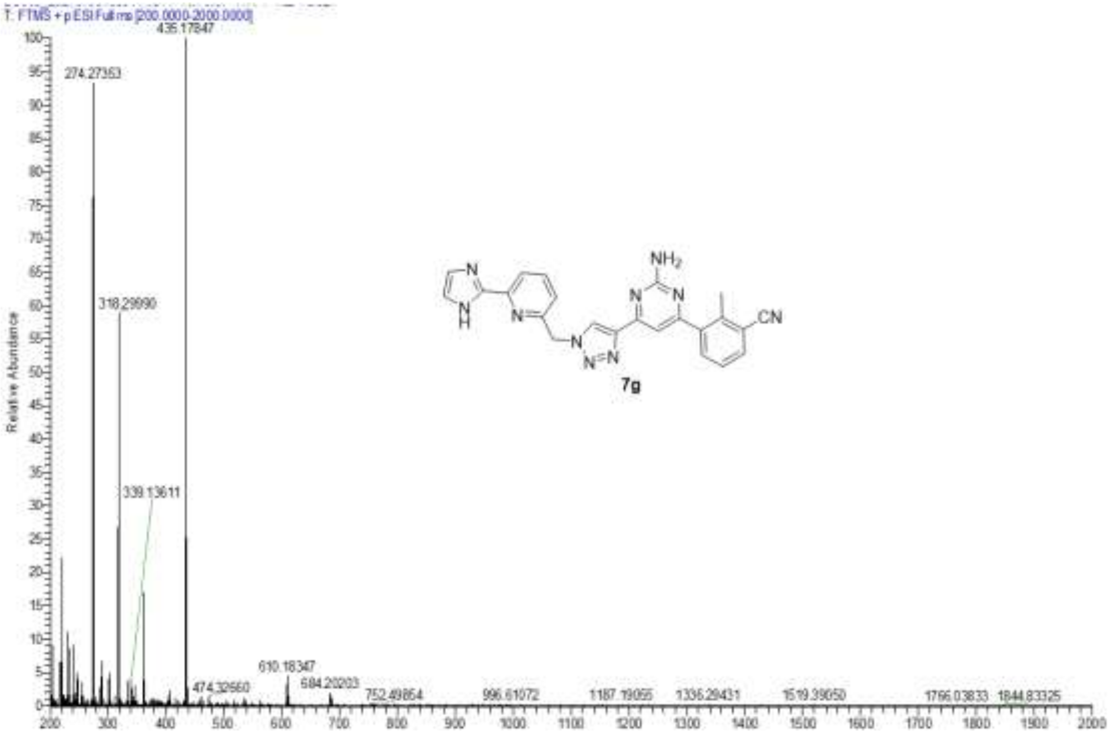
T: FTMS + p ESI Full ms [200.0000-2000.0000]
435.17847
274.27353
318.29990
339.13611
610.18347
684.20203
474.32660
752.49854
996.61072
1187.19055
1336.29431
1519.39050
1766.03833
1844.83325
Relative Abundance
NH2
CN
7g

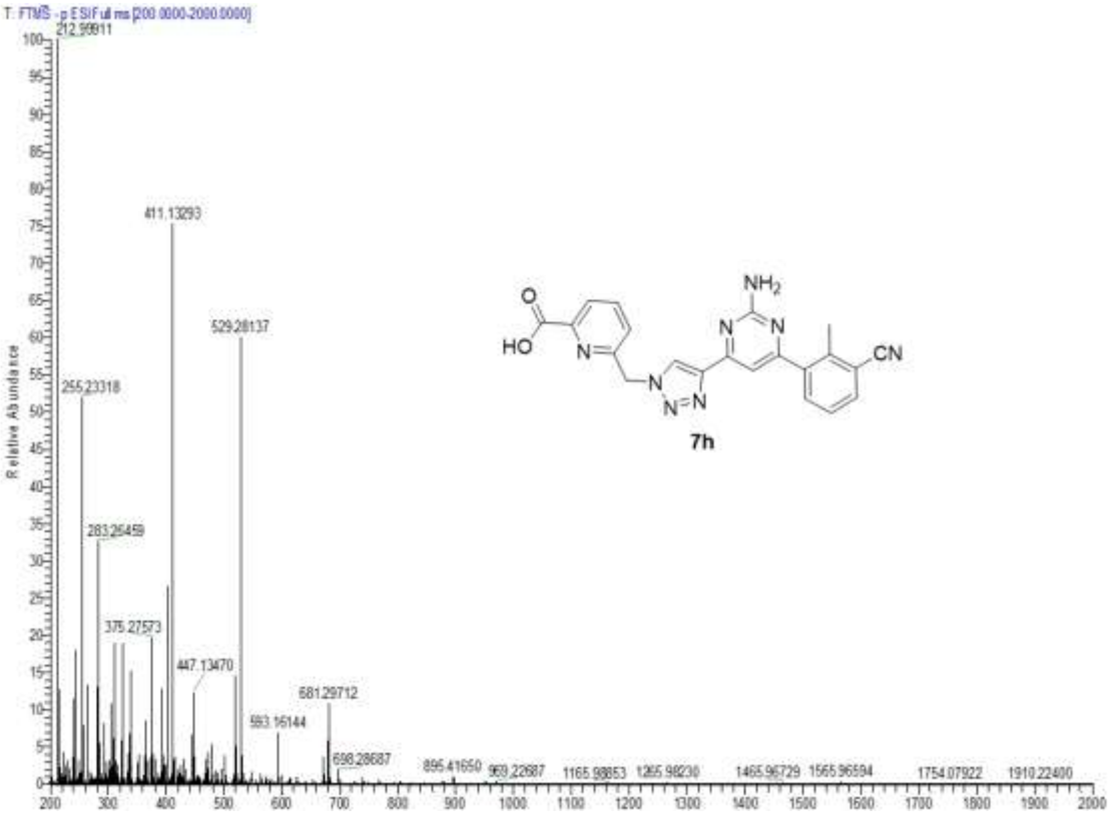
T: FTMS - p ESI Full ms [200.0000-2000.0000]
Relative Abundance
212.99911
411.13293
529.28137
255.23318
283.26459
375.27573
447.13470
681.29712
593.16144
698.28687
895.41650
969.22687
1165.98853
1265.98230
1465.96729
1565.96594
1754.07922
1910.22400
HO
O
N
N
N=N
NH2
N
N
CN
7h

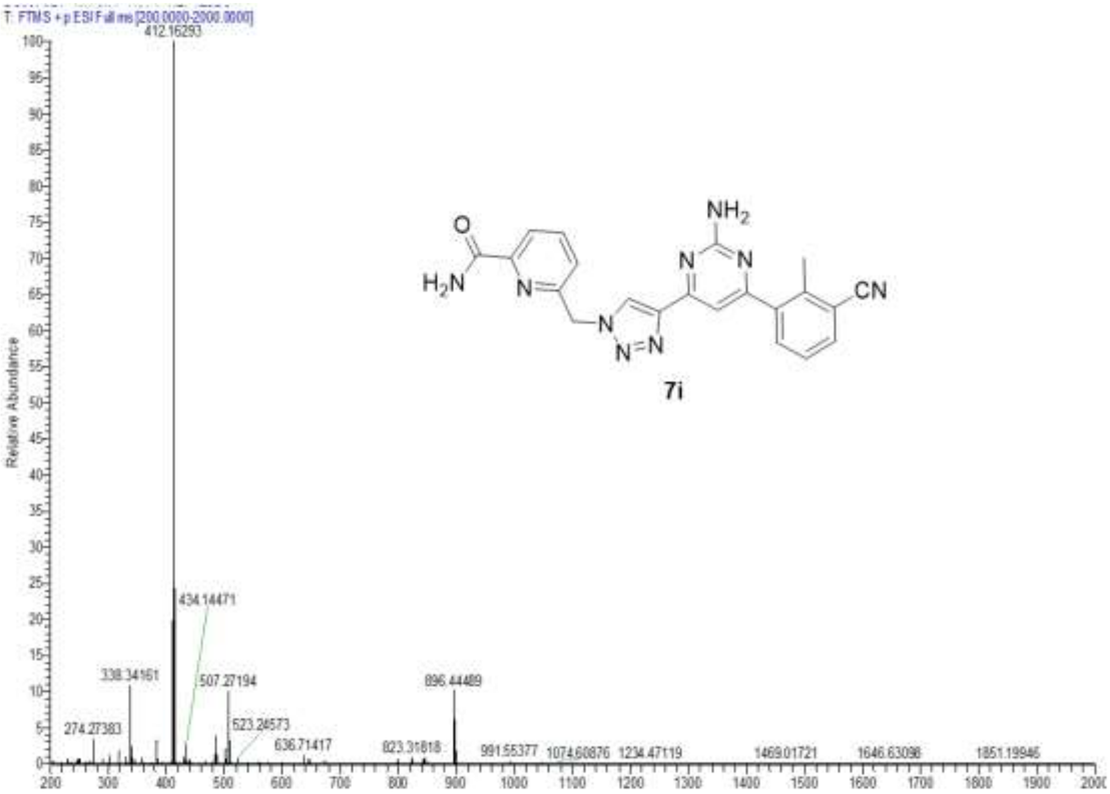
T: FTMS + p ESI Full ms [200.0000-2000.0000]
Relative Abundance
412.16293
434.14471
338.34161
507.27194
896.44489
274.27383
523.24573
636.71417
823.31818
991.55377
1074.60876
1234.47119
1469.01721
1646.63098
1851.19946
H2N
O
N
N
N=N
NH2
N
N
CN
7i

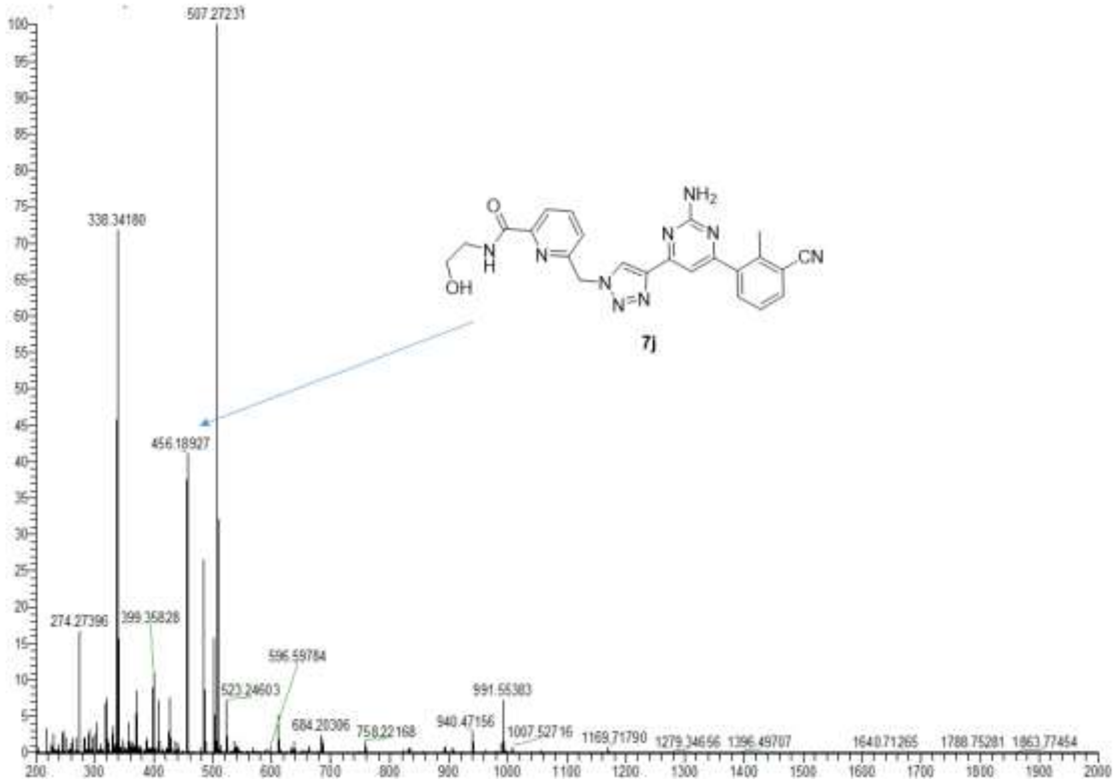
507.27231
338.34180
456.18927
274.27396
399.35828
596.59784
523.24603
991.55383
684.20306
758.22168
940.47156
1007.52716
1169.71790
1279.34656
1396.49707
1640.71265
1788.75281
1863.77454
NH2
O
N
N
N
H
N
CN
OH
N
N
N
7j

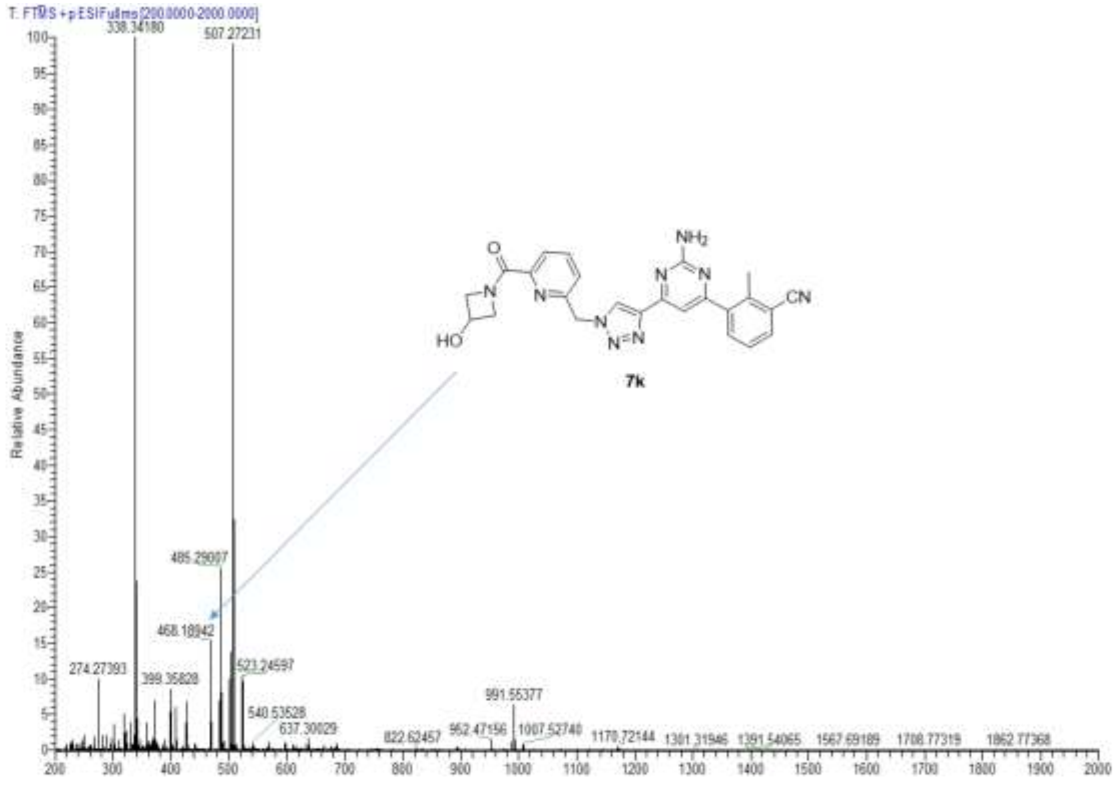
T: FTMS + p ESI Full ms [200.0000-2000.0000]
338.34180
507.27231
Relative Abundance
485.29007
468.18942
274.27393
399.35828
523.24597
540.53528
637.30029
991.55377
822.62457
952.47156
1007.52740
1170.72144
1301.31946
1391.54065
1567.69189
1708.77319
1862.77368
NH2
O
N
N
N
N
CN
HO
N
N
N
7k

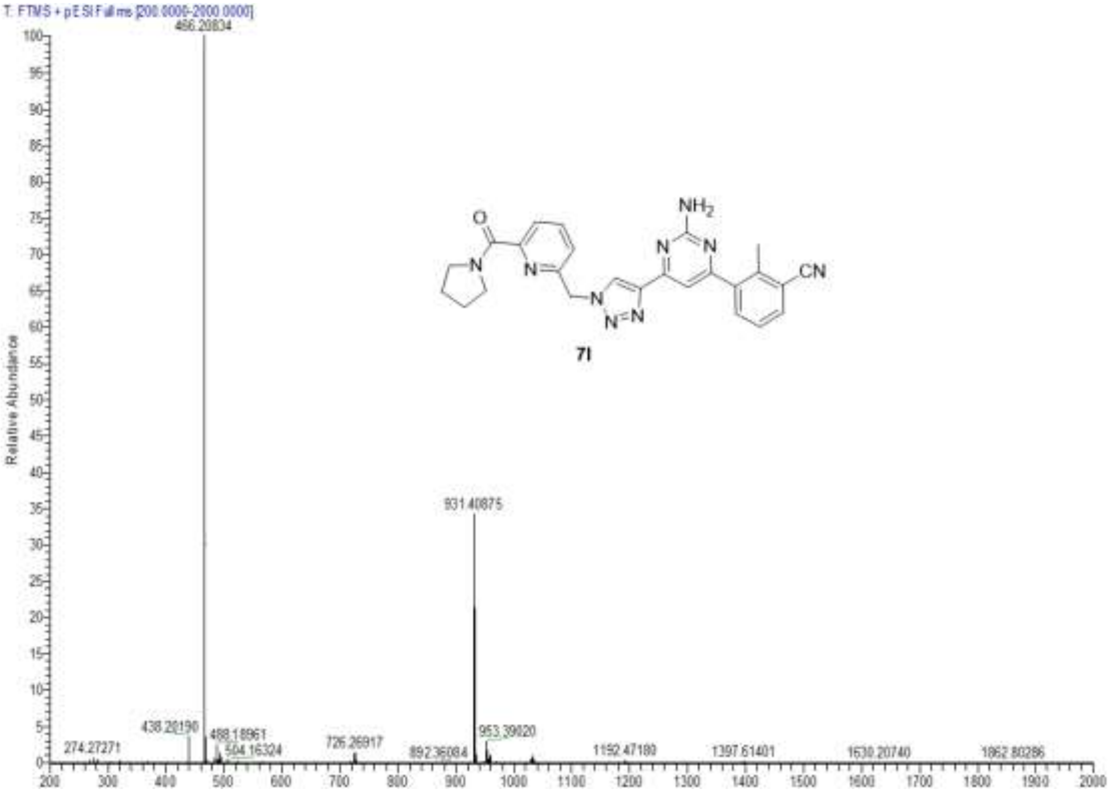
T: FTMS + p ESI Full ms [200.0000-2000.0000]
Relative Abundance
466.20834
931.40875
438.20190
488.18961
504.16324
726.26917
892.36084
953.39020
1192.47180
1397.61401
1630.20740
1862.80286
274.27271

7l

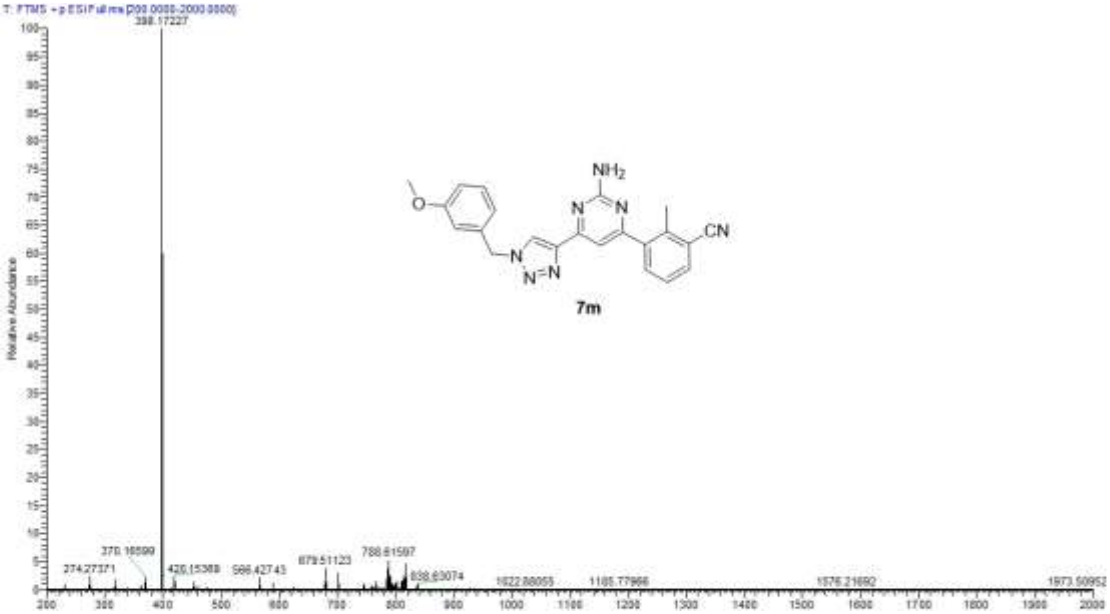
T: FTMS + p ESI Full ms [200.0000-2000.0000]
Relative Abundance
398.17227
370.16599
274.27371
420.15369
566.42743
679.51123
788.61597
838.63074
1022.88055
1185.77966
1576.21692
1973.50952

7m

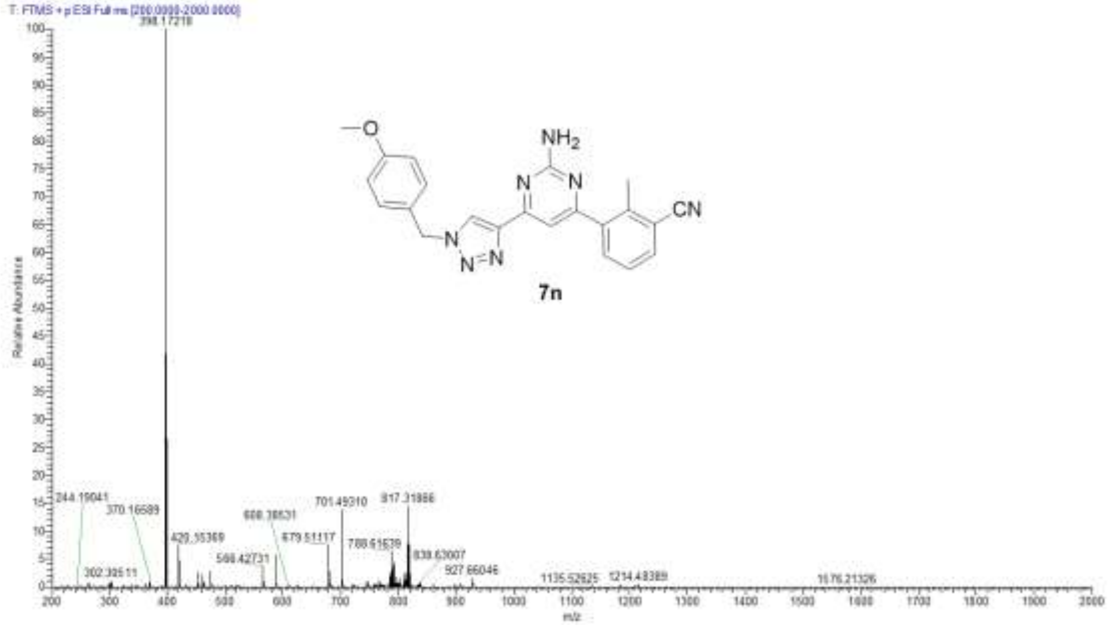
T: FTMS + p ESI Full ms [200.0000-2000.0000]
398.17219
NH2
N
N
CN
N
N=N
7n
Relative Abundance
m/z

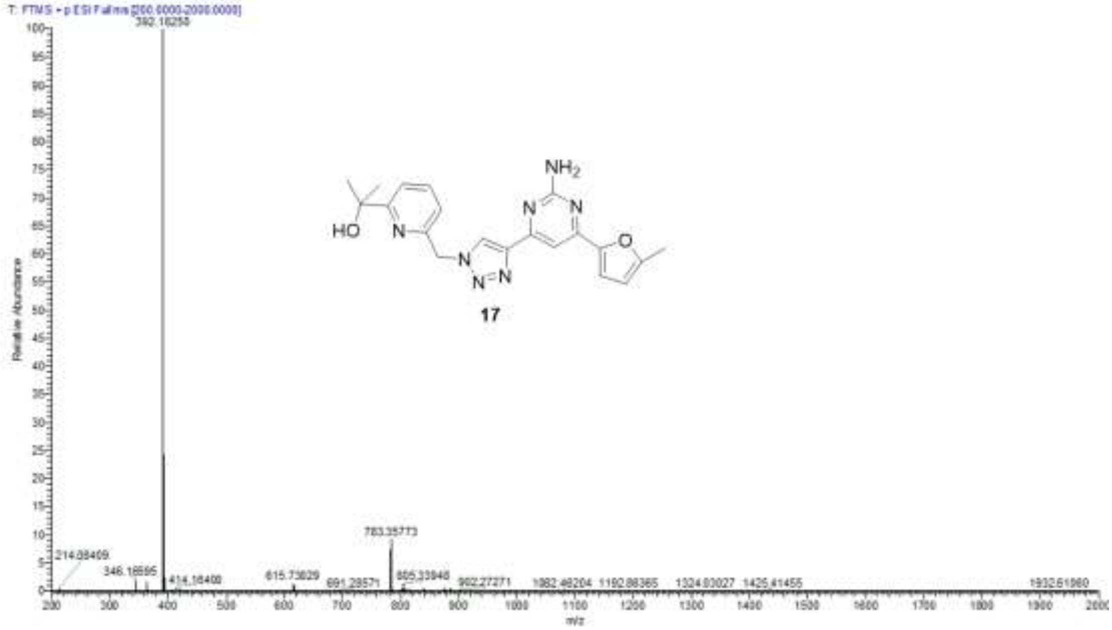
T: FTMS + p ESI Full ms [200.0000-2000.0000]
392.18258
NH2
N
N
HO
N
O
N
N=N
17
Relative Abundance
m/z

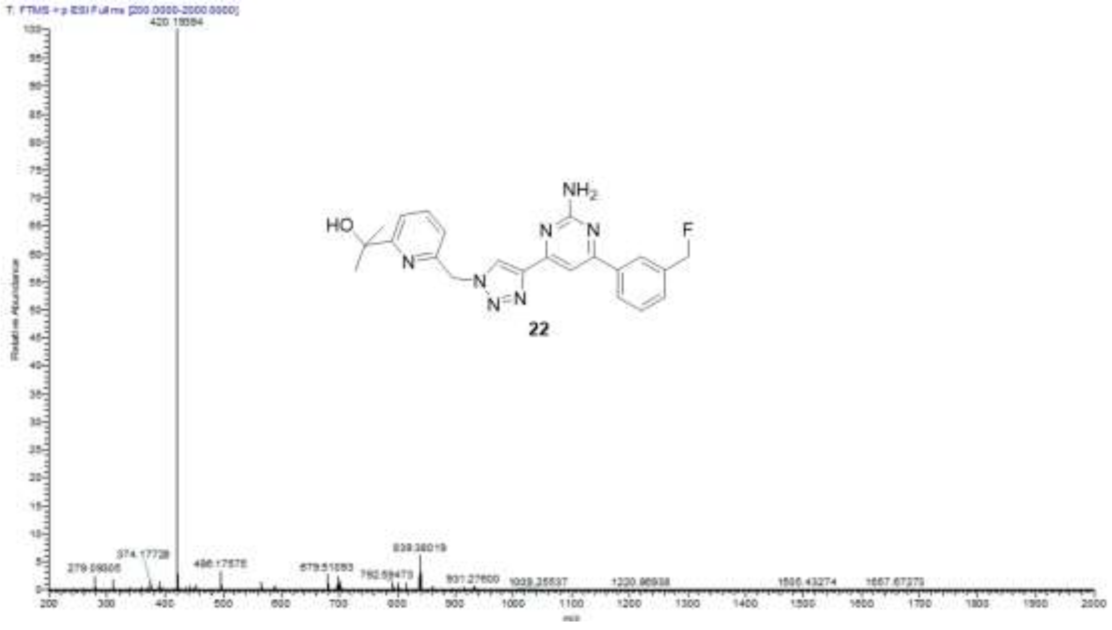

T: FTMS + p ESI Full ms [200.0000-2000.0000]
420.19584
Relative Abundance
HO
N
N
N
N
NH2
N
N
F
22
374.17728
279.09806
496.17575
679.51093
838.38019
931.27600
1039.35537
1220.96938
1506.43274
1667.67279

6. The Ramachandran plot for the homology model of hA_{2B} AR

The primary sequence of hA_{2B} AR was obtained from the NCBI/UNIPROT online database (www.ncbi.nlm.nih.gov/protein/P29275.1) as follows:

1 mlletqdaly valelviaal svagnvlvca avgtantlqt ptnyflvsla aadvavglfa

61 ipfaitislg fctdfygclf lacfvlvltq ssifsllava vdrylaicvp lrykslvtgt

121 rargviavlw vlafgigltp flgwnskdsa tnnctepwdg ttnescclvk clfenvvpms

181 ymvyfnffgc vlppllimlv iyikiflvac rqlqrtelmd hsrttlqrei haakslamiv

241 gifalcwlpv havncvtlfq paqgknkpkw amnmaillsh ansvvnpivy ayrnrdfryt

301 fhkiisryll cqadvksgng qagvqpalgv gl

Based on the primary sequence, the homology model of hA_{2B} AR was built using Swiss Model program from a hA_{2A} AR crystal structure as the template (PDB ID: 6PS7). The sequence identity of homology model and the template was 61.92%. And the homology model has been checked using the Ramachandran plot application in Discovery Studio 2017 R2 (Figure S1). The data showed that 98.2% (326/332) of the residues are in favorable regions and 1.2% (4/332) in permitted regions. These data indicate that the model has high stereochemical quality, since models with more than 90% of residues in favorable and allowed regions are considered excellent and therefore, suitable for molecular docking studies.

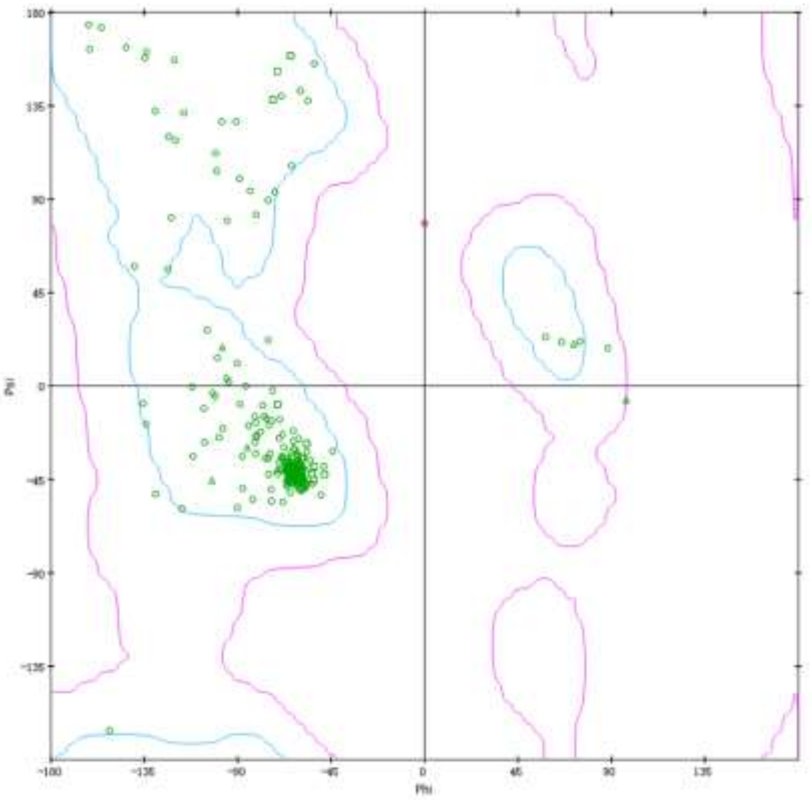

Figure S1. The Ramachandran plot for the homology model of hA_{2B} AR

7. Binding modes of compound 7i with hA_{2A} AR or hA_{2B} AR

Docking studies were performed at the hA_{2A} and hA_{2B} AR binding sites to simulate the interaction of compound **7i** with these two receptors. The binding modes were analyzed by docking simulations using the C-DOCKER protocol of Discovery Studio 2017 R2. The interactions were visualised by Pymol software and Discovery Studio Visualizer (Figure S2 and S3).

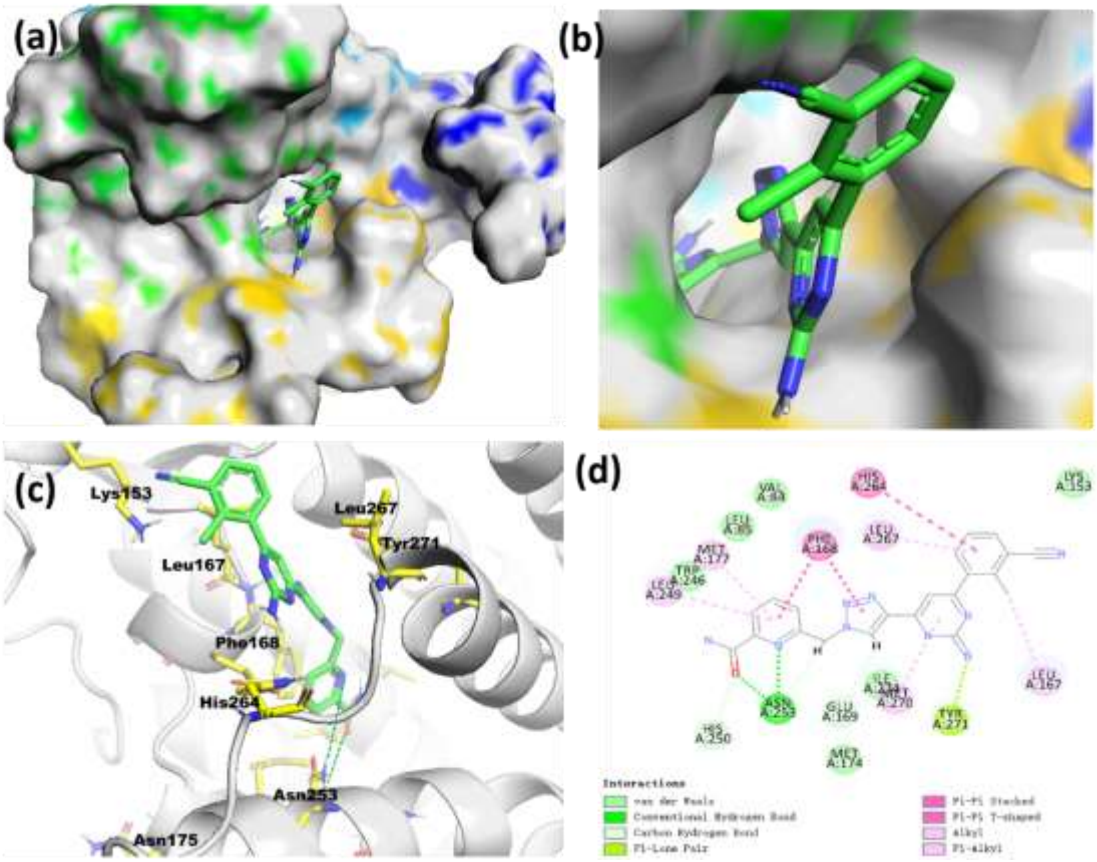

Figure S2. The binding mode of compound **7i** with hA_{2A} AR: (a) the 3D view of **7i** in the active pocket of hA_{2A} AR; (b) **7i** in the active pocket; (c) 3D view of **7i** and key receptor residues; (d) 2D diagrams of ligand-target interactions for **7i** with the A_{2A} AR.

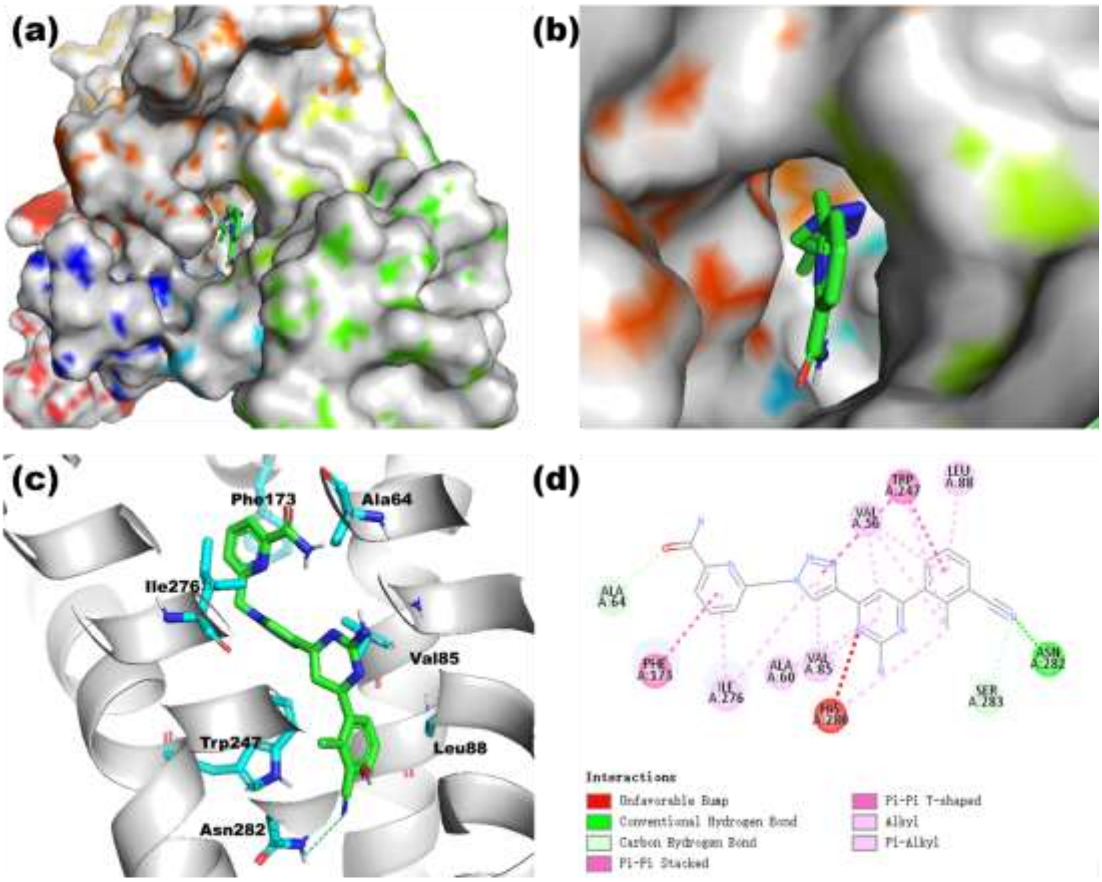

Figure S3. The binding mode of compound **7i** with hA_{2B} AR (homology mode): (a) the 3D view of **7i** in the active pocket of hA_{2B} AR; (b) **7i** in the active pocket; (c) 3D view of **7i** and key receptor residues; (d) 2D diagrams of ligand-target interactions for **7i** with the A_{2B} AR.

8. Metabolic stability in human and rat liver microsomes

8.1 Method

Metabolic stabilities of compounds **7f**, **7i** and AB928 in liver microsomes were studied with pooled human (male) liver microsomes solution (20 mg/mL) and male Sprague-Dawley rats pooled liver microsomes solution (20 mg/mL) purchased from Research Institute for Liver Diseases (Shanghai) Co. Ltd. Both liver microsomes solutions (140 μL) were diluted by 3948 μL of water and 280 μL of phosphate buffered saline. The assay procedure: 640 μL of diluted liver microsomes solution was added to a solution of compound working solution (16 μL, 25 μM in DMSO) and the mixed solution was

incubated at 37 °C for 5 min. Then 160 μL of NADPH generating system was added and take out 100 μL from the mixed solution was added 200 μL of acetonitrile solution to stop the reaction at the set incubation time (0 min, 10 min, 20 min, 30 min, 40 min, 50 min, and 60 min). All stopped reaction solutions were vortex mixed (3 min) and centrifuged (10000 rpm, 4 °C for 10 min). Then 50 μL of the supernatant was analyzed by UPLC-MS/MS system.

8.2 Chromatography and mass spectrometry conditions

UPLC was performed using an ACQUITY^{TM} UPLC system (Waters, Milford, MA, USA). Separation was achieved on a Waters BEH C18 column（100 mm × 2.1 mm，1.7 μm) at 40 °C. The injection volume of the sample solution was 5 μL. The mobile phase consisted of water (0.1% formic acid and ammonium acetate) and acetonitrile solution (20:80, v/v) at a flow rate of 0.4 mL/min within a running time of 6 min. Mass spectrometric analysis was performed on a Waters Xevo TQ-S triple quadrupole instrument using electrospray ionization in positive mode. Quantification was performed using multiple reaction monitoring (MRM) mode to monitor precursor-product ion transitions (Table S1).

Table S1. Conditions of MRM Detection.

Compounds	m/z	DP (volts)	CE (volts)
**7f**	394.100 → 366.100	101	25
**7i**	412.100 → 339.000	75	29
AB928	427.200 → 381.300	70	28
7-Ethoxycoumarin	191.100 → 107.100	85	36

8.3 Raw data

Table S2. Raw data of metabolic stability in human and rat liver microsomes.

Time	%Remaining (7-Ethoxycoumarin)		%Remaining (**7f**)		%Remaining (**7i**)		%Remaining (AB928)	
(min)	Hum	Rat	Hum	Rat	Hum	Rat	Hum	Rat
0	100.00	100.00	100.00	100.00	100.00	100.00	100.00	100.00
10	34.02	49.01	96.32	84.69	98.04	74.94	96.20	74.70
20	11.49	22.97	96.51	87.95	107.96	72.75	103.70	67.40
30	3.54	10.45	107.23	77.21	116.43	67.80	97.00	53.10
40	1.60	2.98	98.42	73.70	100.72	61.04	91.80	42.80
50	0.97	1.02	101.51	64.23	94.99	53.78	89.00	35.60
60	0.72	0.35	104.65	64.04	98.76	50.67	94.40	28.10

9. Analytical method of pharmacokinetics assay

9.1 Animals

Animal experiments were performed according to the institutional ethical guidelines on animal care and approved by the Institute Animal Care and Use Committee at Binzhou Medical University.

Female Bal b/c mice (sourced from Jinan Pengyue Experimental Animal Breeding Co., Ltd.) approximately 6-8 weeks of age, and a weight of approximately 20-30 g. All of the mice were permitted with free access to standard pelleted food and water ad libitum and were kept in clean cages under controlled conditions (light/ dark cycle of 12 h, 60 ± 2% humidity, and 25 ± 1 °C). Before the day of administration, rats were fasted for 12 h but allowed water ad libitum.

9.2 Design of bioavailability study

The mice were divided into 4 groups each having six mice and were collected at crossover time points, with three mice at each point. Pharmacokinetics (PK) was

evaluated after a single dose of 30 mg/kg oral gavage (Oral) and 10 mg/kg intravenous (i.v.) administration. The blood samples were obtained from the suborbital veniplex. Each cohort had three mice, and plasma was collected at pre, 5 min, 15 min, 30 min, 1 h, 2 h, 4 h, 6 h, 8 h, 12 h, and 24 h post dose for intravenous administration. Plasma was collected at pre, 15 min, 30 min, 1 h, 2 h, 4 h, 6 h, 8 h, 12 h, and 24 h post dose for Oral.

9.3 Samples collection and quantitative analysis

Approximately 200 μL of blood was collected at each time point. All blood samples were put into plastic microcentrifuge tubes containing Heparin-Na as anticoagulant. microcentrifuge tubes with blood samples and anticoagulant were inverted several times for proper mixing of the tube contents to centrifugation for plasma. Plasma samples will be centrifuged at 12000 rpm for 8 min at 4 °C to obtain the supernatant. The serum sample (25 μL) was treated with acetonitrile (100 μL), after which the mixture was vortex-mixed for 8 min and centrifuged at 10000 rpm for 10 min at 4 °C. The supernatant layer was collected and then 50 μL of supernatant was injected for the UPLC-MS/MS analysis.

9.4 Chromatography and mass spectrometry conditions

UPLC was performed using an ACQUITY^{TM} UPLC system (Waters, Milford, MA, USA). Separation was achieved on a Waters XBridge column (150 mm × 2.1 mm, 3.5 μm). Column temperature was maintained at 40 °C. The injection volume of the sample solution was 10 μL. The mobile phase consisted of water (0.1% formic acid) and acetonitrile solution (20:80, v/v) at a flow rate of 0.6 mL/min within a running time of 6 min. Mass spectrometric analysis was performed on a Waters Xevo TQ-S triple quadrupole instrument using electrospray ionization in positive mode. Quantification was performed in multiple reaction monitoring (MRM) using the same conditions in Table S1.

9.5 Standard solutions and work curve

Stock solutions of 1.00 mg/mL **7f**, **7i**, and AB928 were prepared in acetonitrile and

stored at -20 ℃. Calibration solutions of **7f**, **7i**, and AB928 from 2.5 to 1000 ng/mL were diluted with mice blank plasma. A one-step protein precipitation was adapted as follows: a 20 μL plasma sample was transferred into a 1.5 mL centrifuge tube, and 100 μL of acetonitrile (including 375 ng/mL IS) was added to precipitate the protein. The samples were vortexed for 8 min, followed by centrifugation at 10000 rpm for 5 min. Then, the supernatants were transferred to an autosampler bottle, and 50 μL of sample was immediately injected into the UPLC-MS/MS system for analysis.
